# Local Energy Decomposition of Intramolecular Interactions: The CovaLED Approach and Its Application to Molecular Recognition in Biomolecular Assemblies

**DOI:** 10.1021/acscentsci.6c00336

**Published:** 2026-05-27

**Authors:** Martina Colucci, Christoph Riplinger, Ahmet Altun, Frank Neese, Giovanni Bistoni

**Affiliations:** † Department of Chemistry, Biology and Biotechnology, University of Perugia, 06123 Perugia, Italy; ‡ FAccTs GmbH, Rolandstr. 67, 50677 Köln, Germany; § 28314 Max-Planck-Institut für Kohlenforschung, Kaiser-Wilhelm-Platz 1, 45470 Mülheim an der Ruhr, Germany

## Abstract

Achieving quantitative insight into the noncovalent interactions that govern molecular function and biological activity in complex assemblies remains a major challenge for quantum chemical analysis, particularly in systems such as nucleic acids where standard force-fields are known to struggle. Local energy decomposition (LED) provides gold-standard coupled-cluster descriptions of intermolecular interactions but can become difficult to interpret chemically when interacting fragments are covalently connected, such as ligands embedded in nucleic acid frameworks or functional groups linked through bonding networks. In these situations, shared electron density across fragment boundaries obscures the physical interpretation of energy contributions. Here we introduce CovaLED, an extension of the LED scheme that enables a rigorous treatment of covalent connectivity within the LED framework. Application to nucleic-acid-based recognition systems demonstrates the capabilities of the approach. In a riboswitch RNA–ligand complex, CovaLED reveals how methylation of guanine leads to a loss of stabilizing hydrogen-bonding interactions, consistent with experimental binding affinity trends. In a segment of human DNA, the method enables accurate quantification of interactions between nucleotides covalently linked within the backbone. CovaLED thus enables coupled-cluster-level energy decomposition in realistic biomolecular systems, where covalent structure and noncovalent recognition are intrinsically intertwined.

## Graphical abstract

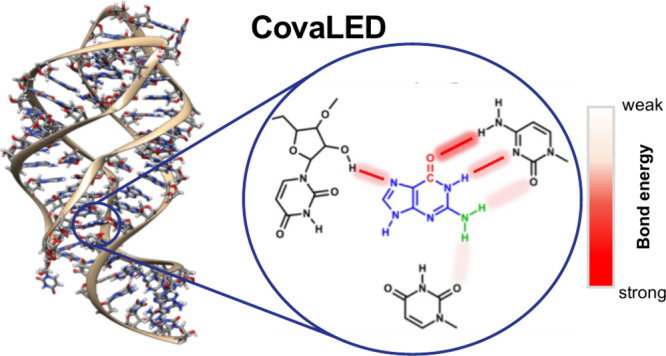

## Introduction

The ability of biomolecules to recognize and bind specific partners underpins gene regulation, enzymatic catalysis, and therapeutic action.^1–3^ Yet, despite decades of research, the challenge of understanding molecular recognition remains multifaceted. At the most basic level, we must establish reliable structural models of the complexes involved. Beyond this, we need to identify the key forces that determine stability and selectivity. But the true difficulty lies in moving from description to prediction: anticipating how a point mutation alters protein–ligand affinity, why a chemically subtle ligand modification disrupts RNA binding, or how competing interactions within a crowded cellular environment shift recognition outcome. Meeting these challenges requires methods that can both access biologically relevant scales and provide a transparent, quantitative account of the physics behind binding.

On the structural side, Artificial Intelligence (AI) methods now deliver models of proteins, nucleic acids, and their complexes at near–experimental resolution.^4–6^ Yet their strength is also their limitation: while AI excels at answering *what* a biomolecular structure might be, it cannot explain *why* molecules recognize each other, nor which physical forces dictate selectivity and stability. While many sophisticated interpretable models are available,^7^ the highly complex architectures that underlie state-of-the-art predictors - such as deep neural networks - still obscure the underlying physical drivers of binding.

Molecular recognition can also be investigated using physics-based computational approaches such as molecular docking, molecular dynamics (MD) simulations, and free-energy calculations.^8^ These methods are grounded in statistical mechanics and have therefore played a central role in developing a quantitative understanding of binding thermodynamics and kinetics. More recently, machine-learning techniques have been integrated with these frameworks to extend their practical applicability.^9^ However, the predictive reliability of such strategies remains strongly dependent on the quality of the underlying force fields. In systems where these models are insufficiently accurate, their errors propagate directly into computed affinities and selectivities. RNA provides a notable example: despite substantial progress, the transferability and balance of current RNA force fields remain under active scrutiny, which limits the robustness of quantitative predictions for RNA–ligand recognition.^10^

For decades, quantum chemistry has been regarded as the most rigorous route to understanding molecular recognition, since in principle it can deliver a quantitative description of all the forces that stabilize biomolecular assemblies. The difficulty lies in the complexity of the underlying problem: the many-electron Schrödinger equation has no closed-form solution, and accurate models to approximate it - such as coupled-cluster theory - inevitably scale steeply with system size.^11^ As a consequence, direct applications to large biomolecular assemblies, including proteins and nucleic acids, have long remained impractical. To extend the reach of quantum chemistry, researchers have therefore turned to more approximate approaches, such as semiempirical methods and density functional theory (DFT). While these methods are still substantially more demanding than classical force fields, they enable simulations of larger systems. In addition, because the wave function at the mean-field level has a rather simple formal structure, being described by a single Slater determinant, the formulation of energy decomposition schemes within these methods is relatively straightforward from both a mathematical and an implementation standpoint.^12^ In such approaches, interaction energies computed at the Hartree–Fock (HF) or DFT level can be partitioned into components such as electrostatics, exchange, induction, and dispersion, thereby expressing the physics of binding in chemically intuitive terms. This gain in simplicity, interpretability, and computational tractability, however, comes at the cost of reduced and less systematically controllable accuracy compared to correlated wave function-based methods.

Recent progress in local correlation methods has changed the landscape.^13–15^ By exploiting the intrinsic short-range nature of electron correlation, local coupled-cluster approaches reduce the steep scaling of canonical wave function methods and make it possible to compute highly accurate interaction energies for proteins and other large assemblies on a routine basis. Accuracy, however, comes at the cost of complexity. The resulting wave function in this case is a mathematical object defined by billions of cluster amplitudes encoding correlation effects at the cost of losing chemical interpretation. Precisely because correlated wave functions are so information-rich yet chemically opaque, the need for physically meaningful interpretive frameworks becomes even more pressing at this level of theory than at the mean-field level.^16–19^ The central challenge is therefore clear: to develop energy decomposition schemes that are firmly rooted in modern local coupled cluster approaches. The local energy decomposition (LED),^20–24^ rooted in the domain-based pair natural orbital (DLPNO) framework,^14^ provides a direct route to this goal, enabling coupled-cluster-level analysis of the forces governing recognition in intact biomolecular assemblies.

LED has already been applied to biomolecular assemblies, but only by introducing significant approximations. A central limitation in this context is the need to assign orbitals to user-defined fragments, a procedure that becomes ambiguous in systems connected by covalent bonds. To define fragments under such conditions, studies have typically relied on model systems, for example dividing the system into smaller subunits that interact noncovalently and introducing link atoms to saturate the resulting dangling bonds.^25,26^ Similar strategies have also been employed in combination with multilayer approaches.^27^ Alternatively, covalent bonds have been cut heterolytically, thereby creating artificially charged fragments.^28^ While these strategies enabled studies of large assemblies, they did so at the expense of chemical realism, since covalent connectivity and environmental effects were not treated on the same footing as noncovalent interactions. In this work, we introduce the CovaLED framework, which overcomes this limitation by extending LED to treat covalent bonds explicitly. This makes it possible to partition a biomolecular system into fragments without modifying its structure or introducing artificial charges, and to analyze intra- and intermolecular interactions within the intact system in a chemically consistent way. The result is a method that preserves coupled-cluster accuracy while providing an interpretable decomposition of all contributions to molecular recognition, from strong covalent effects to the subtle noncovalent interactions that determine stability and selectivity.

As a case study, we focus on the guanine riboswitch, a paradigmatic system for RNA-ligand recognition.^29–31^ This riboswitch regulates gene expression by binding guanine with nanomolar affinity, exploiting a binding pocket that is almost completely solvent-inaccessible and engages the ligand through a dense hydrogen-bonding network. Even subtle chemical modifications can drastically perturb this recognition process. For example, replacing guanine with O6-methylguanine imposes a binding penalty of more than 3 orders of magnitude relative to the natural ligand.^32^ Such modifications not only weaken binding affinity but also alter the regulatory response of the riboswitch, as observed in transcription termination assays. The structures of the guanine- and O6-methylguanine–riboswitch complexes thus provide an ideal benchmark for probing how small chemical changes translate into profound energetic and functional consequences. By enabling coupled-cluster-level decomposition of RNA-ligand interactions, our approach not only reproduces the experimental difference in binding affinity but also unveils its physical origin. Specifically, we can disentangle how individual forces - hydrogen bonding, stacking, electrostatics, and dispersion - are rebalanced upon ligand modification, and how these changes manifest in the observed energetic penalty. Beyond demonstrating technical feasibility, this application highlights the broader impact of the method: the ability to resolve the fundamental physical forces that differentiate a natural ligand from a close analog in an RNA system of direct biological and therapeutic relevance.

## Results and Discussion

### The CovaLED Approach

The LED scheme partitions a system into fragments and decomposes the total DLPNO–CCSD­(T) energy into intrafragment and interfragment contributions by exploiting the locality of occupied and virtual orbitals in the DLPNO framework. Each orbital is assigned to the fragment on which it is predominantly localized. Interfragment contributions can further be resolved into physically meaningful components, such as electrostatic, exchange, and dispersion interactions, while intrafragment terms provide insight into short-range effects, including Pauli repulsion, through comparison with the energies of the isolated fragments at the same geometry.

Difficulties arise when fragments are connected by covalent bonds. A covalent bond implies the presence of at least one orbital delocalized over both fragments. In the conventional LED treatment, such an orbital is assigned entirely to one fragment, leading to an inherently “ionic” or “heterolytic” description of the shared electronic density. This leads to strong electrostatic interactions between fragments and might obscure the chemical interpretation of the bonding. To overcome this limitation, the CovaLED approach was developed to provide a more chemically meaningful description of interactions in systems containing covalently connected fragments. In this framework, a shared orbital is defined as an orbital that is not predominantly localized on a single fragment but instead exhibits significant population on two fragments, as determined by orbital population analysis. No explicit population threshold is used to define shared orbitals. Instead, the user specifies the pair of atoms forming the covalent bond to be treated within CovaLED, and the corresponding shared orbitals are identified as those most delocalized between these atoms. Since the current implementation is restricted to the cleavage of a single bond, this identification is straightforward and effectively independent of the specific population analysis scheme, thereby avoiding the introduction of arbitrary parameters.

Let |*k*⟩ be the shared orbital between the fragments *x* and *y*. In the CovaLED framework, we write

$$\left|k\right\rangle =n_{k_{x}}\left|k_{x}\right\rangle +n_{k_{y}}\left|k_{y}\right\rangle$$ 1

where |*k*_*x*_⟩ and |*k*_*y*_⟩ are identical to |*k*⟩ but formally assigned to *x* and *y*, respectively. The coefficients *n*_*k*_*x*__ and *n*_*k*_*y*__ are positive numbers satisfying *n*_*k*_*x*__ + *n*_*k*_*y*__ = 1 and quantify the relative importance of each fragment to the description of |*k*⟩. For a purely covalent bond *n*_*k*_*x*__ = *n*_*k*_*y*__ = 0.5. For polar bonds, fragment-resolved population analyses of orbital |*k*⟩ may instead be used to determine asymmetric coefficients. However, enabling bond polarization in this way introduces partial fragment charges and generally leads to stronger electrostatic interaction terms. Moreover, the presence of charged fragments makes the results more sensitive to the specific population scheme employed, potentially reducing the robustness and transferability of the method. For these reasons, homolytic splitting is used as the default choice in CovaLED.

Starting from the LED expressions of the HF and correlation energies reported in the Supporting Information (eqs S4–S7), the key contributions in the CovaLED framework can be reformulated as follows:

$$E_{x}^{HF}=\underset{A_{x}<B_{x}}{\sum }\frac{Z_{A_{x}}Z_{B_{x}}}{\left|R_{A_{x}}-R_{B_{x}}\right|}-\underset{i_{x}}{\sum }n_{i_{x}}\left\langle i_{x}\right|\nabla_{i_{x}}^{2}\left|i_{x}\right\rangle -2\underset{i_{x},A_{x}}{\sum }n_{i_{x}}\left\langle i_{x}\right|\frac{Z_{A_{x}}}{r_{i_{x}}-R_{A_{x}}}\left|i_{x}\right\rangle +4\underset{i_{x}\leq j_{x}}{\sum }\frac{n_{i_{x}}n_{j_{x}}}{1+\delta_{i_{x}j_{x}}}\left(i_{x}i_{x}|j_{x}j_{x}\right)-2\underset{i_{x}\leq j_{x}}{\sum }\frac{n_{i_{x}}n_{j_{x}}}{1+\delta_{i_{x}j_{x}}}\left(i_{x}j_{x}|j_{x}i_{x}\right)$$ 2

$$E_{xy}^{HF}=\underset{A_{x}<B_{y}}{\sum }\frac{Z_{A_{x}}Z_{B_{y}}}{\left|R_{A_{x}}-R_{B_{y}}\right|}-\underset{i_{x},A_{y}}{\sum }n_{i_{x}}\left\langle i_{x}\right|\frac{Z_{A_{y}}}{r_{i_{x}}-R_{A_{y}}}\left|i_{x}\right\rangle -\underset{i_{y},A_{x}}{\sum }n_{i_{y}}\left\langle i_{y}\right|\frac{Z_{A_{y}}}{r_{i_{y}}-R_{A_{x}}}\left|i_{y}\right\rangle +\underset{i_{x}\leq j_{y}}{\sum }n_{i_{x}}n_{j_{y}}\left(i_{x}i_{x}|j_{y}j_{y}\right)-\frac{1}{2}\underset{i_{x}\leq j_{y}}{\sum }n_{i_{x}}n_{j_{y}}\left(i_{x}j_{y}|j_{y}i_{x}\right)$$ 3

$$E_{x}^{C}=\underset{i_{x}\leq j_{x}}{\sum }n_{i_{x}}n_{j_{x}}\varepsilon_{i_{x}j_{x}}$$ 4

$$E_{xy}^{C}=\underset{i_{x}<j_{y}}{\sum }n_{i_{x}}n_{j_{y}}\varepsilon_{i_{x}j_{y}}$$ 5

where *E*_*x*_^*HF*^ and *E*_*x*_^*C*^ denote intrafragment mean-field and correlation energies of fragment x, respectively, while *E*_*xy*_^*HF*^ and *E*_*xy*_^*C*^ denote the corresponding interfragment contributions. The sums run over all occupied spin orbitals (shared ones appear twice, one for each fragment assignment, e.g. |*k*_*x*_⟩and |*k*_*y*_⟩). Note that, for a given shared orbital |*k*⟩, the corresponding self-interaction terms involving |*k*_*x*_⟩ and |*k*_*y*_⟩ are not included in the HF energy expressions. The quantities *ε*_*ij*_ denote pair correlation energies. For nonshared occupied orbitals, *n*_*k*_*x*__ and *n*_*k*_*y*__ are set to 1. Hence, if no shared orbitals are present, the expressions reduce to those of the standard LED case.

### An Illustrative Case Study: The Ethane-Na^+^ Case

The ethane-Na^+^ system provides a useful test case to illustrate the application of the CovaLED method. The system was divided into three fragments, by cutting the covalent bonds between the carbon atoms in the ethane molecule. The interaction energy between ethane and Na^+^ was computed using a supramolecular approach, yielding −7.4 kcal mol^–1^. This interaction energy was decomposed into contributions from individual Na^+···^CH_3_ and CH_3_^···^CH_3_ interactions using the standard LED (Figure 1A) and CovaLED (Figure 1B) approaches, each in their fragment-pairwise formulation.^33^ As discussed in detail in the Supporting Information and in ref 33, the fragment pairwise formulation is based on the redistribution of the intrafragment terms (electronic preparation energies) among the interfragment terms, in order to obtain an exact decomposition of the binding energy into pairwise contributions.

**Figure 1:**
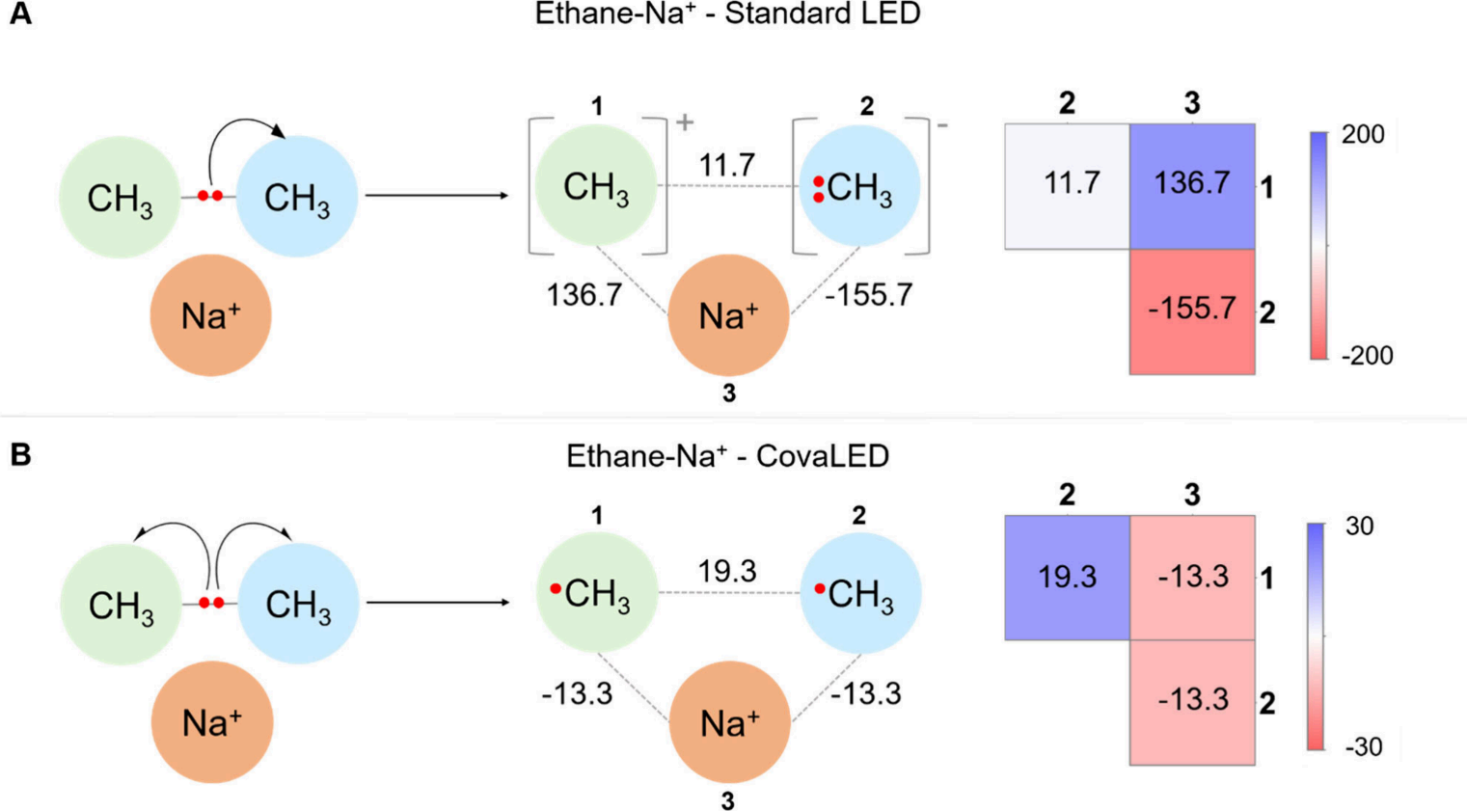
Schematic representation of the ethane-Na^+^ system, showing the assignment of electron density to the fragments and the corresponding fragment-pairwise interaction maps, obtained with (A) standard LED approach and (B) CovaLED method. In both cases, the total interaction energy is −7.4 kcal mol^–1^. All calculations were performed at the DLPNO–CCSD­(T)/CPS/CBS level, as detailed in the Computational Details section. Additional data demonstrating the basis set dependence of the LED energy components for the ethane–Na^+^ system are provided in Table S1 of the Supporting Information.

The fragment-pairwise-LED (fp-LED) map (Figure 1A) shows that the standard LED scheme can yield interaction energies that are difficult to interpret chemically when fragments are connected by a covalent bond. This issue arises from the different formal charges assigned to the two methyl groups, which leads to two strong Na^+^-CH_3_ interactions of opposite sign. In contrast, within the CovaLED framework, the electron density of the covalent bond between the methyl groups is partitioned equally (Figure 1B). As a result, both fragments undergo the same degree of polarization induced by the positively charged Na^+^, yielding identical Na^+^···CH_3_ interaction energies of – 13.3 kcal mol^–1^. The fp-LED map in Figure 1B therefore provides a chemically consistent description of all interactions in the system. Importantly, the covalent bond between the methyl groups is present in both the dimer and the isolated monomers. The value of 19.3 kcal mol^–1^ reported in the map thus reflects the extent to which this bond is weakened upon interaction with Na^+^. In contrast, interactions between fragments belonging to different monomers (the Na^+^···CH_3_ interactions in the present example) arise exclusively from their association and are therefore present only in the dimer. This example illustrates how the CovaLED approach yields a chemically meaningful energy decomposition even in small molecular systems, providing a foundation for its application to larger and more complex biomolecular assemblies.

### The Interaction of DNA Filaments in Human DNA

As a first biological application of the CovaLED method, we consider a segment of human DNA composed by two complementary strands with sequences 5′-CTGAGGA-3′ and 3′-GACTCCT-5′, hereafter denoted X and Y, respectively. The system is assumed to be electrically neutral by protonating the nonbridging oxygen atoms of the phosphate groups. The starting structures were taken from ref. 26.

The interstrand interaction energy is computed using a supramolecular approach, defined as the energy difference between the DNA duplex and the two isolated strands at the same geometry. To compare the results obtained with standard LED and CovaLED, we examine different structural models.

In the first, simplified model, the sugar–phosphate backbones are removed and the resulting covalent cuts are saturated with hydrogen atoms following the link-atom procedure.^34^ The system then consists of 14 fragments corresponding to the individual nucleobases (see Figure 2B). Because the fragments interact exclusively through noncovalent interactions, this model allows the application of the standard LED scheme.

**Figure 2:**
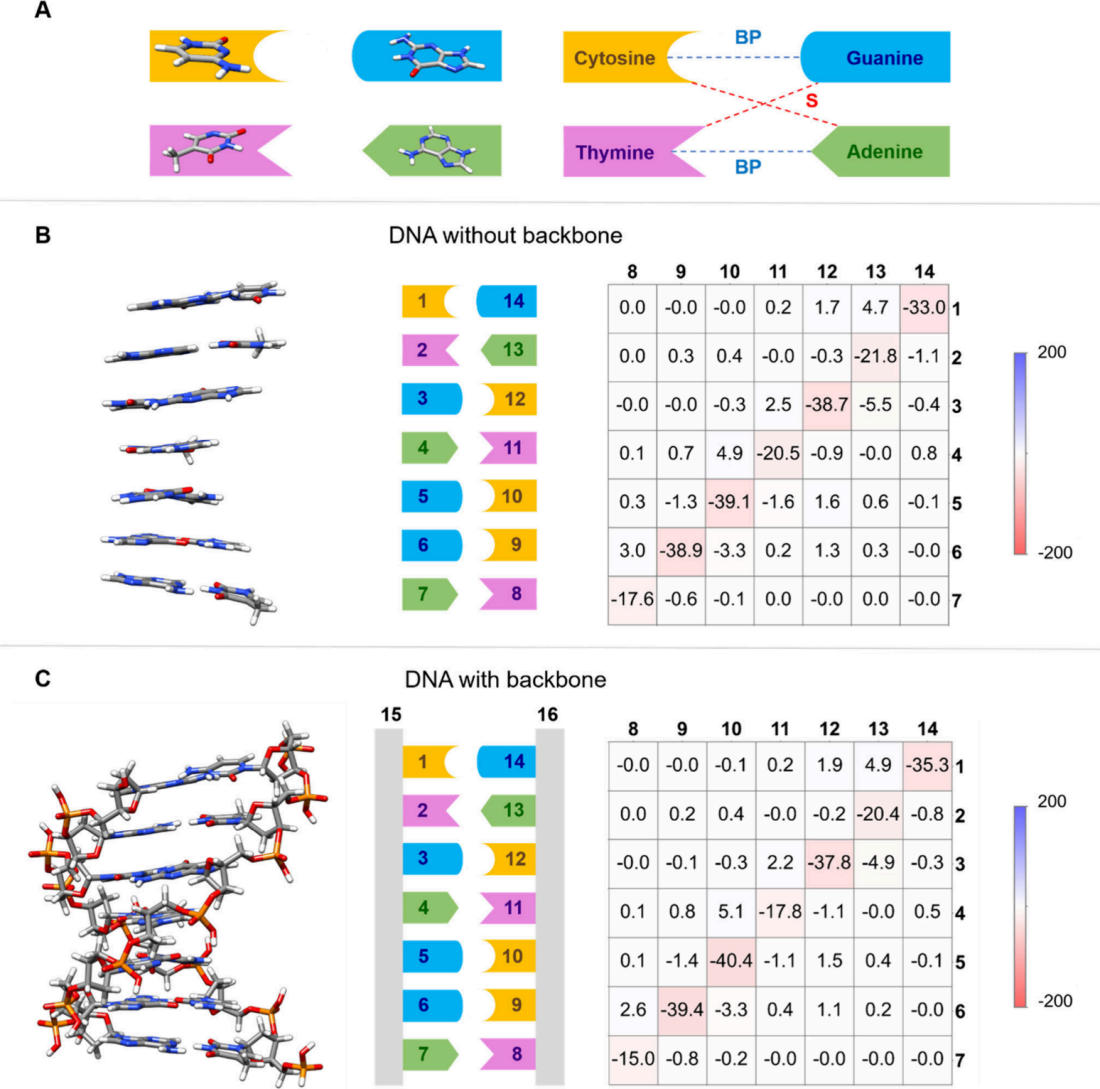
(A) Representation of DNA base pairs and their interactions. The figure highlights hydrogen-bonding interactions within base pairs (BP) and stacking (S) interaction between adjacent base pairs. (B) Structure of the DNA without backbone (206 atoms) with its fragment definition and the corresponding fp-LED map (kcal mol^–1^); (C) structure of the DNA with backbone (comprising 462 atoms), with its fragment definition and corresponding fp-CovaLED map (kcal mol^–1^). All calculations were carried out at the DLPNO–CCSD­(T)/def2-TZVP level. Note that here is reported only the section of the fp-LED maps containing the interstrand base-pair and stacking interaction energy terms between the nucleobases. The full fp-LED maps are reported in the Supporting Information (Figure S1).

For this model, the total interaction energy amounts to −175.3 kcal mol^–1^. The standard fp-LED map (Figure 2B) shows that the dominant contributions arise from hydrogen bonding between complementary nucleobases, with G–C pairs interacting more strongly than A–T pairs due to the additional hydrogen bond. Interstrand stacking interactions also contribute significantly to duplex stabilization. Owing to the right-handed helical structure of B-DNA, the X­(n+1)–Y­(n) base pair is spatially closer than the X­(n)–Y­(n+1) pair, leading to stronger attractive stacking interactions for the former. This trend is consistent with previous HFLD/LED^35,36^ analyses.^26^

In the second model, the sugar–phosphate backbones are retained, yielding a system partitioned into 16 fragments: 14 nucleobases and two backbone moieties (Figure 2C). In this case, nucleobases are covalently connected to the backbone, and the standard LED treatment would lead to an artificial ionic description of the shared orbitals. The CovaLED scheme instead enables a balanced treatment of these covalent connections. The supramolecular interaction energy of the full duplex is –181.0 kcal mol^–1^. Aside from small quantitative differences, the fp-LED map obtained with CovaLED (Figure 2C) is fully consistent to that of the simplified model, both in terms of base-pair hydrogen bonding and interstrand stacking interactions. This agreement indicates that the link-atom protonation used in the simplified model does not significantly distort the underlying interaction pattern, while the CovaLED treatment of covalent connections does not introduce spurious artifacts.

Quantitatively, the sums of the diagonal and off-diagonal elements in panels 2B (−209.5 and 7.83 kcal mol^–1^) and 2C (−206.1 and 7.65 kcal mol^–1^) are essentially identical, confirming that base–base interactions are largely unaffected. The difference in total interaction energy (−175.3 vs –181.0 kcal mol^–1^) instead arises from additional interactions involving the backbone, which are present only in the full system and can be explicitly quantified within the LED framework (see Figure S1). The consistency between the two schemes therefore reinforces the reliability of the decomposition and supports the robustness of the physical interpretation derived from these calculations.

### RNA-Ligand Interaction: Guanine Riboswitches

Riboswitches^29–31^ are noncoding mRNA domains, capable of binding to small target molecules. When the metabolite binds, the RNA undergoes a conformational change in a region distant from the binding site (expression platform), which is responsible for regulating gene expression.

The CovaLED method was employed to investigate the interactions between a model RNA and two ligands with experimentally validated binding affinities:^30^ the guanine (6UBU) and the O6-methylguanine (3F06). The three-dimensional structure of the model system is shown in Figure 3, which includes a close-up of the binding region highlighting the structures of the two ligands.

**Figure 3:**
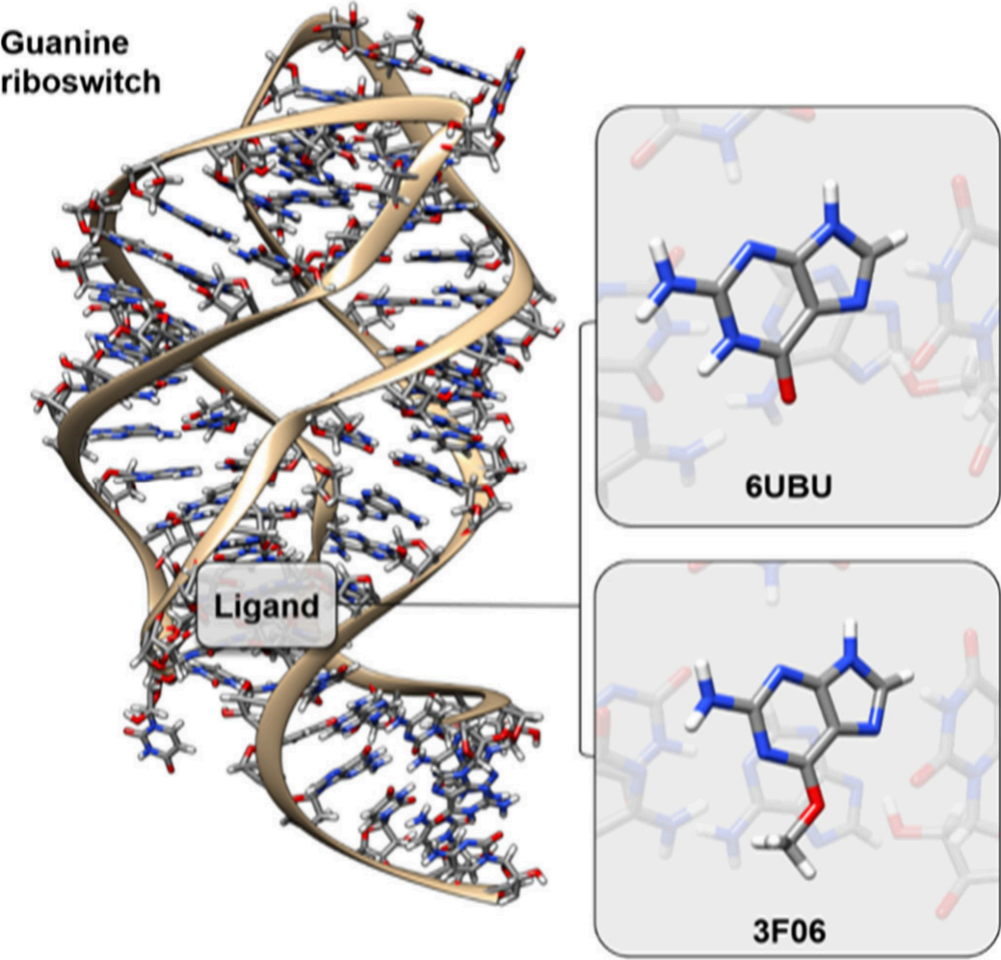
Three-dimensional structure of the RNA molecule (2211 atoms), highlighting the binding region, along with the two possible ligands. The upper right structure shows the natural ligand (6UBU), the lower right structure shows a modified form of the natural ligand bearing a methoxy substitution at position O6 (3F06).

Experimentally, the RNA-guanine complex exhibits a dissociation constant (K_d_) of 0.004 μ M, while O6-methylguanine binds with a significantly weaker affinity (K_d_ = 23 μ M).^30,32^ Using a supramolecular approach, we reproduced this trend computationally through full quantum-mechanical calculations of RNA–ligand binding energies, employing a multilayer DLPNO–CCSD­(T)/HF scheme, as described in the Computational Details. The CovaLED scheme was then used to analyze the complex pattern of noncovalent interactions between the RNA and both ligands within the fp-LED framework.

Figure 4A–B illustrates the fragmentation of the two ligands and of the RNA binding pocket. Each ligand is divided into three fragments (1–3), while fragments 4–9 correspond to the RNA. Fragments 4–8 cover the RNA region directly adjacent to the ligand and primarily responsible for the interaction, whereas fragment 9 encompasses the remainder of the RNA molecule.

**Figure 4:**
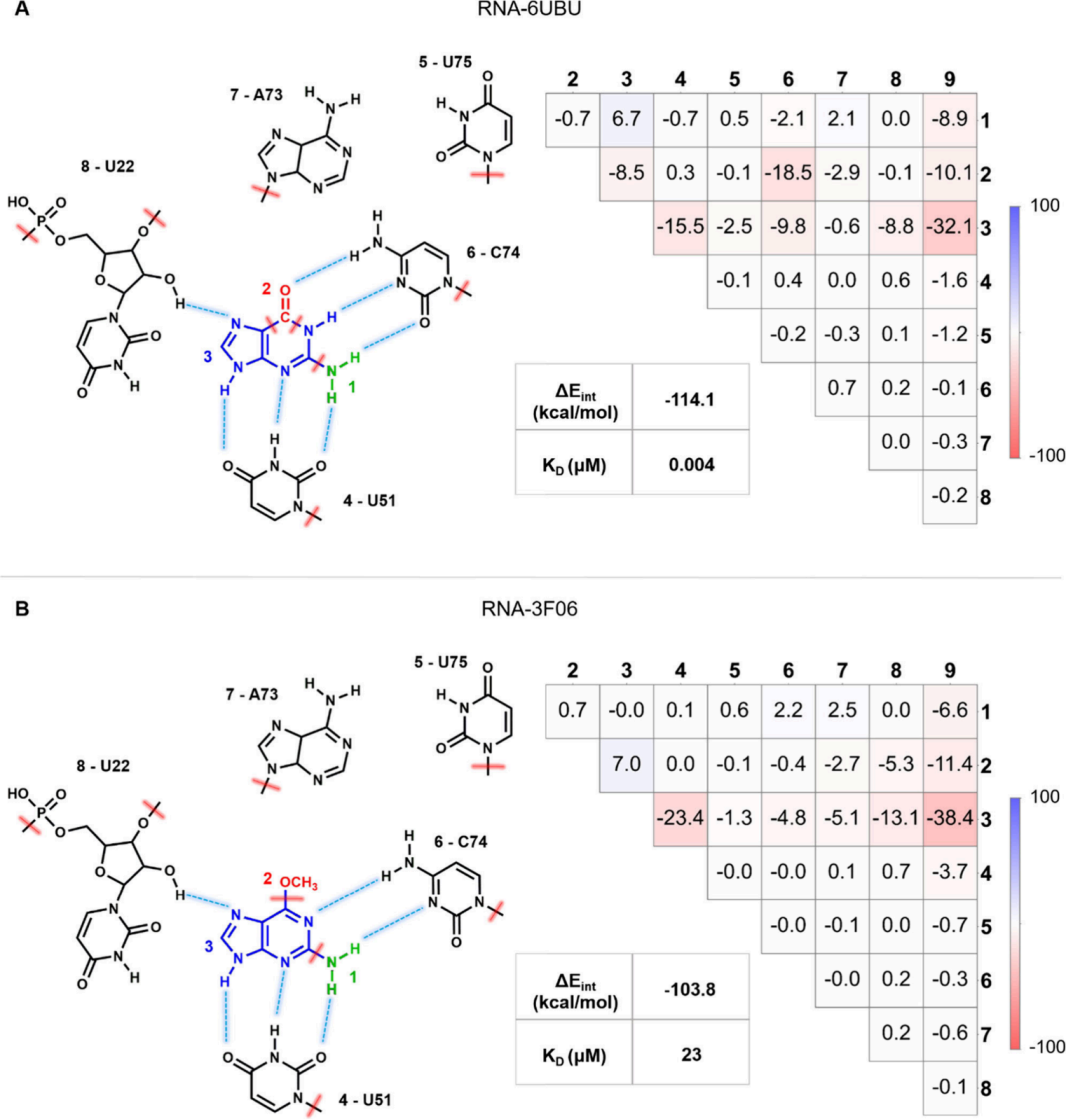
Fragment definition of the ligand and the RNA binding pocket in the (A) RNA-6UBU and (B) RNA-3F06 complexes and corresponding fp-CovaLED maps (kcal mol^–1^). In both systems, the ligand and the binding site are treated at the DLPNO–CCSD­(T)/def2-TZVP­(-f) level. Fragment 9 (not shown) represents the remaining part of the RNA molecule and is treated at HF/MINIX level of theory. The red bar is used to indicate which covalent bonds are treated within the CovaLED scheme.

The total interaction energies amount to – 114.1 kcal mol^–1^ and – 103.8 kcal mol^–1^ for the RNA–6UBU and RNA–3F06 complexes, respectively. Their decomposition into ligand–base interactions is illustrated in the fp-LED maps shown at the right side of Figure 4. Interactions among fragments 1–3 (within the ligand) and among fragments 4–9 (within the RNA) are already present in the isolated molecules; the fp-LED map therefore highlights how these interactions are strengthened or weakened upon complex formation. New interactions that emerge upon complex formation are those established between the ligand fragments (1–3) and the RNA fragments (4–9). Of particular interest are the interactions between the ligand and the nearest RNA binding-pocket region, corresponding to fragments 4–8.

For the RNA–6UBU complex, the most significant RNA–ligand contributions involve residue C74 interacting with the ligand carbonyl group (fragment 2), which contributes – 18.5 kcal mol^–1^ to the total interaction energy. C74 also interacts with fragment 3, the largest structural moiety of the ligand. Residue U51 forms two hydrogen bonds with fragment 3, resulting in an interaction energy of – 15.5 kcal mol^–1^, while U22 (fragment 8) contributes – 8.8 kcal mol^–1^ through a hydrogen bond with the same fragment. In contrast, U75 and A73 (fragments 5 and 7) are only weakly involved, as their distance from the ligand prevents hydrogen-bond formation.

In the RNA–3F06 complex, a similar hydrogen-bonding network is formed between the binding-pocket residues and the ligand. However, substitution of the carbonyl group at position O6 with a methoxy group perturbs the interaction pattern, reducing the number of hydrogen bonds formed by the C74 residue from three, as observed for the RNA-6UBU complex, to two. This modification induces a displacement of residue C74 toward the minor groove.^30^ This effect is directly reflected in the interaction energy between C74 and fragment 2, shown in the map in Figure 4B, which amounts to – 0.4 kcal mol^–1^, compared to – 18.5 kcal mol^–1^ in the RNA–6UBU complex. Similarly, all hydrogen bonds between C74 and the ligand are weakened. This substantial reduction in stabilizing interaction energy results in a pronounced binding penalty, consistent with experimental observations that O6-methylguanine binds approximately 3 orders of magnitude more weakly than guanine.

Finally, it is worth noting that the interaction with fragment 9 (‘the rest of the RNA’) constitutes the largest contribution due to its collective nature, highlighting the crucial role of the surrounding environment in stabilizing the complex.

Importantly, the LED framework offers considerable flexibility in the choice of fragmentation, allowing the analysis to be tailored to the specific chemical question. In the present case, partitioning the ligand into three fragments makes it possible to resolve intermolecular interactions at the level of individual functional groups, while at the same time capturing internal reorganization effects (e.g., ligand polarization) within the ligand. This illustrates how a finer fragmentation can provide a more detailed picture of the interaction, highlighting the flexibility of the approach as one of its key strengths.

## Computational Details

All calculations were performed with a development version of ORCA quantum package based on version 6.1.^37^

Geometry optimization of the ethane–Na^+^ model system was carried out at the RI-MP2 level of theory using the RIJK approximation. The aug-cc-pVTZ basis set was employed.^38^ The auxiliary basis sets were constructing by using the autoaux module of ORCA.^39^ Geometry optimization was performed under C_2 V_ symmetry constraint, which keeps the sodium cation equidistant from the two carbon atoms and positions it such that the axis connecting Na^+^ to the midpoint of the C–C bond is perpendicular to the C–C bond axis. Vibrational frequencies were computed at the same level of theory used in the geometry optimizations. The optimized structure was confirmed as true local minima by the absence of imaginary frequencies. Single point energy calculations were performed at DLPNO–CCSD­(T) level, using aug-cc-pCVnZ (n = T, Q) basis sets and the autoaux module. TightPNO settings were employed. The RIJK approximation was used for the reference calculations. To approach the complete basis set (CBS) and complete PNO space (CPS), CBS (aug-cc-pCVTZ/aug-cc-pCVQZ) and CPS^40,41^ (10^–6^/10^–7^) extrapolations were carried out. Standard frozencore settings were used in all correlated calculations.^42^

For the DNA model systems, the geometry optimization was carried out at GFN2-xTB level.^43^ Energy calculations on both the duplex and the strands were performed with DLPNO–CCSD­(T) method, by using def2-TZVP^44^ basis set and its auxiliary counterparts,^45,46^ and Foster-Boys (FB) localization scheme. The RIJCOSX approximation was used for the calculation of the two-electron integrals.^47^

For the guanine riboswitches, the geometry was optimized using the ONIOM framework at the r2SCAN-3c^48^/GFN2-xTB^43^ level, including implicit solvation via DDCOSMO­(H2O). For the energy calculations a multilayer approach was adopted. The ligand, as well as its interactions with the RNA binding pocket, was treated at DLPNO–CCSD­(T)/def2-TZVP­(-f) level of theory, while the remaining RNA and its interactions were computed at HF/MINIX level. RIJCOSX approximation was used for the evaluation of two-electron integrals, along with def2/J^46^ and def2-TZVP/C^45,46^ auxiliary basis sets. Occupied orbitals were localized using the FB localization scheme.^49^

## Conclusions

In this work, we have introduced the CovaLED framework, an extension of the local energy decomposition (LED) scheme that enables a physically consistent treatment of covalent bonds between fragments within the DLPNO–CCSD­(T) formalism. By allowing shared orbitals to be partitioned in a balanced manner between bonded fragments, CovaLED removes the inherent “ionic” bias of conventional fragment assignments and makes it possible to perform chemically meaningful energy decompositions in intact molecular systems without artificial bond cutting, link atoms, or charge separation.

The method was validated across systems of increasing complexity. For a DNA duplex, comparison between a backbone-truncated link-atom model and the fully covalent system treated with CovaLED shows that both approaches yield consistent base-pairing and stacking interaction patterns. This demonstrates, on the one hand, that the link-atom model does not distort qualitatively the overall interaction picture in this case, and on the other hand that the explicit covalent treatment introduced by CovaLED does not introduce spurious artifacts.

Most importantly, CovaLED enables coupled-cluster-level analysis of molecular recognition in large biomolecular systems. Application to the guanine riboswitch reveals how a chemically subtle ligand modification (O6-methylation) propagates through the interaction network of the binding pocket. The method quantitatively reproduces the experimental binding trend and traces the binding penalty to a specific rebalancing of hydrogen bonding, stacking, electrostatics, and dispersion interactions, with the disruption of the C74–ligand hydrogen bond emerging as the dominant factor. This illustrates how high-level wave function theory, when combined with a chemically transparent decomposition, can translate complex many-electron effects into an interpretable physical picture.

CovaLED therefore bridges a long-standing gap between accuracy and interpretability in electronic-structure analysis of biomolecular interactions. By extending LED to covalently connected fragments, it establishes a general framework for dissecting both inter- and intramolecular interactions at the coupled-cluster level in systems that were previously accessible only through approximate fragmentation strategies. Beyond biomolecular recognition, the approach is expected to be broadly applicable to problems where covalent structure and noncovalent interactions are tightly intertwined, including catalysis, materials chemistry, and enzymatic mechanisms.

## Supplementary Material



## Notes

Theoretical background for standard LED and fragment-pairwise LED (fp-LED); optimized geometric coordinates for ethane-Na^+^, DNA systems with and without backbone, and RNA model systems (RNA-6UBU and RNA-3F06) (PDF)
