# Supplementary material for: Local Energy Decomposition of Intramolecular Interactions: The CovaLED Approach and Its Application to Molecular Recognition in Biomolecular Assemblies
Source: ACS Cent Sci. 2026 May 27;12(6):847–55. doi: 10.1021/acscentsci.6c00336 (PMC13306598; doi:10.1021/acscentsci.6c00336)
Supplement: Supplementary file 1 [file oc6c00336_si_001.pdf]

Supporting Information for Publication

Local Energy Decomposition of Intramolecular Interactions: The CovaLED Approach and its Application to Molecular Recognition in Biomolecular Assemblies

Martina Colucci,† Christoph Riplinger,‡ Ahmet Altun,§ Frank Neese,§ and Giovanni Bistoni*,†

†*Department of Chemistry, Biology and Biotechnology, University of Perugia, 06123 Perugia, Italy*

‡*FAccTs GmbH, Rolandstr. 67, 50677 Köln, Germany*

§*Max-Planck-Institut für Kohlenforschung, Kaiser-Wilhelm-Platz 1, 45470 Mülheim an der Ruhr, Germany*

*Email: giovanni.bistoni@unipg.it

TABLE OF CONTENTS

S1 Theoretical background 2

S1.1 Standard LED 2

S1.2 Fragment-pairwise LED (fp-LED) 4

S2 Ethane-Na$^+$: Basis set dependence of the interaction and binding energies 6

S3 Full fp-LED maps for DNA molecule 7

S4 Geometric Coordinates 8

S4.1 Ethane-Na$^+$ 8

S4.2 DNA with backbone 8

S4.3 DNA without backbone 17

S4.4 RNA-6UBU 22

S4.5 RNA-3F06 67

S1 Theoretical background

S1.1 Standard LED

The LED scheme provides a systematic method for interpreting the results obtained from high level electronic structure calculations within the framework of local correlation approaches. The LED approach involves partitioning the system into a series of chemically meaningful fragments, such as molecules or functional groups and enables the decomposition of the total energy computed at DLPNO-CCSD(T) level. In this framework, the localization of the occupied and virtual orbitals allows for the assignment of each particle in the system to a specific fragment. As a result, the DLPNO-CCSD(T) energy can be broken down into contributions associated with individual fragments and their pairwise interactions. A further decomposition of these energy terms provides a clear-cut definition of different energy contributions, including electrostatic, exchange and dispersive terms. The LED scheme proves particularly advantageous when applied to the decomposition of the binding energy, providing an exact decomposition of the DLPNO-CCSD(T) energy into a sum of fragment and fragment-pairwise contributions.

The standard LED scheme is typically applied alongside local, highly correlated electronic structure methods, such as the DLPNO-CCSD(T) method. In this context, the total DLPNO-CCSD(T) energy is expressed as the sum of a reference (Hartree-Fock) energy and a correlation energy contribution.

$$E = E^{HF} + E^{C} \qquad (S1)$$

Where:

$$E^{HF} = \sum_{A<B} \frac{Z_A Z_B}{|R_A - R_B|} - \sum_{i} \langle i | \nabla_i^{2} | i \rangle - 2 \sum_{i,A} \left\langle i \left| \frac{Z_A}{r_i - R_A} \right| i \right\rangle + 4 \sum_{i_x \leq j_x} \frac{1}{1 + \delta_{ij}} (ii|jj)$$
$$-2 \sum_{i \leq j} \frac{1}{1 + \delta_{ij}} (ij|ji) \qquad (S2)$$

$$E^{C} = \sum_{i \leq j} \varepsilon_{ij} \qquad (S3)$$

Since both the reference and correlation energies depend on one or two particles, the DLPNO-CCSD(T) energy can be decomposed into intra- and inter-fragment contributions, where the intra-fragment terms contain all the energy terms associated to particles belonging to the same fragment, while the inter-fragment contributions contain the energy terms belonging to different fragments. In the following equations, the symbols x and y refers to two arbitrary fragments of the system.

Accordingly, the Hartree-Fock intra-fragment (E_x^{HF}) and inter-fragment (E_{xy}^{HF}) contributions can be written as:

$$E_x^{HF} = \sum_{A_x<B_x} \frac{Z_{A_x}Z_{B_x}}{|R_{A_x} - R_{B_x}|} - \sum_{i_x} \langle i_x | \nabla_{i_x}{}^2 | i_x \rangle - 2 \sum_{i_x, A_x} \left\langle i_x \middle| \frac{Z_{A_x}}{r_{i_x} - R_{A_x}} \middle| i_x \right\rangle$$
$$+4 \sum_{i_x \le j_x} \frac{1}{1+\delta_{i_x j_x}} (i_x i_x | j_x j_x) - 2 \sum_{i_x \le j_x} \frac{1}{1+\delta_{i_x j_x}} (i_x j_x | j_x i_x) \quad (S4)$$

$$E_{xy}^{HF} = \sum_{A_x<B_y} \frac{Z_{A_x}Z_{B_y}}{|R_{A_x} - R_{B_y}|} - 2 \sum_{i_x, A_y} \left\langle i_x \middle| \frac{Z_{A_y}}{r_{i_x} - R_{A_y}} \middle| i_x \right\rangle + 2 \sum_{i_y, A_x} \left\langle i_y \middle| \frac{Z_{A_y}}{r_{i_y} - R_{A_x}} \middle| i_y \right\rangle$$
$$+4 \sum_{i_x<j_y} (i_x i_x | j_y j_y) - 2 \sum_{i_x<j_y} (i_x j_y | j_y i_x) \quad (S5)$$

Similar expressions can be written for the intra-fragment (E_x^C) and inter-fragment correlation energy (E_{xy}^C):

$$E_x^C = \sum_{i_x \le j_x} \epsilon_{i_x j_x} \quad (S6)$$

$$E_{xy}^C = \sum_{i_x<j_y} \epsilon_{i_x j_y} \quad (S7)$$

Within the supramolecular approach, the binding energy is expressed as the energy difference between the adduct and its constituent monomers. In the case of a prototype complex XY consisting of two monomers, X and Y, the binding energy is then expressed as:

$$\Delta E_{bind} = E(XY) - E^0(X) - E^0(Y) \quad (S8)$$

Where $E^0(X)$ and $E^0(Y)$ indicate the energy of the isolated monomers in their equilibrium geometry. By partitioning the system in two fragments, corresponding to the monomers X and Y, the LED scheme decomposes the binding energy into intra- and inter-fragment terms as:

$$\Delta E_{bind} = \Delta E_{geo-prep,x} + \Delta E_{geo-prep,y} + \Delta E_{int} \qquad (S9)$$

The geometric preparation terms $\Delta E_{geo-prep,x}$ and $\Delta E_{geo-prep,y}$ denote the energy required to distort the monomers from their isolated geometry to the geometry adopted in the dimer. ΔE_{int} represents the electronic interaction part of the binding energy, given by the sum of the electronic preparation terms of each fragment and the interaction energy between each fragment pair.

$$\Delta E_{int} = \sum_{x} \Delta E^{x}_{el-prep} + \sum_{xy} E^{xy}_{int} \qquad (S10)$$

The LED scheme provides a physically meaningful decomposition of the interaction between each pair of fragments E^{xy}_{int}. Within the DLPNO-CCSD(T) framework, this term can be decomposed into electrostatic and exchange Hartree-Fock terms and dispersive and non-dispersive correlation terms, providing the well-known expression of the interaction energy decomposition:

$$\Delta E_{int} = \sum_{x} \Delta E^{x}_{el-prep} + \sum_{xy} (E^{els}_{xy} + E^{exch}_{xy} + E^{disp}_{xy} + E^{CT}_{xy}) \qquad (S11)$$

S1.2 Fragment-pairwise LED (fp-LED)

In systems consisting of only two fragments, the sum of the electronic preparation terms is demonstrated to be proportional to the interaction energy between them. On the other hand, when the system is decomposed into more than two fragments, each electronic preparation term is affected by the presence of the other N-1 fragments. This makes the electronic preparation the only term in the energy decomposition that is not pairwise additive.

The fragment-pairwise LED (fp-LED) was developed with the aim of exactly decompose the binding energy in terms of fragment-pairwise interaction energy. fp-LED is based on the definition of

distributed electronic preparation, which describes, for each pair of fragments, the extent to which their interaction changes their electronic structure. Given the fragment pair xy, it is defined as:

$$\Delta E_{el-prep}^{(x,y)} = \omega_x^{(x,y)} \Delta E_{el-prep,x} + \omega_y^{(x,y)} \Delta E_{el-prep,y} \qquad (S12)$$

Where the ω terms represent the contribution of each fragment to the electronic preparation of the other:

$$\omega_x^{(x,y)} = \frac{|E_{int}^{xy}|}{\sum_{\substack{k=1 \\ k\neq x}} |E_{int}^{xk}|} \qquad \omega_y^{(x,y)} = \frac{|E_{int}^{xy}|}{\sum_{\substack{k=1 \\ k\neq y}} |E_{int}^{ky}|} \qquad (S13)$$

Note that absolute values were used for the E_{int}^{xy} terms in the calculation of $the\ \omega\ terms.$ Since $\sum_x \Delta E_{el-prep,x} = \sum_x \Delta E^{(x,y)}{}_{el-prep}$, the interaction energy can be expressed as:

$$\Delta E_{int} = \sum_{x>y} \left[\Delta E_{el-prep}^{(x,y)} + E_{int}^{xy}\right] = \sum_{x>y} \Delta E_{int,xy} \qquad (S14)$$

The latter equation provides an exact decomposition of the interaction energy into additive fragment-pairwise contributions. This decomposition enables to accurately quantify the interaction energy between each fragment pair and to define the contribution of each fragment pair to the total binding energy.

S2 Ethane-Na^+: Basis set dependence of the interaction and binding energies

Table S1 Basis set and TCutPNO threshold dependence of the fragment-pairwise interaction terms and binding energies, computed using both the standard LED and CovaLED approaches. For each basis set (aug-cc-pCVnZ (n=D, T, Q)), the calculations were performed using two values of TCutPNO threshold: $10^{-6}/10^{-7}$. The values obtained from the CBS(3/4)/CPS($10^{-6}/10^{-7}$), representing the most accurate estimates, are those reported in the main manuscript.

Basis set	T_{CutPNO}	Standard LED (kcal mol^{-1})				CovaLED (kcal mol^{-1})			
		$E_{2\text{-}1}$	$E_{3\text{-}1}$	$E_{3\text{-}2}$	ΔE_{int}	$E_{2\text{-}1}$	$E_{3\text{-}1}$	$E_{3\text{-}2}$	ΔE_{int}
aug-cc-pCVDZ	10^{-6}	12.6	137.3	-155.5	-5.6	21.3	-13.4	-13.4	-5.6
	10^{-7}	12.6	137.2	-155.5	-5.7	21.2	-13.5	-13.5	-5.7
aug-cc-pCVTZ	10^{-6}	11.6	136.8	-155.5	-7.0	19.4	-13.2	-13.2	-7.0
	10^{-7}	11.7	136.8	-155.5	-7.0	19.4	-13.2	-13.3	-7.1
aug-cc-pCVQZ	10^{-6}	11.7	136.7	-155.6	-7.2	19.4	-13.3	-13.3	-7.2
	10^{-7}	11.7	136.7	-155.6	-7.2	19.3	-13.3	-13.3	-7.3
CBS(3/4)/CPS($10^{-6}/10^{-7}$) extrapolation		11.7	136.7	-155.7	-7.4	19.3	-13.3	-13.3	-7.4

S3 Full fp-LED maps for DNA molecule

DNA without backbone

	1	2	3	4	5	6	7	8	9	10	11	12	13	14
1		-0.1	0.6	-0.0	0.3	0.2	0.0	0.0	-0.0	-0.0	0.2	1.7	4.7	-33.0
2			1.6	-0.0	-0.0	-0.0	-0.0	0.0	0.3	0.4	-0.0	-0.3	-21.8	-1.1
3				1.3	0.8	-0.0	0.1	-0.0	-0.0	-0.3	2.5	-38.7	-5.5	-0.4
4					0.1	0.2	-0.0	0.1	0.7	4.9	-20.5	-0.9	-0.0	0.8
5						7.9	0.3	0.3	-1.3	-39.1	-1.6	1.6	0.6	-0.1
6							1.7	3.0	-38.9	-3.3	0.2	1.3	0.3	-0.0
7								-17.6	-0.6	-0.1	0.0	-0.0	0.0	-0.0
8									-0.1	-0.0	0.0	0.0	0.0	0.0
9										4.7	-0.0	-0.0	-0.0	0.2
10											0.3	-0.0	0.1	0.3
11												-0.1	-0.0	-0.0
12													5.3	0.4
13														0.3
14														

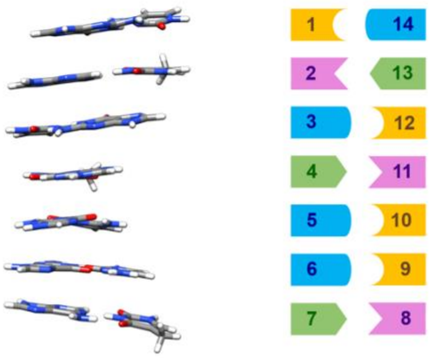

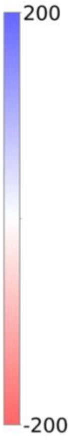

DNA with backbone

	1	2	3	4	5	6	7	8	9	10	11	12	13	14	15	16
1		-0.1	0.6	-0.0	0.3	0.2	0.1	-0.0	-0.0	-0.1	0.2	1.9	4.9	-35.3	0.1	0.1
2			2.3	-0.0	-0.0	-0.0	0.1	0.0	0.2	0.4	-0.0	-0.2	-20.4	-0.8	0.8	-0.7
3				1.0	0.8	-0.0	0.2	-0.0	-0.1	-0.3	2.2	-37.8	-4.9	-0.3	0.8	-5.0
4					-0.0	0.1	0.0	0.1	0.8	5.1	-17.8	-1.1	-0.0	0.5	0.0	0.3
5						7.6	0.4	0.1	-1.4	-40.4	-1.1	1.5	0.4	-0.1	0.4	-4.5
6							1.3	2.6	-39.4	-3.3	0.4	1.1	0.2	-0.0	0.7	-6.2
7								-15.0	-0.8	-0.2	-0.0	-0.0	-0.0	-0.0	0.5	0.5
8									-0.1	-0.0	0.1	0.0	0.0	-0.0	-0.3	2.5
9										3.9	-0.0	-0.0	-0.0	0.2	1.2	1.2
10											-0.0	0.0	0.1	0.3	1.2	0.8
11												-0.0	-0.0	-0.0	-1.2	2.4
12													4.6	0.4	0.4	0.9
13														0.3	0.1	0.9
14															-6.1	1.5
15																-0.3
16																

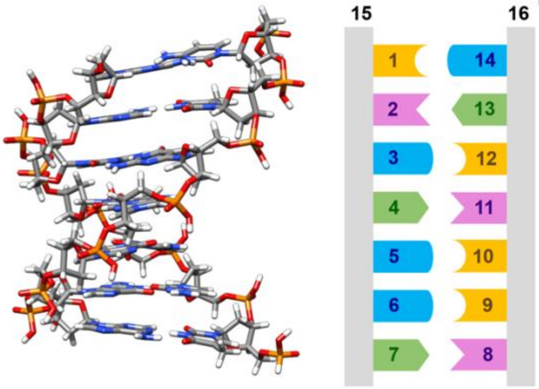

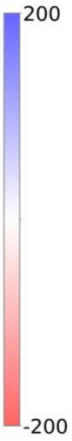

Figure S1. Full fp-LED and fp-CovaLED maps of the DNA molecule without (A) and with (B) backbone, together with the respective fragmentation scheme. Both the maps contain all pairwise interaction terms between nucleobase fragments on the same strand and between the two strands.

S4 Geometric Coordinates

S4.1 Ethane-Na^+

```
9
C       -0.0000000109400    -0.7710800371002    -0.9317150852859
H        0.8950151769228    -1.2092195571277    -0.4876881236594
H        0.0000000011316    -1.1416983531971    -1.9513075547254
H       -0.8950151753572    -1.2092195925737    -0.4876881127800
C       -0.0000000082130     0.7710799963908    -0.9317150760120
H        0.0000000136863     1.1416983600090    -1.9513075227472
H       -0.8950151672029     1.2092195861587    -0.4876881119985
H        0.8950151668812     1.2092195665225    -0.4876881099180
Na       0.0000000030911     0.0000000309177     1.2302416672962
```

S4.2 DNA with backbone

```
   462

 C          2.0323855221220     34.7040745218750     -7.1195171304970
 N          2.0767194006480     34.4991763232710     -8.5458829160190
 C         -0.6649010395700     30.9343499215810     -9.1510786091640
 N          0.2742730220170     30.7256380398680    -10.2134832494860
 C         -1.3762140085260     26.9570457624710    -12.3937150538010
 N          0.0208236556380     26.8157620704850    -12.6718980999670
 C          0.4376836323400     22.8606604950940    -14.8423191858590
 N          1.7858415670690     22.9354840293870    -14.3505080215710
 C          3.9232904157180     19.1996497004460    -16.4269251678120
 N          4.6690401330230     19.3519200916910    -15.2159423474220
 C          7.8983678552230     16.1855615268340    -15.2515662662130
 N          7.8323639940370     16.5257297918000    -13.8451411948430
 C         11.0239116122000     13.5026678889540    -12.1657438779860
 N         10.1284547252190     13.7233906399470    -11.0533381209670
 C          0.5354702397660     13.5878168317050    -12.7651479492480
 N          1.3261625758860     13.9055676409770    -11.6104266759300
 C         -0.9069800132150     17.5476160394350    -10.2326208834280
 N          0.3713817539090     17.8598605989280     -9.6470124003080
 C         -0.1284344681530     21.8792682482530     -7.1020592392120
 N          1.2638990125740     21.9010435582100     -7.4711305066420
 C          2.5937529841670     25.9311261806130     -5.3403161752860
 N          3.5916733579590     25.7441357412810     -6.3740745745960
 C          6.1288123975250     29.7001060088380     -5.6837620294620
 N          6.3273745024740     29.3516791985590     -7.0725539289230
 C          9.5852155892370     32.8143895111260     -8.2191094726820
 N          8.9221371432440     32.4060612983770     -9.4334862992150
 C         10.8175138735640     35.3211097877830    -12.2689782773730
 N          9.5394224845050     35.0289935713420    -12.8943888828120
 P         15.1682604121740     13.2903577976850     -9.5093452820090
```

O	16.3292187377280	13.3250892216970	-10.4085824952450
O	14.7032609064670	14.6855197497190	-8.9440921440040
O	13.8158238321680	12.7779226813150	-10.2049437965220
C	13.9023212200700	11.8492563182270	-11.2726419132120
H	14.9376171269120	11.7759805052030	-11.6170815658870
H	13.5493145099820	10.8766099496040	-10.9210094843150
C	13.0067816707890	12.2978576785220	-12.4339568917350
H	13.1648575041390	11.6094719449990	-13.2730134912330
O	11.6422882059090	12.2360281790250	-12.0483520181930
H	10.3871193538000	13.5255986665710	-13.0620373158590
C	10.5852459392220	13.7014004924310	-9.7756599175130
H	11.6313283535830	13.4636052712210	-9.6496708085890
C	9.7592579866140	13.9569315286590	-8.7381866455500
H	10.1127243134880	13.9526840371260	-7.7232230230210
C	8.3804099570520	14.2128532984220	-9.0314872015510
N	7.5136622683270	14.4935287071860	-8.0759760966070
H	7.8278837016490	14.6062654247820	-7.1269983984570
H	6.5415748064420	14.7044403411470	-8.3172250896720
N	7.9366104751520	14.1318717523110	-10.2866048723300
C	8.7557995345640	13.9013658892060	-11.3119749146740
O	8.3576595922930	13.8545816754820	-12.4759145318870
C	13.2840044801170	13.7424521704660	-12.8940667236280
H	14.2727711612020	14.0822227484560	-12.5753171512240
C	12.1566464238470	14.5328415538510	-12.2497565066390
H	12.4593399865290	14.8444693922900	-11.2501983538480
H	11.8742067826310	15.3978609814350	-12.8385975875040
O	13.2680847012030	13.7509685397220	-14.3224600881850
H	15.3437435241573	15.3862239175290	-9.1146747965320
H	15.2981474423030	12.4975869058610	-8.3958405403730
P	12.8648484666570	14.9606733275680	-15.2648755447740
O	13.3944323474260	14.7677297275510	-16.6165627020430
O	13.2830590037170	16.2731070489580	-14.5019561633450
O	11.2550659249110	15.0737178548000	-15.2276633623530
C	10.5542675781660	14.1340133384490	-16.0290180150560
H	11.1707185499600	13.8692706414950	-16.8921311968110
H	10.3448608036800	13.2392407329220	-15.4368192288080
C	9.2163947416350	14.7129148527730	-16.4984770218770
H	8.7807672019210	14.0043669066950	-17.2144590350090
O	8.3271583856480	14.8476909082790	-15.4044264573060
H	6.8629924560780	16.2760648104460	-15.6156973396650
C	8.9641451370250	16.7044912740180	-13.0976116225560
H	9.8915461151280	16.5510060812210	-13.6289243083220
C	8.9483021768090	17.0299031583220	-11.7910213974120
C	10.1762811639020	17.3079192011560	-10.9836575767220
H	11.0563230302900	17.3443245041020	-11.6157473211950
H	10.0458894895530	18.2613738863410	-10.4761639777600
H	10.3020061888300	16.5479129260740	-10.2146189031830
C	7.6599120533690	17.1426884384760	-11.1275873409630
O	7.5085398202580	17.4173607172750	-9.9434782858290

N	6.5773605678720	16.8818076439660	-11.9267321035780
H	5.6231154239180	17.0234644929670	-11.5023467358010
C	6.5843822898420	16.5754093459920	-13.2500593198180
O	5.5547590841010	16.3546873322050	-13.8672739254550
C	9.3353838056890	16.0976261331850	-17.1633799296610
H	10.3597528371370	16.3028835892010	-17.4819874120170
C	8.8595371711730	17.0506328707330	-16.0774814392520
H	9.7093344543100	17.3706597287540	-15.4792131668530
H	8.3690045181700	17.9219197283950	-16.4965797860150
O	8.5139498699810	16.0823851448860	-18.3307065713650
H	13.6323047095350	16.9676231486830	-15.0713185331640
P	7.8577811892170	17.3356396113190	-19.0490108937030
O	7.5827763697370	17.0522136944250	-20.4591335123950
O	8.7967451877460	18.5579977416480	-18.7282823829900
O	6.5258939224380	17.7146796051840	-18.2174488915550
C	5.3685266552970	16.9546214677330	-18.5398611297970
H	5.4038405173020	16.6924420937530	-19.6006685007390
H	5.3587649150300	16.0427889889120	-17.9365232331360
C	4.0821793112760	17.7310583047480	-18.2339042037220
H	3.2453669322730	17.1543178153710	-18.6459737511260
O	3.8846380100900	17.8449634011590	-16.8338041659240
H	2.9075002026970	19.5261841933440	-16.1657079317070
C	6.0124316651990	19.2308631428990	-14.9868416701410
H	6.6961947479870	18.9627623129750	-15.7648102444760
N	6.3544421681610	19.4668994272970	-13.7584056910420
C	5.1759959838400	19.7592682210460	-13.1100575031810
C	4.8926325808430	20.1720129997420	-11.7799327853450
O	5.6924260040020	20.3573279125690	-10.8622339506920
N	3.5377773362320	20.4061894665150	-11.5808882088340
H	3.2646947154260	20.7534550631940	-10.6360453114940
C	2.5667892456560	20.2561330248670	-12.5310035458510
N	1.3058828492580	20.4983713125680	-12.1782418445400
H	1.0604256518460	20.7594345588630	-11.2233727732350
H	0.6039213700730	20.4530912476440	-12.8962053978150
N	2.8208807272460	19.9018146321890	-13.7750343437190
C	4.1180749144420	19.6742084508960	-14.0140707596180
C	4.0564576406070	19.1642510961720	-18.7992691313170
H	4.7137828840320	19.2692242990770	-19.6648754687330
C	4.5004235612080	19.9913365452860	-17.6061342885490
H	5.5880772246410	19.9874998082230	-17.5536677632180
H	4.1347787811960	21.0119405463000	-17.6389581979420
O	2.7265090197490	19.4376513163640	-19.2422373934400
H	8.8760311484090	19.1939016832280	-19.4484219223810
P	1.8751291884180	20.7670062674710	-19.0814203071610
O	0.6915675290710	20.7201521943240	-19.9411591846830
O	2.8717443871260	21.9688098903480	-19.3034580687510
O	1.5231582593250	20.9491776649040	-17.5190080054350
C	0.3662943569690	20.2864189796000	-17.0269804447780
H	-0.2023132313460	19.8914471569150	-17.8726633202720

H	0.6794394393660	19.4711517346780	-16.3689549647760
C	-0.5333282665170	21.2321510572340	-16.2179356428640
H	-1.4907139295340	20.7226694327590	-16.0615678208790
O	0.0009463466850	21.5177094252450	-14.9346280596090
H	-0.1690735477780	23.3731627489940	-14.0804208016020
C	-0.7598098297140	22.5874059729470	-16.9080329468690
H	-0.6171872370240	22.5127039364050	-17.9889558256610
C	0.2742921634590	23.4790299471360	-16.2382331215240
H	1.2147079299100	23.4131315224830	-16.7821563830170
H	-0.0455031515420	24.5141742818170	-16.1940213792710
O	-2.1122738129530	22.9740795163620	-16.6778196010300
C	2.9632353800210	22.6335077152380	-14.9779024248280
H	2.9915531996430	22.2418276654270	-15.9743675982260
N	4.0180341236570	22.8494473067690	-14.2526457855320
C	3.5350749579370	23.3109529628700	-13.0521438068320
C	4.1457880522300	23.7612815228970	-11.8622595853630
N	5.4584974055980	23.8081856979620	-11.6608985283220
H	6.0641138082450	23.5940028229820	-12.4338265147530
H	5.8261882798420	24.2716709149840	-10.8287582215570
N	3.3191428295970	24.1426670776500	-10.8698599649760
C	2.0033876571760	24.1105713103850	-11.0368443008210
H	1.4183360944640	24.4360993715370	-10.1835075506050
N	1.3307554037500	23.7415750624950	-12.1038179054040
C	2.1361907774280	23.3520312464570	-13.0987798713670
H	2.5673319144190	22.6183697239810	-19.9470774038920
P	-2.6902286578980	24.4467379001050	-16.5476933478400
O	-4.1337339819680	24.4620411430780	-16.7907709788810
O	-1.7989660588350	25.3616942385420	-17.4692770596120
O	-2.2935121819480	24.9848753740190	-15.0800243710070
C	-3.1414120132080	24.5584198357330	-14.0209790228320
H	-4.1418063154810	24.3746505932490	-14.4222933915230
H	-2.7412021471330	23.6358357446950	-13.5916187925850
C	-3.2150449376240	25.6076915634320	-12.9046385014520
H	-3.9837557791270	25.2752345341260	-12.1965141694880
O	-1.9883935448360	25.6939607109420	-12.2009220244560
H	-1.4050187282430	27.5105875287320	-11.4451820243620
C	0.6780297727520	26.3714754359480	-13.7886651122490
H	0.1554574702910	26.0506473737480	-14.6663474368640
N	1.9696741367080	26.3837912542640	-13.6714415795270
C	2.2105243124750	26.8507734579360	-12.3996537966500
C	3.4093649009050	27.1088783726740	-11.6792248528180
O	4.5727716993590	26.9464279461200	-12.0531831400680
N	3.1586020526590	27.6004125606290	-10.4045243591040
H	3.9988716303670	27.8756551447340	-9.8512829822660
C	1.9170986907020	27.8122154824570	-9.8716588245400
N	1.8373541684640	28.2880637774210	-8.6315246459180
H	2.6700454422160	28.4584882540180	-8.0652815969480
H	0.9165046547850	28.4655170579220	-8.2695818070170
N	0.7998947381230	27.5782553266600	-10.5306942222670

C	0.9950000399960	27.1102283459860	-11.7676488788870
C	-3.5394994990990	27.0306895307590	-13.3983440301520
H	-4.0750714430130	27.0122213559890	-14.3503574699500
C	-2.1625927260690	27.6638649692560	-13.5034579353900
H	-1.7298752886090	27.4195800418020	-14.4723416380780
H	-2.1844900473340	28.7405115129660	-13.3719345871960
O	-4.4097488070950	27.6433304604770	-12.4479943798810
H	-2.3025725494690	25.9971243062180	-17.9911471680910
P	-4.3397267742390	29.0772615783400	-11.7711910259970
O	-5.5750997267850	29.3448497223010	-11.0332591791730
O	-3.9588964936710	30.1040918585200	-12.9076789846530
O	-3.0181599436210	29.1415062968070	-10.8574263302070
C	-3.1137529329750	28.6582432801290	-9.5235956570770
H	-4.1661810238620	28.4908227029680	-9.2806391352460
H	-2.5558740827740	27.7205876853580	-9.4501195883130
C	-2.5153524665630	29.6517309997470	-8.5163674066200
H	-2.7744706205370	29.2952569140160	-7.5129446603780
O	-1.1017337966610	29.7069262193530	-8.5900097097760
H	-0.0846093199360	31.4694181128100	-8.3873694441740
C	0.1034789227300	30.3246782652620	-11.5133943069980
H	-0.8570396767160	30.0719858088250	-11.9134952889300
N	1.2073100852780	30.2782226545680	-12.1912064364680
C	2.1923003626300	30.6500123895880	-11.3032852714490
C	3.5989791255280	30.8102750347880	-11.4388076868440
O	4.2870847276880	30.6412442945960	-12.4472973004980
N	4.1987103547290	31.2287895100620	-10.2560966415230
H	5.2227693820130	31.4208307021950	-10.3104077754650
C	3.5354089736600	31.4728414632910	-9.0832679915250
N	4.2438985475530	31.8546249291260	-8.0235407454790
H	5.2354849864210	32.0903086916600	-8.0997279136690
H	3.7207382754490	32.1219098328750	-7.2061942332180
N	2.2314257605550	31.3305604891590	-8.9519201753650
C	1.6137620885850	30.9219165203670	-10.0643445856410
C	-3.0130612904180	31.0947680023720	-8.7134171771340
H	-3.9971164355230	31.1154779103840	-9.1891499865650
C	-1.9214875999040	31.7004835011840	-9.5806917704960
H	-2.1374989800540	31.4933794123060	-10.6272514220530
H	-1.8252529295900	32.7710848299100	-9.4351245052590
O	-3.1601944397780	31.6980291305590	-7.4308218358200
H	-4.5760397864790	30.8403845034500	-12.9846913799170
P	-2.6463062387680	33.1087926300840	-6.9102218063760
O	-3.1959969727290	33.3821197170550	-5.5814997082750
O	-2.9807625096910	34.1634588706620	-8.0392375261850
O	-1.0443700120130	33.1143786694160	-6.9700336927740
C	-0.3322454118540	32.6114344919150	-5.8420972708540
H	-1.0157983661440	32.5438138511080	-4.9918800531480
H	0.0603911708170	31.6211426084340	-6.0867919510550
C	0.8417760542170	33.5289307393840	-5.4833262095230
H	1.2635978270440	33.1734416669670	-4.5356142572920

O	1.8712340428300	33.4668759901060	-6.4565563811370
H	3.0185779820130	35.1147290528610	-6.8578373173030
C	0.4425336005960	35.0082320084910	-5.3362894027200
H	-0.6419973830980	35.0934613925340	-5.1921285218530
C	0.8850698587980	35.6193555611920	-6.6732554853430
H	0.0689163194320	35.5731447267900	-7.3914631525030
H	1.2207771491770	36.6477076069190	-6.5633136102240
O	1.1325512274390	35.5309437383820	-4.2326484161290
C	1.0845107760090	34.0969721665200	-9.3946449404410
H	0.0769569228360	33.9549157846840	-9.0612527685150
N	1.4755781993950	33.9145773417370	-10.6201111792740
C	2.8235928634850	34.1831166114190	-10.6055908002230
C	3.8384726228190	34.1711196778530	-11.5876861181110
N	3.6407156622100	33.9181934772300	-12.8808333335180
H	2.7642201970430	33.5178439636220	-13.1656712436290
H	4.4425048511700	33.8762620653890	-13.5110977372660
N	5.0749050884360	34.5068215973010	-11.1777105679910
C	5.3053535325070	34.8453788867920	-9.9151568203150
H	6.3285156870460	35.1118230638250	-9.6839197543070
N	4.4406369460740	34.9041719536230	-8.9276290449160
C	3.2091315235690	34.5443280220540	-9.3057061735650
H	-3.5229748704390	34.8962962311150	-7.7275305715410
H	0.8404851337150	36.4360640965550	-4.0627441628820
P	12.9959770610980	35.7204854019190	-16.7402907175350
O	14.4478103656430	35.9449899023370	-16.7357425371060
O	12.5236373658710	34.2544884422810	-17.0579381871920
O	12.3073483247770	35.9619851805950	-15.3081119906110
C	12.8143633577160	36.9894493139230	-14.4732290385090
H	13.8737327819790	37.1483790727610	-14.6938808300170
H	12.2547267683000	37.9111057798940	-14.6531150739500
C	12.6365477734040	36.6023467343400	-12.9997683805300
H	13.1487248527170	37.3541019704990	-12.3881308172980
O	11.2614071060300	36.6089291244950	-12.6444859257050
H	10.6196544182470	35.3069920988720	-11.1869274191050
C	9.4339583825960	34.9429755837380	-14.2590264866560
H	10.3381587557160	35.1605934636820	-14.8066148476690
C	8.2894843907920	34.6278470093970	-14.8963908931500
C	8.1929706249740	34.5175217512950	-16.3833537971490
H	9.1783493096570	34.5653193102030	-16.8355681372880
H	7.7050297438860	33.5825988452080	-16.6498432888730
H	7.5745755210050	35.3235379759160	-16.7749153141000
C	7.0965291524640	34.4020027184300	-14.0971746375770
O	6.0026999039720	34.0660665740700	-14.5339867758850
N	7.2657919312480	34.6058488840990	-12.7461944360150
H	6.4010173646870	34.5302334621920	-12.1410489799560
C	8.4151045896030	34.9343152925800	-12.0930371975520
O	8.4503601068580	35.1011662362190	-10.8875445737300
C	13.1768623030480	35.2011094674620	-12.6615471584380
H	13.9213510884470	34.8685954995390	-13.3880120718560

C	11.9251496672560	34.3363580726880	-12.6688226687020
H	11.7556399057210	33.9613834381680	-13.6772871870430
H	12.0097839403810	33.5059261408290	-11.9772743146710
O	13.8180726103120	35.2874578523550	-11.3880712358420
H	13.2510185054860	33.6427819540700	-17.2198333346050
H	12.2630393798130	36.4923789775630	-17.6108172963730
P	14.0994491128110	34.0842992692090	-10.3944601914530
O	15.2269982455550	34.3777296934310	-9.5072400358460
O	14.1923665486350	32.7984295635360	-11.2972901354980
O	12.7260573028760	33.8138781951920	-9.5858401069290
C	12.4691666996740	34.7338308509780	-8.5336672363280
H	13.3856551798610	34.8906835230260	-7.9592259076690
H	12.1461182903560	35.6871677384480	-8.9609102727430
C	11.3505673566020	34.2061338776750	-7.6344215690250
H	11.1979252465670	34.9347723778510	-6.8271164775120
O	10.1604180499600	34.0916620472240	-8.3939777627020
H	8.8023631040960	32.8525246789160	-7.4470891489700
C	9.6181551097770	32.3087858273560	-10.5884432944540
H	10.6632840290500	32.5706094294210	-10.5238520752060
C	9.0225727925200	31.9058901473320	-11.7315675881560
H	9.5656367655080	31.8086773961540	-12.6541295678810
C	7.6207370128650	31.6125602273120	-11.6756630771130
N	6.9820315222730	31.1375670225260	-12.7296265373590
H	7.4627045720410	31.0049396048950	-13.6031527956970
H	5.9774028365110	30.9395317817460	-12.6732433482940
N	6.9374061262870	31.7936032290450	-10.5460419913850
C	7.5246903111600	32.2212854126070	-9.4268207559050
O	6.9097778206050	32.4007730490870	-8.3792632008820
C	11.6265329496250	32.8222539834650	-7.0043500403370
H	12.6838996426320	32.5562966989340	-7.0604521072870
C	10.7397121853650	31.8879595719500	-7.8109739888650
H	11.2864051960970	31.5502317827170	-8.6906015510110
H	10.4070000084910	31.0353435845210	-7.2289380012710
O	11.2712095345420	32.9091325996750	-5.6244433157530
H	14.7656562293000	32.1058483046580	-10.9502472974810
P	10.7998458173010	31.7165702312510	-4.6901563378660
O	10.9578282910310	32.0625655749110	-3.2766115282790
O	11.5613141536280	30.4442921720270	-5.2216292413310
O	9.2725005962420	31.3671759372380	-5.0851750627510
C	8.2632292996660	32.1394955152590	-4.4474895858410
H	8.6405431235680	32.4913688094330	-3.4841366683700
H	8.0112889325090	32.9944675031310	-5.0804558505530
C	6.9879942324480	31.3156934763690	-4.2438531675690
H	6.3094109772820	31.9085028556520	-3.6172684113680
O	6.3622492716590	31.0775521545870	-5.4928309885820
H	5.0776872027720	29.4582661784310	-5.4676889812000
C	7.5214507306630	29.5663280489050	-7.6731082819800
H	8.2669204158390	30.0559456977220	-7.0644652645280
C	7.7405813259700	29.1777292789120	-8.9486204805530

H	8.6915639591500	29.3146740249280	-9.4306974510110
C	6.6455542190940	28.5830678116950	-9.6579759576550
N	6.7855737616340	28.1447007784800	-10.8942719640240
H	7.6866309558230	28.1620044694660	-11.3403594834930
H	5.9947530183760	27.6917487252590	-11.3646692753910
N	5.4527725507790	28.4674162447930	-9.0740675130330
C	5.2447635209280	28.8346971023520	-7.8098494547740
O	4.1593822007230	28.6943079373080	-7.2500758434030
C	7.2207177468500	29.9434739358640	-3.5769348096200
H	8.1985342412970	29.8955885274240	-3.0931276985820
C	7.0883808207070	28.9675148269770	-4.7350880690080
H	8.0615289471050	28.8380873462170	-5.2067125189290
H	6.6961356113040	28.0083984222170	-4.4171919754410
O	6.2281509003790	29.7808303670290	-2.5646854965630
H	11.8126725002610	29.8097726551160	-4.5410380173390
P	5.6802854767150	28.4062116978750	-1.9927413062550
O	5.0950448116900	28.5825028762060	-0.6624154884880
O	6.8646601183380	27.3790330798650	-2.1394286462250
O	4.6224696938620	27.8294908189170	-3.0699356463720
C	3.3260115756850	28.4087851018420	-3.0190067853860
H	3.0808025133040	28.6446310139890	-1.9802818116180
H	3.3176398708470	29.3249896597060	-3.6151300214930
C	2.2823419420710	27.4554116173770	-3.6057198233830
H	1.2964115490140	27.9153328129280	-3.4582722805670
O	2.5095389035200	27.2941419639720	-4.9948385649450
H	1.6454079234930	25.5978970303230	-5.7884926531890
C	4.9120368872430	25.9997618674510	-6.1196684286960
H	5.1159175325400	26.3830840045160	-5.1308181453090
C	5.8917221268590	25.7961320623850	-7.0221892628960
C	7.3485078166120	25.9756488617480	-6.7314364104020
H	7.5275144529070	26.0168131974910	-5.6628640622680
H	7.8963528366110	25.1457457895280	-7.1719761692740
H	7.7168136569330	26.8860782008260	-7.1998245300530
C	5.5134490572930	25.3513850054700	-8.3546258940470
O	6.3041152546740	25.1191179659260	-9.2596835067550
N	4.1652372784010	25.1780334538420	-8.5462274451680
H	3.8511931373900	24.8195879883490	-9.4928660960340
C	3.1694461154900	25.3739906135730	-7.6400154201170
O	1.9976332251570	25.1856872213930	-7.9113163852730
C	2.2703620366500	26.0470732488760	-2.9734066958450
H	2.8202423101400	26.0253787704560	-2.0304420473980
C	2.9074223039770	25.1713093209170	-4.0421305309890
H	3.9815889089830	25.1252050790880	-3.8728656865800
H	2.4943617989700	24.1684659748320	-4.0418445962590
O	0.9091477680850	25.7233645749180	-2.6868611867790
H	6.8897643469970	26.6968512274320	-1.4587489953670
P	0.2885029115530	24.2697999921050	-2.5607747449130
O	-0.9483510404480	24.2850361168870	-1.7778732071170
O	1.4614996833390	23.3496945914520	-2.0572562269680

O	0.0665020559840	23.7205658569990	-4.0662015774300
C	-1.1145746129210	24.1739080005510	-4.7115408315140
H	-1.9080881410090	24.2842249905080	-3.9679901275010
H	-0.9179805346980	25.1393779576670	-5.1852981089450
C	-1.5450797609420	23.1915979167850	-5.8040002248300
H	-2.5069706153160	23.5371563138280	-6.2040171728650
O	-0.5877180478990	23.1915188572500	-6.8504259920900
H	-0.6564651620700	21.4575695043880	-7.9684808167370
C	2.1887194128340	22.3906671620440	-6.6157225262060
H	1.7981695357350	22.7963034600880	-5.6946833903610
C	3.5037645974100	22.3615338363490	-6.9180725615030
H	4.2528117997800	22.7296257413330	-6.2405887423990
C	3.8738212149240	21.8053109961380	-8.1855427124050
N	5.1430600002280	21.6571213148470	-8.5189696079000
H	5.8694262955550	21.9209005010310	-7.8751252473400
H	5.3898923199320	21.1972301996620	-9.4048834520870
N	2.9385652456250	21.4224596526340	-9.0544570263480
C	1.6403189801430	21.4765651693050	-8.7610481515220
O	0.7604790951130	21.1406225725240	-9.5548101777420
C	-1.6936838624510	21.7323882663100	-5.3234510150050
H	-1.8029520692110	21.6751294011530	-4.2382873353380
C	-0.4183822405820	21.0784891370350	-5.8258340516780
H	0.3755600913380	21.2309586725630	-5.0961006255940
H	-0.5383749749390	20.0184562807820	-6.0192307090840
O	-2.8918955623080	21.2088372299740	-5.8984784274200
H	1.1873125838830	22.6228510195810	-1.4866913804220
P	-3.1651922007400	19.7338050029290	-6.4153070048700
O	-4.5992294814720	19.5141849020620	-6.6077092369590
O	-2.4183882922390	18.7691690512740	-5.4168339060120
O	-2.2981984802150	19.5200518371790	-7.7582332017950
C	-2.8796563773960	19.9448279429080	-8.9826502859990
H	-3.9175928272540	20.2332764570240	-8.7990420211230
H	-2.3111352485430	20.7970614050780	-9.3657351060800
C	-2.8489160205560	18.8420064144360	-10.0520289976210
H	-3.5526618421330	19.1317039276590	-10.8404185853500
O	-1.5799517578660	18.7122650363830	-10.6657766204510
H	-0.6658060007870	16.9458898006580	-11.1218445862150
C	-3.2104178679300	17.4524824346580	-9.5021564257700
H	-3.8100026887360	17.5301685826090	-8.5911485842480
C	-1.8468642668810	16.8207146421570	-9.2545500208140
H	-1.5374134762010	17.0139579246620	-8.2295071511800
H	-1.8631429875350	15.7501318959110	-9.4264311725980
O	-4.0135813194590	16.7928873177890	-10.4759164206590
C	0.6677037448410	18.3590462194330	-8.4094820983000
H	-0.0975906094230	18.6424668179970	-7.7162637736380
N	1.9394723128600	18.4671863949620	-8.1707991563460
C	2.5523591748950	18.0279320955950	-9.3197430049210
C	3.9008953750280	17.8344566954090	-9.6921020916300
N	4.9397118848620	18.0897509205380	-8.9075720727590

H	4.7630386492530	18.3707976410000	-7.9598536724280
H	5.8857184596860	17.8755892788780	-9.2288294069490
N	4.1189363642120	17.3611301869680	-10.9340810221280
C	3.1013507893160	17.0874683174780	-11.7388145233190
H	3.3746908355170	16.7189434869730	-12.7222541407830
N	1.8151237688030	17.1988157426250	-11.4892496970130
C	1.5777436538120	17.6646414507090	-10.2569190023640
H	-2.9580117425510	18.0406059838270	-5.0899607800360
P	-3.9927699489640	15.2559392884670	-10.8691978269210
O	-5.1593090372900	14.9149957533820	-11.6857144431690
O	-3.8180761502100	14.4671009888250	-9.5137099596790
O	-2.5904326622360	14.9644761941570	-11.5951202582070
C	-2.5162389406450	15.2752931491940	-12.9825700426050
H	-3.4977954746260	15.1201691009720	-13.4378275846250
H	-2.2154919051640	16.3195724024690	-13.1013695478180
C	-1.4667940230480	14.3856698636070	-13.6476094286400
H	-1.4981463973110	14.5680229853240	-14.7306948373270
O	-0.1845707153430	14.7367824372080	-13.1571663820110
H	1.2497656381920	13.3046754172950	-13.5529271929430
C	0.8871858535630	14.3797681481770	-10.4032148277390
H	-0.1558724200970	14.4911110234940	-10.1864821223320
N	1.8431829526060	14.6674297303370	-9.5746960438220
C	3.0031725566890	14.3926747368630	-10.2621233497450
C	4.3764613917230	14.5221439583040	-9.9069897328410
O	4.8535059263460	14.9270230322550	-8.8453424065300
N	5.2177686247290	14.0995985689780	-10.9263999382050
H	6.2433193187590	14.1558262928160	-10.7286983605980
C	4.8086153863940	13.6174784700600	-12.1375591622210
N	5.7465158071610	13.1395930885840	-12.9628880200060
H	6.7225570825770	13.4191110797110	-12.8315086135560
H	5.4417622656720	12.9425895826900	-13.9009943455550
N	3.5411106285270	13.5087184796840	-12.4836931218730
C	2.6859243381750	13.9250830925720	-11.5401374615330
C	-1.6776444486080	12.8763249794470	-13.4045340003170
H	-2.6399103894270	12.7084492771290	-12.9048492288270
C	-0.5001052514550	12.4835163580940	-12.5026697089000
H	-0.7967538331080	12.5015492452770	-11.4556569770930
H	-0.1009476136810	11.5047569144180	-12.7568815799720
O	-1.6588775657330	12.2388339517420	-14.6549631018690
H	-4.3136180501800	13.6413024720800	-9.4739569085460
H	-1.8513307635470	11.2971036650210	-14.5429161330010

S4.3 DNA without backbone

206

N	10.128454725219	13.723390639947	-11.053338120967
C	10.585245939222	13.701400492431	-9.775659917513
H	11.631328353583	13.463605271221	-9.649670808589
C	9.759257986614	13.956931528659	-8.73818664555
H	10.112724313488	13.952684037126	-7.723223023021
C	8.380409957052	14.212853298422	-9.031487201551
N	7.513662268327	14.493528707186	-8.075976096607
H	7.827883701649	14.606265424782	-7.126998398457
H	6.541574806442	14.704440341147	-8.317225089672
N	7.936610475152	14.131871752311	-10.28660487233
C	8.755799534564	13.901365889206	-11.311974914674
O	8.357659592293	13.854581675482	-12.475914531887
H	10.766000000000	13.566000000000	-11.846000000000
N	7.832363994037	16.5257297918	-13.845141194843
C	8.964145137025	16.704491274018	-13.097611622556
H	9.891546115128	16.551006081221	-13.628924308322
C	8.948302176809	17.029903158322	-11.791021397412
C	10.176281163902	17.307919201156	-10.983657576722
H	11.05632303029	17.344324504102	-11.615747321195
H	10.045889489553	18.261373886341	-10.47616397776
H	10.30200618883	16.547912926074	-10.214618903183
C	7.659912053369	17.142688438476	-11.127587340963
O	7.508539820258	17.417360717275	-9.943478285829
N	6.577360567872	16.881807643966	-11.926732103578
H	5.623115423918	17.023464492967	-11.502346735801
C	6.584382289842	16.575409345992	-13.250059319818
O	5.554759084101	16.354687332205	-13.867273925455
H	7.879000000000	16.284000000000	-14.845000000000
N	4.669040133023	19.351920091691	-15.215942347422
C	6.012431665199	19.230863142899	-14.986841670141
H	6.696194747987	18.962762312975	-15.764810244476
N	6.354442168161	19.466899427297	-13.758405691042
C	5.17599598384	19.759268221046	-13.110057503181
C	4.892632580843	20.172012999742	-11.779932785345
O	5.692426004002	20.357327912569	-10.862233950692
N	3.537777336232	20.406189466515	-11.580888208834
H	3.264694715426	20.753455063194	-10.636045311494
C	2.566789245656	20.256133024867	-12.531003545851
N	1.305882849258	20.498371312568	-12.17824184454
H	1.060425651846	20.759434558863	-11.223372773235
H	0.603921370073	20.453091247644	-12.896205397815
N	2.820880727246	19.901814632189	-13.775034343719
C	4.118074914442	19.674208450896	-14.014070759618
H	4.132000000000	19.243000000000	-16.088000000000
N	1.785841567069	22.935484029387	-14.350508021571
C	2.963235380021	22.633507715238	-14.977902424828
H	2.991553199643	22.241827665427	-15.974367598226
N	4.018034123657	22.849447306769	-14.252645785532
C	3.535074957937	23.31095296287	-13.052143806832

C	4.14578805223	23.761281522897	-11.862259585363
N	5.458497405598	23.808185697962	-11.660898528322
H	6.064113808245	23.594002822982	-12.433826514753
H	5.826188279842	24.271670914984	-10.828758221557
N	3.319142829597	24.14266707765	-10.869859964976
C	2.003387657176	24.110571310385	-11.036844300821
H	1.418336094464	24.436099371537	-10.183507550605
N	1.33075540375	23.741575062495	-12.103817905404
C	2.136190777428	23.352031246457	-13.098779871367
H	0.809000000000	22.846000000000	-14.66500000000
N	0.020823655638	26.815762070485	-12.671898099967
C	0.678029772752	26.371475435948	-13.788665112249
H	0.155457470291	26.050647373748	-14.666347436864
N	1.969674136708	26.383791254264	-13.671441579527
C	2.210524312475	26.850773457936	-12.39965379665
C	3.409364900905	27.108878372674	-11.679224852818
O	4.572771699359	26.94642794612	-12.053183140068
N	3.158602052659	27.600412560629	-10.404524359104
H	3.998871630367	27.875655144734	-9.851282982266
C	1.917098690702	27.812215482457	-9.87165882454
N	1.837354168464	28.288063777421	-8.631524645918
H	2.670045442216	28.458488254018	-8.065281596948
H	0.916504654785	28.465517057922	-8.269581807017
N	0.799894738123	27.57825532666	-10.530694222267
C	0.995000039996	27.110228345986	-11.767648878887
H	-0.984000000000	26.917000000000	-12.472000000000
N	0.274273022017	30.725638039868	-10.213483249486
C	0.10347892273	30.324678265262	-11.513394306998
H	-0.857039676716	30.071985808825	-11.91349528893
N	1.207310085278	30.278222654568	-12.191206436468
C	2.19230036263	30.650012389588	-11.303285271449
C	3.598979125528	30.810275034788	-11.438807686844
O	4.287084727688	30.641244294596	-12.447297300498
N	4.198710354729	31.228789510062	-10.256096641523
H	5.222769382013	31.420830702195	-10.310407775465
C	3.53540897366	31.472841463291	-9.083267991525
N	4.243898547553	31.854624929126	-8.023540745479
H	5.235484986421	32.09030869166	-8.099727913669
H	3.720738275449	32.121909832875	-7.206194233218
N	2.231425760555	31.330560489159	-8.951920175365
C	1.613762088585	30.921916520367	-10.064344585641
H	-0.40100000000	30.876000000000	-9.450000000000
N	2.076719400648	34.499176323271	-8.545882916019
C	1.084510776009	34.09697216652	-9.394644940441
H	0.076956922836	33.954915784684	-9.061252768515
N	1.475578199395	33.914577341737	-10.620111179274
C	2.823592863485	34.183116611419	-10.605590800223
C	3.838472622819	34.171119677853	-11.587686118111
N	3.64071566221	33.91819347723	-12.880833333518

H	2.764220197043	33.517843963622	-13.165671243629
H	4.44250485117	33.876262065389	-13.511097737266
N	5.074905088436	34.506821597301	-11.177710567991
C	5.305353532507	34.845378886792	-9.915156820315
H	6.328515687046	35.111823063825	-9.683919754307
N	4.440636946074	34.904171953623	-8.927629044916
C	3.209131523569	34.544328022054	-9.305706173565
H	2.045000000000	34.645000000000	-7.527000000000
N	9.539422484505	35.028993571342	-12.894388882812
C	9.433958382596	34.942975583738	-14.259026486656
H	10.338158755716	35.160593463682	-14.806614847669
C	8.289484390792	34.627847009397	-14.89639089315
C	8.192970624974	34.517521751295	-16.383353797149
H	9.178349309657	34.565319310203	-16.835568137288
H	7.705029743886	33.582598845208	-16.649843288873
H	7.574575521005	35.323537975916	-16.7749153141
C	7.096529152464	34.40200271843	-14.097174637577
O	6.002699903972	34.06606657407	-14.533986775885
N	7.265791931248	34.605848884099	-12.746194436015
H	6.401017364687	34.530233462192	-12.141048979956
C	8.415104589603	34.93431529258	-12.093037197552
O	8.450360106858	35.101166236219	-10.88754457373
H	10.44600000000	35.236000000000	-12.45100000000
N	8.922137143244	32.406061298377	-9.433486299215
C	9.618155109777	32.308785827356	-10.588443294454
H	10.66328402905	32.570609429421	-10.523852075206
C	9.02257279252	31.905890147332	-11.731567588156
H	9.565636765508	31.808677396154	-12.654129567881
C	7.620737012865	31.612560227312	-11.675663077113
N	6.982031522273	31.137567022526	-12.729626537359
H	7.462704572041	31.004939604895	-13.603152795697
H	5.977402836511	30.939531781746	-12.673243348294
N	6.937406126287	31.793603229045	-10.546041991385
C	7.52469031116	32.221285412607	-9.426820755905
O	6.909777820605	32.400773049087	-8.379263200882
H	9.396000000000	32.697000000000	-8.566000000000
N	6.327374502474	29.351679198559	-7.072553928923
C	7.521450730663	29.566328048905	-7.67310828198
H	8.266920415839	30.055945697722	-7.064465264528
C	7.74058132597	29.177729278912	-8.948620480553
H	8.69156395915	29.314674024928	-9.430697451011
C	6.645554219094	28.583067811695	-9.657975957655
N	6.785573761634	28.14470077848	-10.894271964024
H	7.686630955823	28.162004469466	-11.340359483493
H	5.994753018376	27.691748725259	-11.364669275391
N	5.452772550779	28.467416244793	-9.074067513033
C	5.244763520928	28.834697102352	-7.809849454774
O	4.159382200723	28.694307937308	-7.250075843403
H	6.18600000000	29.600000000000	-6.08300000000

N	3.591673357959	25.744135741281	-6.374074574596
C	4.912036887243	25.999761867451	-6.119668428696
H	5.11591753254	26.383084004516	-5.130818145309
C	5.891722126859	25.796132062385	-7.022189262896
C	7.348507816612	25.975648861748	-6.731436410402
H	7.527514452907	26.016813197491	-5.662864062268
H	7.896352836611	25.145745789528	-7.171976169274
H	7.716813656933	26.886078200826	-7.199824530053
C	5.513449057293	25.35138500547	-8.354625894047
O	6.304115254674	25.119117965926	-9.259683506755
N	4.165237278401	25.178033453842	-8.546227445168
H	3.85119313739	24.819587988349	-9.492866096034
C	3.16944611549	25.373990613573	-7.640015420117
O	1.997633225157	25.185687221393	-7.911316385273
H	2.883000000000	25.877000000000	-5.639000000000
N	1.263899012574	21.90104355821	-7.471130506642
C	2.188719412834	22.390667162044	-6.615722526206
H	1.798169535735	22.796303460088	-5.694683390361
C	3.50376459741	22.361533836349	-6.918072561503
H	4.25281179978	22.729625741333	-6.240588742399
C	3.873821214924	21.805310996138	-8.185542712405
N	5.143060000228	21.657121314847	-8.5189696079
H	5.869426295555	21.920900501031	-7.87512524734
H	5.389892319932	21.197230199662	-9.404883452087
N	2.938565245625	21.422459652634	-9.054457026348
C	1.640318980143	21.476565169305	-8.761048151522
O	0.760479095113	21.140622572524	-9.554810177742
H	0.269000000000	21.885000000000	-7.207000000000
N	0.371381753909	17.859860598928	-9.647012400308
C	0.667703744841	18.359046219433	-8.4094820983
H	-0.097590609423	18.642466817997	-7.716263773638
N	1.93947231286	18.467186394962	-8.170799156346
C	2.552359174895	18.027932095595	-9.319743004921
C	3.900895375028	17.834456695409	-9.69210209163
N	4.939711884862	18.089750920538	-8.907572072759
H	4.763038649253	18.370797641	-7.959853672428
H	5.885718459686	17.875589278878	-9.228829406949
N	4.118936364212	17.361130186968	-10.934081022128
C	3.101350789316	17.087468317478	-11.738814523319
H	3.374690835517	16.718943486973	-12.722254140783
N	1.815123768803	17.198815742625	-11.489249697013
C	1.577743653812	17.664641450709	-10.256919002364
H	-0.543000000000	17.637000000000	-10.066000000000
N	1.326162575886	13.905567640977	-11.61042667593
C	0.887185853563	14.379768148177	-10.403214827739
H	-0.155872420097	14.491111023494	-10.186482122332
N	1.843182952606	14.667429730337	-9.574696043822
C	3.003172556689	14.392674736863	-10.262123349745
C	4.376461391723	14.522143958304	-9.906989732841

O	4.853505926346	14.927023032255	-8.84534240653
N	5.217768624729	14.099598568978	-10.926399938205
H	6.243319318759	14.155826292816	-10.728698360598
C	4.808615386394	13.61747847006	-12.137559162221
N	5.746515807161	13.139593088584	-12.962888020006
H	6.722557082577	13.419111079711	-12.831508613556
H	5.441762265672	12.94258958269	-13.900994345555
N	3.541110628527	13.508718479684	-12.483693121873
C	2.685924338175	13.925083092572	-11.540137461533
H	0.758000000000	13.678000000000	-12.439000000000

S4.4 RNA-6UBU

2227

O	3.9119999999999900	4.1479999999999900	20.8919999999999000
C	4.6840000000000000	3.6789999999999900	21.9819999999999000
C	4.6189999999999900	2.1480000000000000	22.1600000000000000
O	3.2820000000000000	1.7220000000000000	22.4349999999999000
C	4.9390000000000000	1.2969999999999900	20.9370000000000000
O	6.2999999999999900	1.3930000000000000	20.5090000000000000
C	4.3230000000000000	-0.0550000000000000	21.3210000000000000
O	4.9420000000000000	-0.7930000000000000	22.3619999999999000
C	2.9860000000000000	0.4630000000000000	21.8689999999999000
N	1.8910000000000000	0.6080000000000000	20.8240000000000000
C	1.3710000000000000	1.7380000000000000	20.2970000000000000
N	0.4470000000000000	1.5240000000000000	19.3930000000000000
C	0.3720000000000000	0.1870000000000000	19.3150000000000000
C	-0.4840000000000000	-0.6250000000000000	18.4740000000000000
O	-1.3320000000000000	-0.2620000000000000	17.6600000000000000
N	-0.2110000000000000	-1.9490000000000000	18.7049999999999000
C	0.7110000000000000	-2.4830000000000000	19.5869999999999000
N	0.7900000000000000	-3.8159999999999900	19.6099999999999000
N	1.4610000000000000	-1.7529999999999900	20.3460000000000000
C	1.2529999999999900	-0.4130000000000000	20.1739999999999000
H	4.3540000000000000	3.9070000000000000	20.0749999999999000
H	4.3440000000000000	4.1710000000000000	22.8939999999999000
H	5.7180000000000000	3.9910000000000000	21.8369999999999000
H	5.2549999999999900	1.8590000000000000	22.9989999999999000
H	4.4039999999999900	1.6870000000000000	20.0689999999999000
H	4.1490000000000000	-0.6909999999999990	20.4600000000000000
H	5.7389999999999900	-1.2020000000000000	22.0169999999999000
H	2.5779999999999900	-0.1870000000000000	22.6460000000000000
H	1.7050000000000000	2.7090000000000000	20.6299999999999000
H	-0.7580000000000000	-2.5939999999999900	18.1509999999999000
H	1.4390000000000000	-4.2709999999999900	20.2330000000000000
H	0.2140000000000000	-4.3819999999999900	18.9989999999999000
P	7.5199999999999900	0.4370000000000000	21.0139999999999000

O	7.7260000000000000	0.3880000000000000	22.4879999999999000
O	8.7870000000000000	0.9870000000000000	20.1610000000000000
O	7.1859999999999900	-0.9820000000000000	20.2890000000000000
C	7.5549999999999900	-2.2069999999999900	20.8990000000000000
C	6.9080000000000000	-3.3790000000000000	20.1469999999999000
O	5.5030000000000000	-3.1789999999999900	20.0339999999999000
C	7.2820000000000000	-3.5030000000000000	18.6829999999999000
O	8.6029999999999900	-4.0129999999999900	18.5390000000000000
C	6.1689999999999900	-4.3869999999999900	18.1310000000000000
O	6.2619999999999900	-5.7590000000000000	18.4830000000000000
C	4.9720000000000000	-3.7839999999999900	18.8769999999999000
N	4.1989999999999900	-2.7660000000000000	18.0650000000000000
C	4.3719999999999900	-1.4319999999999900	17.9549999999999000
N	3.4889999999999900	-0.8470000000000000	17.1799999999999000
C	2.7589999999999900	-1.8650000000000000	16.7020000000000000
C	1.6410000000000000	-1.8330000000000000	15.7829999999999000
O	1.0520000000000000	-0.8560000000000000	15.3260000000000000
N	1.2909999999999900	-3.1200000000000000	15.4600000000000000
C	1.8330000000000000	-4.2930000000000000	15.9529999999999000
N	1.3540000000000000	-5.4290000000000000	15.4410000000000000
N	2.7599999999999900	-4.3120000000000000	16.8509999999999000
C	3.2010000000000000	-3.0640000000000000	17.1840000000000000
H	7.2300000000000000	-2.2260000000000000	21.9400000000000000
H	8.6410000000000000	-2.3109999999999900	20.9100000000000000
H	7.1040000000000000	-4.3140000000000000	20.6750000000000000
H	7.2640000000000000	-2.5169999999999900	18.2130000000000000
H	6.0389999999999900	-4.2679999999999900	17.0569999999999000
H	5.5860000000000000	-6.2450000000000000	18.0040000000000000
H	4.2530000000000000	-4.5419999999999900	19.1969999999999000
H	5.1600000000000000	-0.9320000000000000	18.4980000000000000
H	0.5470000000000000	-3.2040000000000000	14.7780000000000000
H	1.6990000000000000	-6.3150000000000000	15.7799999999999000
H	0.6810000000000000	-5.4210000000000000	14.6850000000000000
H	8.6519999999999900	1.0469999999999900	19.1969999999999000
P	9.3490000000000000	-4.0830000000000000	17.0869999999999000
O	10.7360000000000000	-4.6210000000000000	17.1069999999999000
O	9.2330000000000000	-2.5609999999999900	16.5380000000000000
O	8.2980000000000000	-4.8860000000000000	16.1419999999999000
C	8.3529999999999900	-6.2960000000000000	16.0159999999999000
C	7.5369999999999900	-6.7100000000000000	14.7840000000000000
O	6.1870000000000000	-6.2679999999999900	14.8989999999999000
C	7.9459999999999900	-6.0039999999999900	13.5079999999999000
O	9.1869999999999900	-6.5010000000000000	13.0190000000000000
C	6.7320000000000000	-6.1710000000000000	12.6090000000000000
O	6.6070000000000000	-7.4539999999999900	12.0160000000000000
C	5.6139999999999900	-5.9710000000000000	13.6449999999999000
N	5.0549999999999900	-4.5620000000000000	13.6549999999999000
C	5.5330000000000000	-3.4640000000000000	14.2780000000000000
N	4.8520000000000000	-2.3740000000000000	14.0250000000000000

C	3.9020000000000000	-2.7719999999999900	13.1649999999999000
C	2.8710000000000000	-2.0640000000000000	12.5079999999999000
N	2.6480000000000000	-0.7020000000000000	12.6850000000000000
N	2.0630000000000000	-2.7450000000000000	11.6549999999999000
C	2.2879999999999900	-4.0549999999999900	11.4840000000000000
N	3.2189999999999900	-4.8339999999999900	12.0519999999999000
C	4.0039999999999900	-4.1269999999999900	12.9009999999999000
H	7.9770000000000000	-6.7850000000000000	16.9130000000000000
H	9.3840000000000000	-6.6330000000000000	15.8930000000000000
H	7.5549999999999900	-7.7949999999999900	14.6579999999999000
H	8.1080000000000000	-4.9429999999999900	13.7089999999999000
H	6.6859999999999900	-5.3830000000000000	11.8590000000000000
H	7.2400000000000000	-7.5170000000000000	11.2970000000000000
H	4.7729999999999900	-6.6490000000000000	13.4870000000000000
H	6.4039999999999900	-3.5230000000000000	14.9120000000000000
H	1.8430000000000000	-0.2760000000000000	12.2420000000000000
H	3.1410000000000000	-0.2240000000000000	13.4250000000000000
H	1.6230000000000000	-4.5519999999999900	10.7929999999999000
H	10.0809999999999000	-2.1469999999999900	16.2880000000000000
P	9.8910000000000000	-5.8719999999999900	11.6899999999999000
O	11.2400000000000000	-6.4070000000000000	11.3670000000000000
O	9.8490000000000000	-4.2720000000000000	11.9719999999999000
O	8.7690000000000000	-6.0930000000000000	10.5350000000000000
C	8.7820000000000000	-5.2900000000000000	9.3750000000000000
C	7.4649999999999900	-5.4889999999999900	8.6140000000000000
O	6.3360000000000000	-5.1749999999999900	9.4230000000000000
C	7.3029999999999900	-4.5300000000000000	7.4509999999999900
O	8.0060000000000000	-5.0690000000000000	6.3410000000000000
C	5.7990000000000000	-4.4320000000000000	7.2580000000000000
O	5.1929999999999900	-5.5469999999999900	6.6239999999999900
C	5.3799999999999900	-4.3970000000000000	8.7430000000000000
N	5.2050000000000000	-3.0819999999999900	9.3729999999999900
C	4.1779999999999900	-2.2599999999999900	8.9290000000000000
O	3.3889999999999900	-2.6840000000000000	8.0830000000000000
N	4.1180000000000000	-0.9670000000000000	9.4879999999999900
C	5.0170000000000000	-0.5190000000000000	10.2919999999999000
N	4.9359999999999900	0.7130000000000000	10.8010000000000000
C	6.1239999999999900	-1.3250000000000000	10.6969999999999000
C	6.1669999999999900	-2.5760000000000000	10.2200000000000000
H	9.6289999999999900	-5.5800000000000000	8.7509999999999900
H	8.9139999999999900	-4.2389999999999900	9.6349999999999900
H	7.3680000000000000	-6.5250000000000000	8.2829999999999900
H	7.6989999999999900	-3.5470000000000000	7.7149999999999900
H	5.5270000000000000	-3.5089999999999900	6.7469999999999900
H	5.3990000000000000	-5.5170000000000000	5.6870000000000000
H	4.4160000000000000	-4.8860000000000000	8.9160000000000000
H	5.6289999999999900	1.0449999999999900	11.4540000000000000
H	4.1490000000000000	1.3060000000000000	10.5679999999999000
H	6.8920000000000000	-0.9700000000000000	11.3689999999999000

H	6.9610000000000000	-3.2540000000000000	10.4920000000000000
H	10.7219999999999000	-3.8340000000000000	11.9939999999999000
P	8.9450000000000000	-4.1399999999999900	5.3849999999999900
O	9.8729999999999900	-4.8819999999999900	4.4889999999999900
O	9.6679999999999900	-3.1019999999999900	6.4050000000000000
O	7.8410000000000000	-3.2240000000000000	4.6250000000000000
C	6.9500000000000000	-3.8510000000000000	3.7200000000000000
C	5.8890000000000000	-2.8540000000000000	3.2440000000000000
O	5.0910000000000000	-2.4159999999999900	4.3339999999999900
C	6.4440000000000000	-1.5380000000000000	2.7380000000000000
O	6.9749999999999900	-1.7250000000000000	1.4319999999999900
C	5.2409999999999900	-0.6140000000000000	2.8279999999999900
O	4.2629999999999900	-0.7950000000000000	1.8160000000000000
C	4.6639999999999900	-1.0860000000000000	4.1749999999999900
N	5.1150000000000000	-0.2680000000000000	5.3670000000000000
C	6.0700000000000000	-0.5300000000000000	6.2839999999999900
N	6.1840000000000000	0.3940000000000000	7.2050000000000000
C	5.2800000000000000	1.3220000000000000	6.8550000000000000
C	4.9119999999999900	2.5529999999999900	7.4420000000000000
N	5.4610000000000000	3.0339999999999900	8.6259999999999900
N	3.9720000000000000	3.3090000000000000	6.8159999999999900
C	3.4359999999999900	2.8410000000000000	5.6820000000000000
N	3.6829999999999900	1.6890000000000000	5.0430000000000000
C	4.6230000000000000	0.9600000000000000	5.6929999999999900
H	6.4640000000000000	-4.7089999999999900	4.1840000000000000
H	7.5090000000000000	-4.2320000000000000	2.8630000000000000
H	5.2389999999999900	-3.3210000000000000	2.5019999999999900
H	7.2380000000000000	-1.1850000000000000	3.3990000000000000
H	5.5410000000000000	0.4290000000000000	2.8919999999999900
H	3.6139999999999900	-0.0900000000000000	1.8870000000000000
H	3.5720000000000000	-1.0720000000000000	4.1840000000000000
H	6.6459999999999900	-1.4419999999999900	6.2370000000000000
H	5.0999999999999900	3.8929999999999900	9.0199999999999900
H	6.1059999999999900	2.4510000000000000	9.1389999999999900
H	2.6930000000000000	3.4750000000000000	5.2210000000000000
H	10.6379999999999000	-3.0379999999999900	6.3129999999999900
P	8.1630000000000000	-0.7750000000000000	0.8390000000000000
O	8.7400000000000000	-1.2200000000000000	-0.4580000000000000
O	9.2340000000000000	-0.6820000000000000	2.0550000000000000
O	7.4909999999999900	0.7040000000000000	0.8210000000000000
C	6.5039999999999900	1.0080000000000000	-0.1470000000000000
C	5.9310000000000000	2.4070000000000000	0.1110000000000000
O	5.3579999999999900	2.4830000000000000	1.4110000000000000
C	6.9610000000000000	3.5219999999999900	0.1790000000000000
O	7.3929999999999900	3.8660000000000000	-1.1319999999999900
C	6.2130000000000000	4.6319999999999900	0.9050000000000000
O	5.2519999999999900	5.3250000000000000	0.1230000000000000
C	5.4779999999999900	3.7759999999999900	1.9560000000000000
N	6.1307921310805700	3.7898352469092500	3.2547890128433900

C	5.7757845483716400	4.8259656724805200	4.1054568958841600
O	4.9957668502963600	5.7062223885932700	3.7572466867293900
N	6.3498226301104400	4.8115108976770400	5.3311497602106400
C	7.3063543283349500	3.9105617962880500	5.7823734932240300
O	7.7608571206423900	4.0235207842236400	6.9047455301323200
C	7.6446881638304900	2.8785791076423400	4.8328750192407300
C	7.0549859352424700	2.8537792433723500	3.6263594578015200
H	5.7050000000000000	0.2670000000000000	-0.1260000000000000
H	6.9409999999999900	0.9640000000000000	-1.1459999999999900
H	5.1600000000000000	2.6360000000000000	-0.6280000000000000
H	7.8220000000000000	3.1970000000000000	0.7660000000000000
H	6.9009999999999900	5.3239999999999900	1.3870000000000000
H	5.7080000000000000	5.9450000000000000	-0.4520000000000000
H	4.4489999999999900	4.1109999999999900	2.1250000000000000
H	6.1097743568669600	5.6146182878387300	5.9517844366156600
H	8.3823631717623400	2.1491034699198400	5.1164050746332500
H	7.2673686806585600	2.0976326309381700	2.8839538179989400
H	10.1379999999999000	-0.9760000000000000	1.8340000000000000
P	8.9000000000000000	4.4219999999999900	-1.4259999999999900
O	9.2219999999999900	4.6260000000000000	-2.8630000000000000
O	9.8689999999999900	3.3719999999999900	-0.6530000000000000
O	8.9949999999999900	5.7640000000000000	-0.5100000000000000
C	8.2889999999999900	6.9119999999999900	-0.9419999999999990
C	8.3237448165107800	8.0560283502303900	0.0699993763303400
O	7.7336522742397500	7.5887761265912800	1.3099519045985100
C	9.7050986540758500	8.5172266549601400	0.5303282954252400
O	10.3426025774543000	9.4131186304496800	-0.3913526287873400
C	9.3759996356465000	9.2510509075243800	1.8465700357076500
O	9.0348476330692100	10.6065737470166000	1.6282837574963900
C	8.1281300342740400	8.4838498802105700	2.3447034786004600
N	8.4311865706687300	7.6687903638001600	3.5102597034758700
C	8.7393901909046800	6.3241147338629800	3.5162233129687500
N	8.9982702882563800	5.8547430730058600	4.7102612515599400
C	8.8619073856980900	6.9451412562134100	5.5419625008560800
C	9.0321056578372900	7.0992575188293400	6.9336997193834600
N	9.3402808203837900	6.1113180189672000	7.7659841513860800
N	8.8703175515146500	8.3545079642713800	7.4371605928891800
C	8.5423541396410800	9.3561631364862600	6.6256019910958200
N	8.3278890784134800	9.3304953379186800	5.3023071923797200
C	8.5065577481300400	8.0854700333992300	4.8222911878456500
H	7.2420000000000000	6.6689999999999900	-1.1200000000000000
H	8.6859999999999900	7.2539999999999900	-1.8990000000000000
H	7.7240050249480700	8.8909694428354500	-0.3315150710776500
H	10.3235400869138000	7.6394172660518500	0.7325563670933300
H	10.1599720498311000	9.1671332141166400	2.6097012615927600
H	9.7510576887180400	10.9717746412036000	1.0752512832456700
H	7.3118125440129100	9.1678018873582300	2.5991496917692700
H	8.7415213642633000	5.7874382789626100	2.5839760473875500
H	9.2961257804446100	6.3302557323869300	8.7598876797256700

H	9.1364131847123900	5.1658512750815700	7.4475810610312000
H	8.4366506496422900	10.3285047513940000	7.1110050917297700
H	9.6929999999999900	3.2440000000000000	0.2980000000000000
P	11.7583083135346000	9.1141079024753500	-1.1024002974188300
O	12.3344570098800000	10.3395804379235000	-1.7008128037549300
O	11.5349566015406000	7.9883579362143400	-2.2161013271363300
O	12.5592751839607000	8.4414903912415100	0.1079294520677600
C	13.8538749542806000	7.8024790251638800	-0.0602309609935900
C	14.4808503690711000	7.6338422453369200	1.3071014977635900
O	14.9352667902728000	8.9282716319303200	1.7943068924777800
C	13.5286726596892000	7.0627664408092900	2.3716416897168300
O	14.2190528247043000	6.1724353255516000	3.2734237204326300
C	13.1009712059712000	8.3070546230351100	3.1432788209104800
O	12.6182666498958000	8.0226059349987700	4.4268250246375100
C	14.3635614666176000	9.1939582463550200	3.0701597388491300
N	14.0513010748329000	10.6254557945051000	3.1323431924858300
C	14.3552110333007000	11.3389993398881000	4.2903882659091000
O	14.8807534119230000	10.8135774302719000	5.2737484259871500
N	13.9988450063799000	12.6640257392722000	4.2565625676339500
C	13.2705385360865000	13.3118611187911000	3.2663819306908100
O	12.9076261539945000	14.4920008930887000	3.4170334427571900
C	12.9874276039067000	12.5058867027439000	2.1095706177863800
C	13.3954415548632000	11.2161775532026000	2.0852366164415800
H	14.5041298286370000	8.4213787964008100	-0.6880190673259200
H	13.7109375697438000	6.8146565025569100	-0.5122592919460500
H	15.3634091694475000	6.9871223712466000	1.1988022322822000
H	12.6878398247764000	6.5211806832841200	1.9321563797578900
H	12.3476274344802000	8.8029951830259200	2.5164027342855600
H	12.2146287317650000	8.8428822502279000	4.7869036811025200
H	15.0561031972950000	8.9876095593180000	3.8941498613973700
H	14.1450509019368000	13.1703773181825000	5.1527298104769600
H	12.4235316427305000	12.9232337900764000	1.2868475513324400
H	13.2439125408177000	10.5694524618071000	1.2280933539910800
H	11.2561174653363000	7.1213544238267400	-1.8697612544530400
P	14.5397790091535000	4.6567382750655700	2.8121370627343500
O	13.7770273174181000	4.1906215551292200	1.6229728268272600
O	14.3821911359682000	3.8514324335309000	4.1744674596225800
O	16.1186896526200000	4.5765868720540600	2.5561126090545700
C	17.0033522882560000	5.2162197193998700	3.5270215684321800
C	18.2491712829552000	5.6678628287819900	2.7992715455563000
O	17.8464282044588000	6.4126500441729300	1.6483974438464700
C	19.0830722861417000	6.6800864164992300	3.5900534184691700
O	19.9761333522008000	6.0283423367250400	4.5129160801015700
C	19.8544388475240000	7.4307167339638100	2.4937108638186700
O	21.0576487470151000	6.7577364382813700	2.1452228715173600
C	18.8865451008099000	7.3498190185652800	1.3040604807862700
N	18.2740000000000000	8.5990000000000000	0.9700000000000000
C	17.8880000000000000	8.9670000000000000	-0.2680000000000000
N	17.3930000000000000	10.1769999999999000	-0.3330000000000000

C	17.4050000000000000	10.6050000000000000	0.9389999999999990
C	17.0189999999999000	11.8330000000000000	1.5200000000000000
N	16.5350000000000000	12.9060000000000000	0.7850000000000000
N	17.1380000000000000	11.9809999999999000	2.8660000000000000
C	17.5930000000000000	10.9399999999999000	3.5710000000000000
N	17.9939999999999000	9.7379999999999900	3.1370000000000000
C	17.8949999999999000	9.6300000000000000	1.7900000000000000
H	17.2597458600090000	4.4694629809313700	4.2870210913178500
H	16.4968631214762000	6.0854643724710300	3.9615609314300000
H	18.8748769746511000	4.8117809975024400	2.4992323814169100
H	18.4214008128976000	7.4065683655653000	4.0755089653345500
H	20.0308002624441000	8.4792264429250000	2.7730444248736700
H	21.4300162150361000	6.4342128529943600	2.9822217084847700
H	19.4322902862351000	6.9895306663622200	0.4186670863923000
H	18.0210000000000000	8.2989999999999900	-1.1070000000000000
H	16.2979999999999000	13.7639999999999000	1.2660000000000000
H	16.4009999999999000	12.8100000000000000	-0.2120000000000000
H	17.6589999999999000	11.0960000000000000	4.6390000000000000
H	13.7247139708508000	4.2475132118761100	4.7734963936854800
P	20.0986638921182000	6.5522216198646000	6.0456010921927800
O	21.1597940738430000	5.7866254334998500	6.7480415434096600
O	18.6150461377701000	6.5337057120219200	6.6180600752384700
O	20.4294499391842000	8.1117506088269200	5.9615432734174200
C	19.4600122474505000	9.1847339240748800	5.9743425535163500
C	19.4020095491056000	9.8468745055138200	7.3523135681022500
O	18.5687929942214000	9.0749544482311100	8.1995389063882800
C	20.7412592841707000	9.9494350929959100	8.0972946227743200
O	21.4124584564691000	11.1149160252214000	7.7466825540613600
C	20.2969211147477000	9.9765484176585000	9.5816472493626300
O	20.2718479286965000	11.2681604670687000	10.1039101326105000
C	18.8727959753804000	9.3488884540877700	9.5367827635338600
N	18.7819928670173000	8.1175309848049200	10.2825431184643000
C	18.4431833956539000	6.8824261558080300	9.7937367623271100
N	18.4144855650214000	5.9520177697427900	10.6961610550717000
C	18.7404203244948000	6.5802888991042800	11.8693697214335000
C	18.7760130674746000	6.1549501607394300	13.2112306498846000
N	18.5347844093072000	4.9042780168796600	13.5947337905962000
N	19.0439553455124000	7.0845258399665900	14.1485957543446000
C	19.2697969802560000	8.3306088260070400	13.7799568007634000
N	19.2451067419981000	8.8563310109970400	12.5663558678252000
C	18.9676812007705000	7.9444928470768400	11.6228173936628000
H	18.4472391697037000	8.8271661639800400	5.7826825516190600
H	19.7536649659737000	9.8477958851503100	5.1529090832394900
H	18.9852242986011000	10.8613038097090000	7.2399377589434700
H	21.3578781454099000	9.0630499813558100	7.9238005702711200
H	20.9375366250546000	9.3460739600644900	10.2110660706203000
H	20.6441690076320000	11.8655706834976000	9.4219716408353600
H	18.1577046655796000	10.0681956554008000	9.9777556851908900
H	18.2115602527277000	6.7430666435395100	8.7596356081965300

H	18.3836804036527000	4.7277043838370200	14.5759730759363000
H	18.2647707019221000	4.2337607264008500	12.8933917566424000
H	19.4920842528941000	9.0302001257602500	14.5772378045042000
H	18.1566048051211000	5.6871973848213900	6.4631423881478900
P	22.9109012833335000	11.0631140180607000	7.1385467391778100
O	23.7343199827383000	9.9806881656717500	7.6942808463157400
O	23.3743205747271000	12.5189958719276000	7.3984687797504900
O	22.7563157409083000	11.0307233481929000	5.5427789942848600
C	22.8833321317789000	9.8603877133377100	4.7496556149907200
C	22.3829999999999000	10.1809999999999000	3.3370000000000000
O	21.0390000000000000	10.6460000000000000	3.3780000000000000
C	23.1159999999999000	11.3339999999999000	2.6680000000000000
O	24.3079999999999000	10.8439999999999000	2.0619999999999900
C	22.0940000000000000	11.8710000000000000	1.6759999999999900
O	21.9130000000000000	11.0820000000000000	0.5100000000000000
C	20.8219999999999000	11.7509999999999000	2.5350000000000000
N	20.3640000000000000	12.9280000000000000	3.2759999999999900
C	20.0090000000000000	14.0470000000000000	2.5440000000000000
O	19.9220000000000000	14.1099999999999000	1.3180000000000000
N	19.7480000000000000	15.1530000000000000	3.3069999999999900
C	19.7899999999999000	15.3209999999999000	4.6580000000000000
O	19.5360000000000000	16.3909999999999000	5.2050000000000000
C	20.1589999999999000	14.1059999999999000	5.3579999999999900
C	20.4299999999999000	12.9909999999999000	4.6529999999999900
H	22.2994957724841000	9.0407205424881700	5.1710536417979700
H	23.9328142037558000	9.5612461288987600	4.6911064994983300
H	22.4179999999999000	9.2940000000000000	2.7000000000000000
H	23.3739999999999000	12.0969999999999000	3.4060000000000000
H	22.2979999999999000	12.9060000000000000	1.4199999999999900
H	22.6619999999999000	11.2159999999999000	-0.0720000000000000
H	19.9510000000000000	11.5060000000000000	1.9239999999999900
H	19.4759999999999000	15.9770000000000000	2.7869999999999900
H	20.1939999999999000	14.1010000000000000	6.4349999999999900
H	20.6789999999999000	12.0649999999999000	5.1449999999999900
H	22.6822691025870000	13.1751900947742000	7.2198487067926100
P	25.5979999999999000	11.7989999999999000	1.7640000000000000
O	26.7970000000000000	11.0920000000000000	1.2370000000000000
O	25.8380000000000000	12.5690000000000000	3.1720000000000000
O	25.0070000000000000	12.9700000000000000	0.8020000000000000
C	24.7549999999999000	12.7210000000000000	-0.5700000000000000
C	24.1269999999999000	13.9680000000000000	-1.2160000000000000
O	22.9570000000000000	14.3620000000000000	-0.5050000000000000
C	24.9690000000000000	15.2279999999999000	-1.1240000000000000
O	26.0479999999999000	15.1820000000000000	-2.0529999999999900
C	23.9549999999999000	16.3509999999999000	-1.3089999999999900
O	23.5100000000000000	16.5509999999999000	-2.6400000000000000
C	22.7869999999999000	15.7629999999999000	-0.4920000000000000
N	22.6140000000000000	16.2869999999999000	0.8660000000000000
C	22.1269999999999000	17.5809999999999000	1.0229999999999900

O	21.8290000000000000	18.2340000000000000	0.0210000000000000
N	22.0309999999999000	18.0549999999999000	2.3470000000000000
C	22.5459999999999000	17.4280000000000000	3.3430000000000000
N	22.4950000000000000	17.9400000000000000	4.5750000000000000
C	23.1810000000000000	16.1570000000000000	3.1770000000000000
C	23.1859999999999000	15.6440000000000000	1.9399999999999900
H	24.0949999999999000	11.8640000000000000	-0.6860000000000000
H	25.6849999999999000	12.4640000000000000	-1.0800000000000000
H	23.8619999999999000	13.7609999999999000	-2.2549999999999900
H	25.4200000000000000	15.2919999999999000	-0.1320000000000000
H	24.3039999999999000	17.2809999999999000	-0.8670000000000000
H	22.9589999999999000	17.3380000000000000	-2.6640000000000000
H	21.8129999999999000	15.9320000000000000	-0.9620000000000000
H	22.8949999999999000	17.4490000000000000	5.3570000000000000
H	22.0640000000000000	18.8410000000000000	4.7169999999999900
H	23.6370000000000000	15.6189999999999000	3.9940000000000000
H	23.6389999999999000	14.6929999999999000	1.7180000000000000
H	26.7540000000000000	12.5389999999999000	3.5089999999999900
P	27.1819999999999000	16.3550000000000000	-2.1379999999999900
O	28.3309999999999000	16.0640000000000000	-3.0369999999999900
O	27.5919999999999000	16.5910000000000000	-0.5850000000000000
O	26.3369999999999000	17.6980000000000000	-2.4960000000000000
C	26.1419999999999000	18.1030000000000000	-3.8410000000000000
C	25.8689999999999000	19.6140000000000000	-3.8740000000000000
O	24.7139999999999000	19.9130000000000000	-3.0990000000000000
C	26.9080000000000000	20.4669999999999000	-3.1680000000000000
O	28.0869999999999000	20.6020000000000000	-3.9550000000000000
C	26.1589999999999000	21.7590000000000000	-2.8790000000000000
O	26.0210000000000000	22.6219999999999000	-3.9960000000000000
C	24.7860000000000000	21.1840000000000000	-2.4990000000000000
N	24.5869999999999000	21.0560000000000000	-1.0020000000000000
C	24.9319999999999000	20.0530000000000000	-0.1670000000000000
N	24.6610000000000000	20.2959999999999000	1.0940000000000000
C	24.1479999999999000	21.5360000000000000	1.0800000000000000
C	23.6950000000000000	22.3520000000000000	2.1880000000000000
O	23.6179999999999000	22.0569999999999000	3.3790000000000000
N	23.3329999999999000	23.5889999999999000	1.7140000000000000
C	23.3509999999999000	24.0309999999999000	0.4030000000000000
N	22.9720000000000000	25.2929999999999000	0.1910000000000000
N	23.7169999999999000	23.2860000000000000	-0.5820000000000000
C	24.1110000000000000	22.0420000000000000	-0.1910000000000000
H	25.3150000000000000	17.5519999999999000	-4.2850000000000000
H	27.0199999999999000	17.8880000000000000	-4.4500000000000000
H	25.7119999999999000	19.9540000000000000	-4.9000000000000000
H	27.2070000000000000	19.9899999999999000	-2.2320000000000000
H	26.5820000000000000	22.2910000000000000	-2.0280000000000000
H	26.8670000000000000	23.0470000000000000	-4.1589999999999900
H	23.9609999999999000	21.7869999999999000	-2.8830000000000000
H	25.3990000000000000	19.1589999999999000	-0.5470000000000000

H	23.0120000000000000	24.2369999999999000	2.4220000000000000
H	22.9639999999999000	25.6619999999999000	-0.7480000000000000
H	22.7100000000000000	25.8990000000000000	0.9600000000000000
H	28.5420000000000000	16.4840000000000000	-0.3860000000000000
P	29.4319999999999000	21.3099999999999000	-3.3610000000000000
O	30.5760000000000000	21.3979999999999000	-4.3070000000000000
O	29.7349999999999000	20.4510000000000000	-2.0160000000000000
O	28.8960000000000000	22.7390000000000000	-2.7940000000000000
C	29.6370000000000000	23.4349999999999000	-1.8129999999999900
C	28.8769999999999000	24.7100000000000000	-1.4239999999999900
O	27.6000000000000000	24.3760000000000000	-0.8810000000000000
C	29.4909999999999000	25.4669999999999000	-0.2600000000000000
O	30.6570000000000000	26.1769999999999000	-0.6720000000000000
C	28.3309999999999000	26.3129999999999000	0.2510000000000000
O	27.9770000000000000	27.4220000000000000	-0.5600000000000000
C	27.2119999999999000	25.2560000000000000	0.1500000000000000
N	26.8750000000000000	24.5390000000000000	1.3830000000000000
C	26.2770000000000000	25.2500000000000000	2.4169999999999900
O	25.9160000000000000	26.4119999999999000	2.2160000000000000
N	26.1340000000000000	24.5779999999999000	3.6469999999999900
C	26.6370000000000000	23.4130000000000000	3.8599999999999900
N	26.5180000000000000	22.8200000000000000	5.0490000000000000
C	27.3249999999999000	22.6990000000000000	2.8290000000000000
C	27.4110000000000000	23.2950000000000000	1.6319999999999900
H	30.6179999999999000	23.6909999999999000	-2.2170000000000000
H	29.7950000000000000	22.8009999999999000	-0.9379999999999990
H	28.7459999999999000	25.3599999999999000	-2.2900000000000000
H	29.8060000000000000	24.7519999999999000	0.5040000000000000
H	28.4810000000000000	26.6230000000000000	1.2809999999999900
H	27.3109999999999000	27.9359999999999000	-0.0970000000000000
H	26.2549999999999000	25.6780000000000000	-0.1720000000000000
H	26.9029999999999000	21.9009999999999000	5.2060000000000000
H	26.0169999999999000	23.2809999999999000	5.7969999999999900
H	27.7519999999999000	21.7190000000000000	2.9830000000000000
H	27.8990000000000000	22.8210000000000000	0.7940000000000000
H	30.5960000000000000	19.9909999999999000	-2.0049999999999900
P	31.7089999999999000	26.8339999999999000	0.3930000000000000
O	32.9769999999999000	27.3419999999999000	-0.1990000000000000
O	31.9110000000000000	25.6679999999999000	1.5040000000000000
O	30.8309999999999000	27.9450000000000000	1.1890000000000000
C	30.5539999999999000	29.1980000000000000	0.5940000000000000
C	29.8410000000000000	30.0990000000000000	1.6130000000000000
O	28.6799999999999000	29.4589999999999000	2.1299999999999900
C	30.6230000000000000	30.3900000000000000	2.8830000000000000
O	31.5500000000000000	31.4379999999999000	2.6290000000000000
C	29.5369999999999000	30.7880000000000000	3.8660000000000000
O	29.0130000000000000	32.0949999999999000	3.6850000000000000
C	28.4680000000000000	29.7489999999999000	3.4920000000000000
N	28.5289999999999000	28.4619999999999000	4.2980000000000000

C	29.0760000000000000	27.2689999999999000	3.9800000000000000
N	28.9220000000000000	26.3490000000000000	4.9000000000000000
C	28.2940000000000000	26.9939999999999000	5.8920000000000000
C	27.8949999999999000	26.4909999999999000	7.1870000000000000
O	27.9639999999999000	25.3410000000000000	7.6139999999999900
N	27.3949999999999000	27.5289999999999000	7.9310000000000000
C	27.2270000000000000	28.8470000000000000	7.5439999999999900
N	26.7270000000000000	29.6750000000000000	8.4629999999999900
N	27.5279999999999000	29.2749999999999000	6.3639999999999900
C	28.0719999999999000	28.3060000000000000	5.5720000000000000
H	29.9390000000000000	29.0659999999999000	-0.2950000000000000
H	31.4759999999999000	29.6769999999999000	0.2630000000000000
H	29.5390000000000000	31.0309999999999000	1.1319999999999900
H	31.1350000000000000	29.4929999999999000	3.2380000000000000
H	29.8539999999999000	30.6479999999999000	4.8979999999999900
H	29.6619999999999000	32.7299999999999000	3.9960000000000000
H	27.4549999999999000	30.1419999999999000	3.6090000000000000
H	29.5790000000000000	27.1189999999999000	3.0409999999999900
H	27.1110000000000000	27.2740000000000000	8.8699999999999900
H	26.5779999999999000	30.6469999999999000	8.2390000000000000
H	26.5240000000000000	29.3449999999999000	9.4009999999999900
H	32.8389999999999000	25.4239999999999000	1.6859999999999900
P	33.1210000000000000	31.3470000000000000	3.0690000000000000
O	34.0339999999999000	32.2650000000000000	2.3380000000000000
O	33.4939999999999000	29.7719999999999000	2.9420000000000000
O	33.0469999999999000	31.5960000000000000	4.6740000000000000
C	32.7070000000000000	32.8870000000000000	5.1559999999999900
C	32.3040000000000000	32.8350000000000000	6.6349999999999900
O	31.1340000000000000	32.0390000000000000	6.7990000000000000
C	33.2790000000000000	32.1240000000000000	7.5599999999999900
O	34.4249999999999000	32.9279999999999000	7.8190000000000000
C	32.4149999999999000	31.8090000000000000	8.7729999999999900
O	32.1150000000000000	32.9149999999999000	9.6110000000000000
C	31.1329999999999000	31.3689999999999000	8.0399999999999900
N	30.9639999999999000	29.9280000000000000	7.8689999999999900
C	30.5869999999999000	29.2029999999999000	8.9830000000000000
O	30.1909999999999000	29.6670000000000000	10.0519999999999000
N	30.7240000000000000	27.8500000000000000	8.8369999999999900
C	31.2220000000000000	27.1230000000000000	7.7969999999999900
O	31.2899999999999000	25.8979999999999000	7.8200000000000000
C	31.6370000000000000	27.9499999999999000	6.6829999999999900
C	31.4939999999999000	29.2860000000000000	6.7679999999999900
H	31.8859999999999000	33.3100000000000000	4.5750000000000000
H	33.5600000000000000	33.5559999999999000	5.0229999999999900
H	32.0900000000000000	33.8440000000000000	6.9960000000000000
H	33.6210000000000000	31.2029999999999000	7.0860000000000000
H	32.8239999999999000	30.9849999999999000	9.3499999999999900
H	32.8810000000000000	33.1019999999999000	10.1570000000000000
H	30.2210000000000000	31.6980000000000000	8.5470000000000000

H	30.4340000000000000	27.3069999999999000	9.6400000000000000
H	32.0519999999999000	27.4879999999999000	5.7999999999999900
H	31.7929999999999000	29.9370000000000000	5.9630000000000000
H	34.2389999999999000	29.5689999999999000	2.3430000000000000
P	35.8310000000000000	32.2929999999999000	8.3640000000000000
O	36.9889999999999000	33.2269999999999000	8.3520000000000000
O	36.0349999999999000	30.9619999999999000	7.4569999999999900
O	35.4549999999999000	31.6870000000000000	9.8260000000000000
C	35.2719999999999000	32.5690000000000000	10.9179999999999000
C	34.7069999999999000	31.8120000000000000	12.1280000000000000
O	33.5499999999999000	31.0710000000000000	11.7460000000000000
C	35.5790000000000000	30.7049999999999000	12.6969999999999000
O	36.6469999999999000	31.2160000000000000	13.4870000000000000
C	34.5630000000000000	29.8859999999999000	13.4840000000000000
O	34.0709999999999000	30.4899999999999000	14.6730000000000000
C	33.4320000000000000	29.8599999999999000	12.4529999999999000
N	33.5129999999999000	28.6709999999999000	11.5139999999999000
C	33.9819999999999000	28.5730000000000000	10.2509999999999000
N	33.9530000000000000	27.3539999999999000	9.7699999999999900
C	33.4819999999999000	26.6149999999999000	10.7840000000000000
C	33.2719999999999000	25.1829999999999000	10.8559999999999000
O	33.3870000000000000	24.3430000000000000	9.9680000000000000
N	32.9099999999999000	24.8490000000000000	12.1379999999999000
C	32.7090000000000000	25.6990000000000000	13.2100000000000000
N	32.3840000000000000	25.1329999999999000	14.3729999999999000
N	32.8250000000000000	26.9800000000000000	13.1170000000000000
C	33.2239999999999000	27.3900000000000000	11.8789999999999000
H	34.5880000000000000	33.3710000000000000	10.6359999999999000
H	36.2180000000000000	33.0469999999999000	11.1780000000000000
H	34.4399999999999000	32.5249999999999000	12.9100000000000000
H	36.0150000000000000	30.1230000000000000	11.8829999999999000
H	34.9099999999999000	28.8719999999999000	13.6790000000000000
H	33.5270000000000000	31.2450000000000000	14.4369999999999000
H	32.4440000000000000	29.8200000000000000	12.9190000000000000
H	34.3500000000000000	29.4379999999999000	9.7240000000000000
H	32.7710000000000000	23.8590000000000000	12.2959999999999000
H	32.2160000000000000	25.7139999999999000	15.1799999999999000
H	32.3280000000000000	24.1239999999999000	14.4680000000000000
H	36.8920000000000000	30.9029999999999000	6.9930000000000000
P	37.9219999999999000	30.2830000000000000	13.9120000000000000
O	38.9549999999999000	30.9589999999999000	14.7420000000000000
O	38.4699999999999000	29.7310000000000000	12.4870000000000000
O	37.2550000000000000	28.9540000000000000	14.5770000000000000
C	36.8179999999999000	28.9630000000000000	15.9250000000000000
C	36.4620000000000000	27.5369999999999000	16.3700000000000000
O	35.5200000000000000	26.9269999999999000	15.4890000000000000
C	37.6059999999999000	26.5489999999999000	16.2800000000000000
O	38.5579999999999000	26.7860000000000000	17.3099999999999000
C	36.8849999999999000	25.2079999999999000	16.3210000000000000

O	36.3329999999999000	24.8460000000000000	17.5770000000000000
C	35.7259999999999000	25.5339999999999000	15.3680000000000000
N	36.0099999999999000	25.1709999999999000	13.9230000000000000
C	36.4059999999999000	25.9639999999999000	12.9030000000000000
N	36.6090000000000000	25.3170000000000000	11.7810000000000000
C	36.3459999999999000	24.0390000000000000	12.0879999999999000
C	36.4099999999999000	22.8649999999999000	11.2390000000000000
O	36.7010000000000000	22.7890000000000000	10.0480000000000000
N	36.0949999999999000	21.7560000000000000	11.9830000000000000
C	35.7859999999999000	21.7020000000000000	13.3300000000000000
N	35.5989999999999000	20.4789999999999000	13.8399999999999000
N	35.6929999999999000	22.7549999999999000	14.0730000000000000
C	35.9869999999999000	23.9080000000000000	13.4009999999999000
H	35.9630000000000000	29.6219999999999000	16.0489999999999000
H	37.6000000000000000	29.3610000000000000	16.5740000000000000
H	36.0499999999999000	27.5590000000000000	17.3810000000000000
H	38.1229999999999000	26.6789999999999000	15.3260000000000000
H	37.4930000000000000	24.4020000000000000	15.9179999999999000
H	37.0369999999999000	24.5090000000000000	18.1350000000000000
H	34.8059999999999000	25.0159999999999000	15.6470000000000000
H	36.5279999999999000	27.0279999999999000	13.0440000000000000
H	36.1280000000000000	20.8870000000000000	11.4730000000000000
H	35.3830000000000000	20.3769999999999000	14.8190000000000000
H	35.6769999999999000	19.6550000000000000	13.2620000000000000
H	39.4139999999999000	29.9050000000000000	12.3109999999999000
P	40.0390000000000000	26.1000000000000000	17.2979999999999000
O	40.9819999999999000	26.6140000000000000	18.3279999999999000
O	40.5429999999999000	26.2910000000000000	15.7680000000000000
O	39.7269999999999000	24.5070000000000000	17.3870000000000000
C	39.3449999999999000	23.9469999999999000	18.6290000000000000
C	39.1899999999999000	22.4299999999999000	18.4849999999999000
O	38.3179999999999000	22.0949999999999000	17.4100000000000000
C	40.4530000000000000	21.6990000000000000	18.0740000000000000
O	41.3040000000000000	21.5799999999999000	19.2079999999999000
C	39.9029999999999000	20.3700000000000000	17.5859999999999000
O	39.4939999999999000	19.4950000000000000	18.6250000000000000
C	38.6599999999999000	20.8590000000000000	16.8260000000000000
N	38.8609999999999000	21.0279999999999000	15.3300000000000000
C	39.0260000000000000	22.1580000000000000	14.6120000000000000
N	39.1169999999999000	21.9559999999999000	13.3209999999999000
C	39.0679999999999000	20.6219999999999000	13.1899999999999000
C	39.1240000000000000	19.7830000000000000	12.0530000000000000
N	39.2070000000000000	20.2650000000000000	10.7509999999999000
N	39.1189999999999000	18.4370000000000000	12.2420000000000000
C	39.0480000000000000	17.9819999999999000	13.4990000000000000
N	38.9399999999999000	18.6720000000000000	14.6449999999999000
C	38.9540000000000000	20.0079999999999000	14.4239999999999000
H	38.4189999999999000	24.3949999999999000	18.9830000000000000
H	40.0959999999999000	24.1660000000000000	19.3900000000000000

H	38.7899999999999000	22.0139999999999000	19.4119999999999000
H	40.9620000000000000	22.2360000000000000	17.2699999999999000
H	40.5859999999999000	19.8739999999999000	16.9020000000000000
H	39.1569999999999000	18.6840000000000000	18.2250000000000000
H	37.8119999999999000	20.1810000000000000	16.9430000000000000
H	39.0519999999999000	23.1219999999999000	15.0960000000000000
H	39.2349999999999000	19.6000000000000000	9.9879999999999900
H	39.1659999999999000	21.2609999999999000	10.5899999999999000
H	39.0989999999999000	16.9100000000000000	13.6099999999999000
H	41.3950000000000000	26.7579999999999000	15.6679999999999000
P	42.9320000000000000	21.6239999999999000	19.0910000000000000
O	43.6529999999999000	21.7100000000000000	20.3889999999999000
O	43.2189999999999000	22.8560000000000000	18.0740000000000000
O	43.2849999999999000	20.3109999999999000	18.1980000000000000
C	43.0319999999999000	19.0169999999999000	18.7180000000000000
C	43.2340000000000000	17.9720000000000000	17.6129999999999000
O	42.4169999999999000	18.3309999999999000	16.5000000000000000
C	44.6610000000000000	17.9059999999999000	17.0530000000000000
O	45.0219999999999000	16.5450000000000000	16.8859999999999000
C	44.6000000000000000	18.6090000000000000	15.7110000000000000
O	45.4780000000000000	18.1769999999999000	14.6920000000000000
C	43.1529999999999000	18.3449999999999000	15.3140000000000000
N	42.6090000000000000	19.2979999999999000	14.3590000000000000
C	42.5319999999999000	18.9310000000000000	13.0310000000000000
O	42.5910000000000000	17.7890000000000000	12.5790000000000000
N	42.4179999999999000	19.9899999999999000	12.1739999999999000
C	42.3710000000000000	21.3279999999999000	12.4250000000000000
O	42.2989999999999000	22.1660000000000000	11.5299999999999000
C	42.4020000000000000	21.6310000000000000	13.8430000000000000
C	42.5089999999999000	20.6219999999999000	14.7279999999999000
H	42.0129999999999000	18.9529999999999000	19.1000000000000000
H	43.6910000000000000	18.8120000000000000	19.5629999999999000
H	42.9229999999999000	16.9959999999999000	17.9920000000000000
H	45.4089999999999000	18.3859999999999000	17.6890000000000000
H	44.7340000000000000	19.6789999999999000	15.8750000000000000
H	46.3779999999999000	18.4319999999999000	14.9260000000000000
H	43.0450000000000000	17.3389999999999000	14.8970000000000000
H	42.3939999999999000	19.7369999999999000	11.1940000000000000
H	42.3359999999999000	22.6580000000000000	14.1720000000000000
H	42.5480000000000000	20.7850000000000000	15.7959999999999000
H	43.7790000000000000	23.5689999999999000	18.4349999999999000
P	45.8089999999999000	15.7750000000000000	18.0859999999999000
O	45.4390000000000000	16.1829999999999000	19.4699999999999000
O	47.3689999999999000	15.9990000000000000	17.7070000000000000
O	45.5579999999999000	14.2119999999999000	17.7270000000000000
C	44.3890000000000000	13.5670000000000000	18.1990000000000000
C	44.5399999999999000	12.0470000000000000	18.0489999999999000
O	44.5020000000000000	11.7070000000000000	16.6720000000000000
C	45.8770000000000000	11.4879999999999000	18.5199999999999000

O	45.6499999999999000	10.2059999999999000	19.0919999999999000
C	46.7399999999999000	11.3889999999999000	17.2680000000000000
O	47.7139999999999000	10.3610000000000000	17.3019999999999000
C	45.6910000000000000	11.1560000000000000	16.1780000000000000
N	46.0150000000000000	11.8030000000000000	14.8450000000000000
C	45.7610000000000000	13.0649999999999000	14.4410000000000000
N	46.2040000000000000	13.3330000000000000	13.2379999999999000
C	46.7980000000000000	12.1959999999999000	12.8439999999999000
C	47.4849999999999000	11.8539999999999000	11.6560000000000000
N	47.7000000000000000	12.7449999999999000	10.6120000000000000
N	47.9650000000000000	10.5890000000000000	11.5250000000000000
C	47.7689999999999000	9.7379999999999900	12.5419999999999000
N	47.1679999999999000	9.9489999999999900	13.7219999999999000
C	46.6929999999999000	11.2159999999999000	13.8149999999999000
H	43.5149999999999000	13.9309999999999000	17.6580000000000000
H	44.2250000000000000	13.8019999999999000	19.2530000000000000
H	43.6950000000000000	11.5640000000000000	18.5420000000000000
H	46.3680000000000000	12.1199999999999000	19.2650000000000000
H	47.2220000000000000	12.3510000000000000	17.0829999999999000
H	48.3229999999999000	10.5459999999999000	18.0210000000000000
H	45.5119999999999000	10.0930000000000000	16.0030000000000000
H	45.2349999999999000	13.7550000000000000	15.0860000000000000
H	48.2790000000000000	12.4529999999999000	9.8390000000000000
H	47.4200000000000000	13.7119999999999000	10.7270000000000000
H	48.1629999999999000	8.7439999999999900	12.3930000000000000
H	47.9290000000000000	16.3219999999999000	18.4400000000000000
P	45.5709999999999000	10.0530000000000000	20.7130000000000000
O	46.8170000000000000	10.4429999999999000	21.4269999999999000
O	45.1289999999999000	8.5039999999999900	20.9149999999999000
O	44.2529999999999000	10.9410000000000000	21.1020000000000000
C	42.9900000000000000	10.3640000000000000	21.4089999999999000
C	42.2689999999999000	11.1929999999999000	22.4879999999999000
O	43.1280000000000000	11.2949999999999000	23.6260000000000000
C	41.9560000000000000	12.6530000000000000	22.1219999999999000
O	40.7710000000000000	13.0389999999999000	22.8180000000000000
C	43.1210000000000000	13.4380000000000000	22.6990000000000000
O	42.9109999999999000	14.8179999999999000	22.9439999999999000
C	43.3310000000000000	12.6329999999999000	23.9830000000000000
N	44.5970000000000000	12.7980000000000000	24.6849999999999000
C	44.6069999999999000	13.1980000000000000	26.0079999999999000
O	43.6310000000000000	13.4979999999999000	26.6939999999999000
N	45.8590000000000000	13.2530000000000000	26.5599999999999000
C	47.0739999999999000	12.9680000000000000	26.0100000000000000
O	48.1250000000000000	13.0649999999999000	26.6370000000000000
C	46.9799999999999000	12.5399999999999000	24.6269999999999000
C	45.7700000000000000	12.4770000000000000	24.0399999999999000
H	43.0949999999999000	9.3469999999999900	21.7890000000000000
H	42.3870000000000000	10.2949999999999000	20.5030000000000000
H	41.3560000000000000	10.6669999999999000	22.7710000000000000

H	41.8290000000000000	12.8369999999999000	21.0530000000000000
H	43.9919999999999000	13.3209999999999000	22.0530000000000000
H	42.0300000000000000	14.9299999999999000	23.3109999999999000
H	42.5279999999999000	12.8699999999999000	24.6870000000000000
H	45.8860000000000000	13.5510000000000000	27.5229999999999000
H	47.8759999999999000	12.2739999999999000	24.0850000000000000
H	45.6379999999999000	12.1560000000000000	23.0169999999999000
H	45.7479999999999000	7.9610000000000000	21.4400000000000000
P	39.3029999999999000	12.9749999999999000	22.1129999999999000
O	38.1469999999999000	13.2100000000000000	23.0180000000000000
O	39.2979999999999000	11.5359999999999000	21.3599999999999000
O	39.4519999999999000	14.0280000000000000	20.8859999999999000
C	39.4099999999999000	15.4250000000000000	21.1099999999999000
C	39.2389999999999000	16.1469999999999000	19.7650000000000000
O	40.3890000000000000	15.9239999999999000	18.9540000000000000
C	38.1099999999999000	15.6250000000000000	18.8840000000000000
O	36.8699999999999000	16.2049999999999000	19.2809999999999000
C	38.5609999999999000	15.9789999999999000	17.4740000000000000
O	38.3439999999999000	17.3369999999999000	17.1419999999999000
C	40.0709999999999000	15.7319999999999000	17.5990000000000000
N	40.5409999999999000	14.3539999999999000	17.1720000000000000
C	40.4359999999999000	13.1869999999999000	17.8419999999999000
N	40.9489999999999000	12.1620000000000000	17.2109999999999000
C	41.3659999999999000	12.6820000000000000	16.0509999999999000
C	42.0020000000000000	12.0039999999999000	14.9459999999999000
O	42.3049999999999000	10.8170000000000000	14.8550000000000000
N	42.2199999999999000	12.9039999999999000	13.9359999999999000
C	41.9219999999999000	14.2530000000000000	13.9269999999999000
N	42.2299999999999000	14.8979999999999000	12.8010000000000000
N	41.3789999999999000	14.8620000000000000	14.9290000000000000
C	41.1129999999999000	14.0250000000000000	15.9749999999999000
H	40.3220000000000000	15.7509999999999000	21.6110000000000000
H	38.5759999999999000	15.6790000000000000	21.7680000000000000
H	39.1409999999999000	17.2199999999999000	19.9299999999999000
H	38.0120000000000000	14.5429999999999000	18.9860000000000000
H	38.1340000000000000	15.3179999999999000	16.7240000000000000
H	38.5170000000000000	17.4649999999999000	16.2029999999999000
H	40.6439999999999000	16.4540000000000000	17.0150000000000000
H	39.9799999999999000	13.1379999999999000	18.8150000000000000
H	42.6450000000000000	12.5120000000000000	13.1050000000000000
H	42.1000000000000000	15.8989999999999000	12.7370000000000000
H	42.6099999999999000	14.3989999999999000	12.0069999999999000
H	38.5210000000000000	10.9740000000000000	21.5450000000000000
P	35.4279999999999000	15.6739999999999000	18.7259999999999000
O	34.2379999999999000	16.3769999999999000	19.2749999999999000
O	35.4720000000000000	14.0839999999999000	19.0509999999999000
O	35.5709999999999000	15.7100000000000000	17.1050000000000000
C	35.1890000000000000	16.8560000000000000	16.3640000000000000
C	35.3609999999999000	16.5710000000000000	14.8620000000000000

O	36.7049999999999000	16.1619999999999000	14.6039999999999000
C	34.5919999999999000	15.3719999999999000	14.3260000000000000
O	33.2269999999999000	15.6460000000000000	14.0320000000000000
C	35.3609999999999000	14.9990000000000000	13.0709999999999000
O	35.1540000000000000	15.8680000000000000	11.9659999999999000
C	36.7869999999999000	15.1739999999999000	13.6020000000000000
N	37.3819999999999000	13.8960000000000000	14.1720000000000000
C	37.0979999999999000	13.2759999999999000	15.3399999999999000
N	37.6649999999999000	12.1069999999999000	15.4760000000000000
C	38.3220000000000000	11.9350000000000000	14.3230000000000000
C	39.0570000000000000	10.7759999999999000	13.8740000000000000
O	39.2449999999999000	9.7119999999999900	14.4610000000000000
N	39.5529999999999000	11.0220000000000000	12.6189999999999000
C	39.3950000000000000	12.1600000000000000	11.8550000000000000
N	39.9729999999999000	12.1310000000000000	10.6530000000000000
N	38.7280000000000000	13.1899999999999000	12.2569999999999000
C	38.1989999999999000	13.0259999999999000	13.5039999999999000
H	35.7899999999999000	17.7160000000000000	16.6589999999999000
H	34.1510000000000000	17.1119999999999000	16.5770000000000000
H	35.1360000000000000	17.4660000000000000	14.2769999999999000
H	34.6409999999999000	14.5630000000000000	15.0549999999999000
H	35.1820000000000000	13.9600000000000000	12.7959999999999000
H	34.2719999999999000	15.7110000000000000	11.6189999999999000
H	37.4659999999999000	15.5250000000000000	12.8239999999999000
H	36.4389999999999000	13.7230000000000000	16.0670000000000000
H	40.0790000000000000	10.2609999999999000	12.2089999999999000
H	39.9170000000000000	12.9380000000000000	10.0429999999999000
H	40.4479999999999000	11.3019999999999000	10.3200000000000000
H	34.7310000000000000	13.7509999999999000	19.5919999999999000
P	32.0660000000000000	14.5860000000000000	14.4770000000000000
O	32.5420000000000000	13.2360000000000000	14.8859999999999000
O	31.0629999999999000	14.5770000000000000	13.1999999999999000
O	31.2390000000000000	15.3979999999999000	15.6229999999999000
C	30.4100000000000000	16.5000000000000000	15.2949999999999000
C	30.8109999999999000	17.7459999999999000	16.0970000000000000
O	32.0409999999999000	18.2789999999999000	15.6150000000000000
C	29.8560000000000000	18.9089999999999000	15.8949999999999000
O	28.6960000000000000	18.7469999999999000	16.7079999999999000
C	30.7059999999999000	20.1359999999999000	16.1859999999999000
O	30.9710000000000000	20.3790000000000000	17.5579999999999000
C	32.0170000000000000	19.6840000000000000	15.5069999999999000
N	32.2670000000000000	20.1409999999999000	14.1349999999999000
C	32.3669999999999000	21.5049999999999000	13.8949999999999000
O	32.3329999999999000	22.2860000000000000	14.8469999999999000
N	32.5230000000000000	21.9009999999999000	12.5510000000000000
C	32.5369999999999000	21.0609999999999000	11.5790000000000000
N	32.7190000000000000	21.4720000000000000	10.3219999999999000
C	32.3750000000000000	19.6570000000000000	11.8030000000000000
C	32.2419999999999000	19.2579999999999000	13.0749999999999000

H	29.3900000000000000	16.2119999999999000	15.5510000000000000
H	30.4170000000000000	16.7250000000000000	14.2270000000000000
H	30.9110000000000000	17.5120000000000000	17.1589999999999000
H	29.5199999999999000	18.9250000000000000	14.8559999999999000
H	30.2940000000000000	21.0219999999999000	15.7100000000000000
H	31.4220000000000000	21.2240000000000000	17.6389999999999000
H	32.9050000000000000	20.0399999999999000	16.0390000000000000
H	32.7349999999999000	20.8140000000000000	9.5589999999999900
H	32.8629999999999000	22.4570000000000000	10.1310000000000000
H	32.3629999999999000	18.9349999999999000	11.0000000000000000
H	32.1270000000000000	18.2149999999999000	13.3330000000000000
H	30.8520000000000000	13.6910000000000000	12.8460000000000000
P	27.3730000000000000	19.6939999999999000	16.5629999999999000
O	26.2450000000000000	19.3460000000000000	17.4690000000000000
O	27.0180000000000000	19.5990000000000000	14.9819999999999000
O	27.9450000000000000	21.2100000000000000	16.7109999999999000
C	27.9780000000000000	21.8700000000000000	17.9660000000000000
C	27.9729999999999000	23.3889999999999000	17.7369999999999000
O	29.0829999999999000	23.7650000000000000	16.9280000000000000
C	26.8120000000000000	23.9089999999999000	16.9070000000000000
O	25.6320000000000000	23.9959999999999000	17.6990000000000000
C	27.3380000000000000	25.2409999999999000	16.3939999999999000
O	27.3599999999999000	26.2899999999999000	17.3490000000000000
C	28.7830000000000000	24.8369999999999000	16.0650000000000000
N	28.9929999999999000	24.4050000000000000	14.6259999999999000
C	28.9460000000000000	23.1690000000000000	14.0879999999999000
N	29.1990000000000000	23.1380000000000000	12.8040000000000000
C	29.3840000000000000	24.4250000000000000	12.4779999999999000
C	29.6969999999999000	25.0479999999999000	11.2490000000000000
N	29.9110000000000000	24.3399999999999000	10.0709999999999000
N	29.8049999999999000	26.4020000000000000	11.2240000000000000
C	29.6069999999999000	27.0740000000000000	12.3659999999999000
N	29.3200000000000000	26.6000000000000000	13.5869999999999000
C	29.2289999999999000	25.2480000000000000	13.5809999999999000
H	28.8619999999999000	21.5680000000000000	18.5259999999999000
H	27.1170000000000000	21.5990000000000000	18.5779999999999000
H	28.0339999999999000	23.9149999999999000	18.6920000000000000
H	26.6149999999999000	23.2300000000000000	16.0749999999999000
H	26.8270000000000000	25.5539999999999000	15.4879999999999000
H	27.5779999999999000	27.1110000000000000	16.8990000000000000
H	29.4929999999999000	25.6439999999999000	16.2590000000000000
H	28.7349999999999000	22.3090000000000000	14.7010000000000000
H	30.1580000000000000	24.8430000000000000	9.2270000000000000
H	29.8389999999999000	23.3339999999999000	10.0790000000000000
H	29.7010000000000000	28.1479999999999000	12.2949999999999000
H	27.7280000000000000	19.8629999999999000	14.3670000000000000
P	24.1409999999999000	24.0680000000000000	17.0360000000000000
O	23.0139999999999000	24.0219999999999000	18.0060000000000000
O	24.1469999999999000	22.8539999999999000	15.9570000000000000

O	24.1849999999999000	25.3930000000000000	16.0940000000000000
C	24.0509999999999000	26.6810000000000000	16.6660000000000000
C	24.2259999999999000	27.7600000000000000	15.5839999999999000
O	25.4810000000000000	27.6009999999999000	14.9280000000000000
C	23.2729999999999000	27.6679999999999000	14.4030000000000000
O	21.9660000000000000	28.1159999999999000	14.7390000000000000
C	23.9819999999999000	28.4909999999999000	13.3339999999999000
O	23.9490000000000000	29.8949999999999000	13.5269999999999000
C	25.4179999999999000	27.9969999999999000	13.5770000000000000
N	25.8829999999999000	26.9439999999999000	12.6730000000000000
C	26.2650000000000000	27.2959999999999000	11.3859999999999000
O	26.2989999999999000	28.4849999999999000	11.0679999999999000
N	26.5670000000000000	26.2340000000000000	10.5090000000000000
C	26.3359999999999000	25.0060000000000000	10.8070000000000000
N	26.5839999999999000	24.0320000000000000	9.9290000000000000
C	25.8249999999999000	24.6329999999999000	12.0869999999999000
C	25.6250000000000000	25.6230000000000000	12.9649999999999000
H	24.7880000000000000	26.8219999999999000	17.4570000000000000
H	23.0719999999999000	26.7809999999999000	17.1370000000000000
H	24.1799999999999000	28.7510000000000000	16.0380000000000000
H	23.1849999999999000	26.6250000000000000	14.0909999999999000
H	23.6490000000000000	28.2229999999999000	12.3330000000000000
H	23.0869999999999000	30.2210000000000000	13.2620000000000000
H	26.1630000000000000	28.7940000000000000	13.4870000000000000
H	26.3909999999999000	23.0680000000000000	10.1519999999999000
H	26.9619999999999000	24.2710000000000000	9.0199999999999900
H	25.6080000000000000	23.6099999999999000	12.3569999999999000
H	25.2390000000000000	25.4409999999999000	13.9529999999999000
H	23.4690000000000000	22.1670000000000000	16.1020000000000000
P	20.6649999999999000	27.7630000000000000	13.8109999999999000
O	19.3539999999999000	28.1739999999999000	14.3819999999999000
O	20.8090000000000000	26.1690000000000000	13.5419999999999000
O	21.0199999999999000	28.4119999999999000	12.3610000000000000
C	20.8659999999999000	29.8049999999999000	12.1609999999999000
C	21.3249999999999000	30.1969999999999000	10.7469999999999000
O	22.6380000000000000	29.7049999999999000	10.4939999999999000
C	20.5629999999999000	29.5470000000000000	9.6080000000000000
O	19.2719999999999000	30.1250000000000000	9.4489999999999900
C	21.5150000000000000	29.7300000000000000	8.4329999999999900
O	21.6140000000000000	31.0509999999999000	7.9260000000000000
C	22.8369999999999000	29.3900000000000000	9.1349999999999900
N	23.2310000000000000	27.9359999999999000	8.9789999999999900
C	23.0199999999999000	26.8810000000000000	9.7940000000000000
N	23.4480000000000000	25.7420000000000000	9.3030000000000000
C	23.9359999999999000	26.0710000000000000	8.0969999999999900
C	24.5249999999999000	25.2190000000000000	7.0839999999999900
O	24.7719999999999000	24.0159999999999000	7.1429999999999900
N	24.7899999999999000	25.9699999999999000	5.9660000000000000
C	24.5869999999999000	27.3270000000000000	5.7910000000000000

N	24.9250000000000000	27.8329999999999000	4.6020000000000000
N	24.1020000000000000	28.0880000000000000	6.7130000000000000
C	23.7920000000000000	27.4080000000000000	7.8540000000000000
H	21.4400000000000000	30.3569999999999000	12.9049999999999000
H	19.8240000000000000	30.0949999999999000	12.3040000000000000
H	21.3150000000000000	31.2830000000000000	10.6419999999999000
H	20.4149999999999000	28.4860000000000000	9.8279999999999900
H	21.3150000000000000	29.0189999999999000	7.6330000000000000
H	22.1380000000000000	31.0309999999999000	7.1210000000000000
H	23.6750000000000000	29.9849999999999000	8.7660000000000000
H	22.5330000000000000	27.0060000000000000	10.7490000000000000
H	25.1879999999999000	25.4579999999999000	5.1900000000000000
H	24.8129999999999000	28.8200000000000000	4.4269999999999900
H	25.2830000000000000	27.2409999999999000	3.8610000000000000
H	20.0330000000000000	25.6350000000000000	13.7980000000000000
P	18.1409999999999000	29.4840000000000000	8.4570000000000000
O	16.8000000000000000	30.1239999999999000	8.5299999999999900
O	18.1490000000000000	27.9070000000000000	8.8430000000000000
O	18.8329999999999000	29.4989999999999000	6.9859999999999900
C	18.8550000000000000	30.6879999999999000	6.2169999999999900
C	19.4579999999999000	30.3949999999999000	4.8350000000000000
O	20.7190000000000000	29.7439999999999000	4.9550000000000000
C	18.6879999999999000	29.3850000000000000	4.0010000000000000
O	17.5219999999999000	29.9860000000000000	3.4520000000000000
C	19.7109999999999000	28.9490000000000000	2.9630000000000000
O	19.9520000000000000	29.8880000000000000	1.9259999999999900
C	20.9559999999999000	28.8730000000000000	3.8690000000000000
N	21.3380000000000000	27.5320000000000000	4.3170000000000000
C	21.9540000000000000	26.6819999999999000	3.4089999999999900
O	22.2169999999999000	27.0949999999999000	2.2799999999999900
N	22.2210000000000000	25.3719999999999000	3.8559999999999900
C	21.7699999999999000	24.9200000000000000	4.9720000000000000
N	22.0120000000000000	23.6619999999999000	5.3449999999999900
C	21.0180000000000000	25.7480000000000000	5.8630000000000000
C	20.8359999999999000	27.0229999999999000	5.4930000000000000
H	19.4269999999999000	31.4609999999999000	6.7270000000000000
H	17.8449999999999000	31.0839999999999000	6.1010000000000000
H	19.5839999999999000	31.3279999999999000	4.2800000000000000
H	18.3889999999999000	28.5429999999999000	4.6299999999999900
H	19.4680000000000000	27.9699999999999000	2.5520000000000000
H	19.2070000000000000	29.8730000000000000	1.3210000000000000
H	21.8539999999999000	29.2680000000000000	3.3860000000000000
H	21.6950000000000000	23.3180000000000000	6.2380000000000000
H	22.5530000000000000	23.0549999999999000	4.7409999999999900
H	20.6110000000000000	25.3870000000000000	6.7960000000000000
H	20.2820000000000000	27.7240000000000000	6.0960000000000000
H	17.2820000000000000	27.5380000000000000	9.0969999999999900
P	16.2100000000000000	29.1039999999999000	3.0430000000000000
O	15.0289999999999000	29.8979999999999000	2.6110000000000000

O	15.9520000000000000	28.1729999999999000	4.3470000000000000
O	16.7789999999999000	28.0509999999999000	1.9379999999999900
C	16.9669999999999000	28.4869999999999000	0.6030000000000000
C	17.5549999999999000	27.3679999999999000	-0.2690000000000000
O	18.7830000000000000	26.9020000000000000	0.2800000000000000
C	16.7680000000000000	26.0689999999999000	-0.3150000000000000
O	15.5839999999999000	26.1870000000000000	-1.0980000000000000
C	17.8049999999999000	25.0899999999999000	-0.8490000000000000
O	18.1329999999999000	25.2300000000000000	-2.2220000000000000
C	19.0189999999999000	25.5459999999999000	-0.0240000000000000
N	19.1980000000000000	24.7429999999999000	1.2460000000000000
C	18.7899999999999000	25.0139999999999000	2.5030000000000000
N	19.0390000000000000	24.0530000000000000	3.3559999999999900
C	19.6149999999999000	23.0919999999999000	2.6150000000000000
C	20.0820000000000000	21.7989999999999000	2.9399999999999900
N	20.0279999999999000	21.2689999999999000	4.2249999999999900
N	20.5859999999999000	21.0219999999999000	1.9450000000000000
C	20.6129999999999000	21.5289999999999000	0.7050000000000000
N	20.2130000000000000	22.7349999999999000	0.2760000000000000
C	19.7220000000000000	23.4840000000000000	1.2929999999999900
H	17.6290000000000000	29.3530000000000000	0.5850000000000000
H	16.0169999999999000	28.8180000000000000	0.1830000000000000
H	17.7310000000000000	27.7469999999999000	-1.2769999999999900
H	16.4579999999999000	25.7989999999999000	0.6970000000000000
H	17.5479999999999000	24.0599999999999000	-0.6100000000000000
H	18.6969999999999000	24.4959999999999000	-2.4790000000000000
H	19.9570000000000000	25.4710000000000000	-0.5780000000000000
H	18.2959999999999000	25.9460000000000000	2.7360000000000000
H	20.3140000000000000	20.3129999999999000	4.3630000000000000
H	19.6030000000000000	21.8120000000000000	4.9619999999999900
H	21.0199999999999000	20.8750000000000000	-0.0530000000000000
H	15.0589999999999000	28.2429999999999000	4.7359999999999900
P	14.4090000000000000	25.0479999999999000	-1.0910000000000000
O	13.1730000000000000	25.4140000000000000	-1.8350000000000000
O	14.1750000000000000	24.7500000000000000	0.4870000000000000
O	15.1440000000000000	23.6920000000000000	-1.6120000000000000
C	15.4459999999999000	23.5369999999999000	-2.9870000000000000
C	16.2010000000000000	22.2169999999999000	-3.2109999999999900
O	17.2600000000000000	22.0749999999999000	-2.2709999999999900
C	15.3940000000000000	20.9600000000000000	-2.9409999999999900
O	14.5359999999999000	20.6909999999999000	-4.0460000000000000
C	16.4770000000000000	19.9089999999999000	-2.7400000000000000
O	17.1099999999999000	19.4699999999999000	-3.9329999999999900
C	17.4950000000000000	20.7289999999999000	-1.9330000000000000
N	17.3979999999999000	20.5339999999999000	-0.4310000000000000
C	16.8670000000000000	21.3290000000000000	0.5220000000000000
N	16.9929999999999000	20.8520000000000000	1.7360000000000000
C	17.5990000000000000	19.6670000000000000	1.5660000000000000
C	18.0300000000000000	18.6799999999999000	2.4840000000000000

N	17.9130000000000000	18.8069999999999000	3.8650000000000000
N	18.5869999999999000	17.5440000000000000	1.9920000000000000
C	18.7139999999999000	17.4259999999999000	0.6640000000000000
N	18.3859999999999000	18.3000000000000000	-0.2970000000000000
C	17.8299999999999000	19.4200000000000000	0.2260000000000000
H	16.0440000000000000	24.3760000000000000	-3.3370000000000000
H	14.5320000000000000	23.5509999999999000	-3.5819999999999900
H	16.6080000000000000	22.1909999999999000	-4.2240000000000000
H	14.7910000000000000	21.0889999999999000	-2.0390000000000000
H	16.1149999999999000	19.0670000000000000	-2.1520000000000000
H	16.5030000000000000	18.9039999999999000	-4.4139999999999900
H	18.5259999999999000	20.4899999999999000	-2.2020000000000000
H	16.4130000000000000	22.2740000000000000	0.2650000000000000
H	18.2469999999999000	18.0560000000000000	4.4539999999999900
H	17.4649999999999000	19.6260000000000000	4.2500000000000000
H	19.1600000000000000	16.5060000000000000	0.3190000000000000
H	13.2539999999999000	24.8430000000000000	0.7950000000000000
P	13.2040000000000000	19.7540000000000000	-3.9180000000000000
O	12.4309999999999000	19.5790000000000000	-5.1779999999999900
O	12.3729999999999000	20.4439999999999000	-2.7050000000000000
O	13.7460000000000000	18.3730000000000000	-3.2500000000000000
C	14.3590000000000000	17.3850000000000000	-4.0570000000000000
C	14.7699999999999000	16.1810000000000000	-3.1970000000000000
O	15.6300000000000000	16.5850000000000000	-2.1379999999999900
C	13.6479999999999000	15.4990000000000000	-2.4340000000000000
O	12.9659999999999000	14.5869999999999000	-3.2829999999999900
C	14.3719999999999000	14.7820000000000000	-1.3080000000000000
O	15.0169999999999000	13.5809999999999000	-1.7070000000000000
C	15.4269999999999000	15.8480000000000000	-0.9570000000000000
N	15.0099999999999000	16.7940000000000000	0.1550000000000000
C	14.3140000000000000	17.9480000000000000	0.0720000000000000
N	14.0890000000000000	18.5199999999999000	1.2290000000000000
C	14.6470000000000000	17.6810000000000000	2.1139999999999900
C	14.6999999999999000	17.7899999999999000	3.5560000000000000
O	14.2460000000000000	18.6759999999999000	4.2759999999999900
N	15.3789999999999000	16.7100000000000000	4.0599999999999900
C	15.9179999999999000	15.6470000000000000	3.3620000000000000
N	16.4860000000000000	14.6920000000000000	4.1010000000000000
N	15.8710000000000000	15.5579999999999000	2.0750000000000000
C	15.2219999999999000	16.6060000000000000	1.4910000000000000
H	15.2300000000000000	17.7940000000000000	-4.5650000000000000
H	13.6739999999999000	17.0590000000000000	-4.8419999999999900
H	15.3040000000000000	15.4540000000000000	-3.8119999999999900
H	12.9469999999999000	16.2139999999999000	-1.9960000000000000
H	13.7110000000000000	14.6020000000000000	-0.4580000000000000
H	14.3450000000000000	12.9280000000000000	-1.9199999999999900
H	16.3810000000000000	15.4060000000000000	-0.6620000000000000
H	13.9930000000000000	18.3350000000000000	-0.8800000000000000
H	15.4849999999999000	16.7049999999999000	5.0659999999999900

H	16.8539999999999000	13.8610000000000000	3.6560000000000000
H	16.5740000000000000	14.7880000000000000	5.1050000000000000
H	12.8550000000000000	20.5609999999999000	-1.8650000000000000
P	11.4600000000000000	14.8650000000000000	-3.8460000000000000
O	11.1809999999999000	14.3190000000000000	-5.2020000000000000
O	11.2390000000000000	16.4680000000000000	-3.6949999999999900
O	10.5389999999999000	14.2439999999999000	-2.6589999999999900
C	10.5709999999999000	12.8439999999999000	-2.4399999999999900
C	9.6192895406047200	12.4376455455122000	-1.3224342002683600
O	10.0825100978849000	12.8935439739928000	-0.0486522752087800
C	8.1830470118368300	12.9507124052886000	-1.4616580170221200
O	7.3092555379096000	11.9653685659250000	-0.9222286436644200
C	8.1531481419947300	14.1690786644515000	-0.5151013747647400
O	6.8988716168337900	14.4884685616237000	-0.0086805476152900
C	9.0822715052070500	13.6372940629685000	0.6017100657151300
N	9.6923135388860800	14.6648877408301000	1.4107241632529200
C	9.3082692768586700	14.7666624781503000	2.7357728887673200
O	8.4816836966977800	13.9914214748312000	3.2486933423545400
N	9.8895696317441600	15.7864068364984000	3.4316025368136400
C	10.8611579033712000	16.6702664502571000	2.9659844504295400
O	11.3688777017092000	17.4833582672067000	3.7426550045141900
C	11.1804574651061000	16.5247243920255000	1.5610636932456500
C	10.6071488890435000	15.5316274583472000	0.8514714359240700
H	11.5809999999999000	12.5269999999999000	-2.1720000000000000
H	10.3119999999999000	12.3179999999999000	-3.3610000000000000
H	9.6135459517807000	11.3424165165297000	-1.3165415644245400
H	7.9146122026682900	13.1832364140087000	-2.4911543673894200
H	8.6264464513694800	15.0199243342550000	-1.0203217936731900
H	6.4038677887597300	15.0027088155230000	-0.6690234060110600
H	8.4815262945438400	13.0003221420072000	1.2770805864552100
H	9.7166472352874300	15.8047779980418000	4.4476592829571800
H	11.9073149557864000	17.2093461082011000	1.1411722561429400
H	10.8368342653452000	15.3353211796751000	-0.1900142100907900
H	11.0210000000000000	16.9409999999999000	-4.5220000000000000
P	6.4699256517454300	11.0326808520633000	-1.8831843036529200
O	5.8589708448506100	9.9308899091905200	-1.1249279954301100
O	7.4734912653838600	10.6538586078399000	-3.0309938491756600
O	5.4313782409034300	12.0046265003126000	-2.6299113963257800
C	4.4304383243140100	12.5747917269798000	-1.7893960992372300
C	3.7120000000000000	13.6969999999999000	-2.5520000000000000
O	2.7850000000000000	13.1560000000000000	-3.4880000000000000
C	2.8700000000000000	14.5679999999999000	-1.6110000000000000
O	2.9310000000000000	15.9339999999999000	-2.0129999999999900
C	1.4540000000000000	14.0399999999999000	-1.7749999999999900
O	0.3970000000000000	14.9369999999999000	-1.4900000000000000
C	1.4910000000000000	13.6180000000000000	-3.2460000000000000
N	0.5140000000000000	12.6300000000000000	-3.6880000000000000
C	-0.2950000000000000	12.8970000000000000	-4.7770000000000000
O	-0.2950000000000000	13.9209999999999000	-5.4600000000000000

N	-1.1690000000000000	11.8859999999999000	-5.0789999999999900
C	-1.3350000000000000	10.6669999999999000	-4.4909999999999900
O	-2.1819999999999900	9.8599999999999900	-4.8650000000000000
C	-0.4120000000000000	10.4380000000000000	-3.3969999999999900
C	0.4560000000000000	11.4090000000000000	-3.0539999999999900
H	4.9083540888028300	12.9833984597322000	-0.8965523673002200
H	3.7274345962430700	11.7913922803502000	-1.4970676680668900
H	4.4600000000000000	14.2870000000000000	-3.0790000000000000
H	3.1669999999999900	14.5009999999999000	-0.5639999999999990
H	1.3450000000000000	13.1539999999999000	-1.1490000000000000
H	0.7020000000000000	15.8309999999999000	-1.6650000000000000
H	1.3790000000000000	14.5099999999999000	-3.8690000000000000
H	-1.7829999999999900	12.0730000000000000	-5.8559999999999900
H	-0.4230000000000000	9.4900000000000000	-2.8790000000000000
H	1.1790000000000000	11.2820000000000000	-2.2610000000000000
H	7.0925569039670200	10.0977633931094000	-3.7163564205928100
P	4.1079999999999900	16.9349999999999000	-1.4750000000000000
O	3.8990000000000000	18.3750000000000000	-1.7840000000000000
O	5.4660000000000000	16.3109999999999000	-2.1080000000000000
O	4.2389999999999900	16.5740000000000000	0.1080000000000000
C	3.2380000000000000	16.9980000000000000	1.0169999999999900
C	3.6207734638489100	16.5589793264112000	2.4328884676003500
O	3.5784841268247200	15.1366576121951000	2.5112439064382700
C	5.0276233141814100	16.9954379756815000	2.9261535622139400
O	4.8980714361079200	17.6998727858598000	4.1479010174184000
C	5.7550343459589800	15.6525149547215000	3.1765332740298200
O	6.6336774692392200	15.6845668009515000	4.2546177754502500
C	4.5401850636801500	14.7420405994472000	3.4643748326370200
N	4.8040000000000000	13.3270000000000000	3.3759999999999900
C	4.4649999999999900	12.5229999999999000	4.4500000000000000
O	4.0570000000000000	12.8970000000000000	5.5469999999999900
N	4.5979999999999900	11.1809999999999000	4.2080000000000000
C	4.9619999999999900	10.5320000000000000	3.0659999999999900
O	4.9720000000000000	9.3079999999999900	2.9710000000000000
C	5.3060000000000000	11.4380000000000000	1.9870000000000000
C	5.2060000000000000	12.7650000000000000	2.1859999999999900
H	2.2730000000000000	16.5650000000000000	0.7480000000000000
H	3.1240000000000000	18.0809999999999000	0.9600000000000000
H	2.8826730038997400	16.9823856816968000	3.1278153830709500
H	5.5376821061946200	17.6226760292676000	2.1913555970086200
H	6.2458635117087100	15.3367480610520000	2.2502982248057800
H	7.3708877440261600	15.0514553499646000	4.0745682752526900
H	4.1831331965391000	14.9340312567916000	4.4889726353268800
H	4.3650000000000000	10.5809999999999000	4.9850000000000000
H	5.6219999999999900	11.0370000000000000	1.0360000000000000
H	5.4169999999999900	13.4809999999999000	1.4099999999999900
H	5.9340000000000000	16.8999999999999000	-2.7309999999999900
P	5.9974556032339100	18.8567522009312000	4.4428141854627600
O	5.8648747069910500	19.9994333283904000	3.5267087052969400

O	7.4073450653529200	18.2017655585041000	4.4801515780598000
O	5.7174808802994300	19.1826255217235000	5.9770610418516100
C	4.6524859090632100	20.0390717518053000	6.3558145175150900
C	4.2889999999999900	19.7190000000000000	7.8040000000000000
O	3.8670000000000000	18.3619999999999000	7.8929999999999900
C	5.4509999999999900	19.7480000000000000	8.7840000000000000
O	5.7260000000000000	21.0650000000000000	9.2460000000000000
C	4.9760000000000000	18.8520000000000000	9.9100000000000000
O	4.0190000000000000	19.4359999999999000	10.7799999999999000
C	4.3060000000000000	17.7340000000000000	9.0770000000000000
N	5.2293393571065900	16.6472992983020000	8.7855342889112500
C	5.7395371742885900	15.9166916008274000	9.8830622481436500
O	5.4902314984296100	16.3049630899961000	11.0421445216920000
N	6.4935512477015500	14.8208940627951000	9.6171449213883300
C	6.6294312652849800	14.3808228060487000	8.3534679866048600
N	7.3470236358709400	13.2748862883261000	8.1383031883276700
C	6.0727502526826000	15.0803935881339000	7.2416051835224300
C	5.3638664092553200	16.2000774117540000	7.5053715089472400
H	3.7850147346400700	19.8746023473625000	5.7153620075228000
H	4.9662638820844000	21.0837718037979000	6.2757944764823300
H	3.4700000000000000	20.3490000000000000	8.1579999999999900
H	6.3460000000000000	19.3129999999999000	8.3369999999999900
H	5.8259999999999900	18.4879999999999000	10.4770000000000000
H	4.4600000000000000	20.0889999999999000	11.3279999999999000
H	3.4100000000000000	17.3410000000000000	9.5660000000000000
H	7.3819726340087300	12.8842057249572000	7.2120050565774300
H	7.6933276944405500	12.7018055540277000	8.9009329410442400
H	6.1391920110773100	14.7068997903989000	6.2322905700883800
H	4.8680064133133500	16.8078089952446000	6.7555758708733300
H	7.3694058478638400	17.2259297058773000	4.6378691731471600
P	7.1859999999999900	21.7710000000000000	9.0389999999999900
O	7.2169999999999900	23.2319999999999000	9.3179999999999900
O	7.6230000000000000	21.3500000000000000	7.5320000000000000
O	8.1660000000000000	20.8769999999999000	9.9870000000000000
C	7.9930000000000000	20.9100000000000000	11.3970000000000000
C	8.5878589994972900	19.6196160919431000	11.9674933681276000
O	7.9513577754188400	18.5379095997466000	11.2703574877843000
C	10.0923626139893000	19.3350626217635000	11.7590210061794000
O	10.9317002768587000	19.9515984301850000	12.7089336608207000
C	10.1156990710458000	17.8007219333698000	11.8887790099302000
O	10.1918528565969000	17.4443543029200000	13.2373076800735000
C	8.7644950185852400	17.4116176871559000	11.2505871388668000
N	9.0255411058840500	16.9409684077511000	9.8942905308224800
C	9.5937979865397900	15.6755088401745000	9.8191474858719600
O	9.8952802960001800	15.0400562304310000	10.8391896526633000
N	9.8129161297429900	15.2267717456755000	8.5501860677307200
C	9.4994330764432100	15.8836238671044000	7.3727118028492900
O	9.6634736233538500	15.3144036226440000	6.2892043704990000
C	9.0127524544513000	17.2399840961672000	7.5422337771412600

C	8.7921442213031800	17.6999838980189000	8.7880696467977900
H	6.9329999999999900	20.9710000000000000	11.6470000000000000
H	8.4550000000000000	21.8129999999999000	11.7989999999999000
H	8.3657216710701900	19.5627494278192000	13.0411247467326000
H	10.3799382860648000	19.6354146838186000	10.7466910740858000
H	10.9359068845833000	17.3531559586911000	11.3206726190873000
H	10.2436906927743000	16.4786208490125000	13.2839386223480000
H	8.2846630458704500	16.5806590094668000	11.7974461229313000
H	10.2451895473812000	14.2868764183782000	8.4893191055898300
H	8.8855119047433600	17.8494673426087000	6.6555293031622500
H	8.4536706595175200	18.6967757644923000	9.0556046503212100
H	7.5910000000000000	20.3979999999999000	7.3209999999999900
P	12.5288244652981000	19.7341542197970000	12.6407926246992000
O	13.1769296629335000	20.5490384357122000	13.6667749602401000
O	12.9573222731344000	20.0322852787471000	11.1672738741832000
O	12.8725007913061000	18.1602943030655000	12.7719371710199000
C	13.1622813579295000	17.5559794677264000	14.0101709341422000
C	13.9797701783323000	16.2719202638578000	13.7978159505591000
O	13.2721221608839000	15.3641038558716000	12.9097157559949000
C	15.3378964730898000	16.4715263079262000	13.1246495268542000
O	16.3224154567269000	16.8402854052598000	14.1086166173300000
C	15.5929211533790000	15.0901367671245000	12.5093033194547000
O	15.9236527607679000	14.1438846075455000	13.5264919141440000
C	14.1873077839912000	14.7197906221282000	12.0222026542000000
N	13.9096006671782000	15.2147648138678000	10.6709526965989000
C	13.3279068273641000	16.4368386068881000	10.4069834501237000
N	13.1325500168170000	16.6569996677732000	9.1305038800678200
C	13.6064682560117000	15.5237188828089000	8.5069947706259400
C	13.6515446659487000	15.1244582794139000	7.1565971553488000
N	13.2190703354656000	15.8763788813853000	6.1435048833742700
N	14.1502419571826000	13.8923469390670000	6.8894397559344300
C	14.5886181544828000	13.1197732027477000	7.8892557197585100
N	14.6077494954445000	13.3907655963169000	9.1991706588466800
C	14.0935052038721000	14.6056728200857000	9.4449470312514300
H	12.2255518632366000	17.3053137234104000	14.5200797004712000
H	13.7373964585853000	18.2461758510891000	14.6380255644889000
H	14.1381019394010000	15.7817089705130000	14.7681387727260000
H	15.2814068126574000	17.2343348119694000	12.3463687166211000
H	16.3403336445417000	15.0770358572897000	11.7020608536252000
H	16.5444137556340000	14.5968671716581000	14.1201483464057000
H	14.0445026784319000	13.6325301725442000	12.0371315685606000
H	13.0587716173030000	17.0346481201641000	11.2666325829593000
H	13.1834466566583000	15.4959592387332000	5.2008197468345000
H	12.8581081060589000	16.8021702239551000	6.3005750881136000
H	14.9638928549578000	12.1397870227919000	7.5903986074798100
H	12.8576753805323000	19.3386850146950000	10.4952262089868000
P	17.4959783955255000	17.9000795442495000	13.7253143921384000
O	18.2734528183355000	18.2218270161891000	14.9487859262486000
O	16.8044630836950000	19.0626179461046000	12.8885527170500000

O	18.3062993459818000	17.2505700040559000	12.5115053717336000
C	19.2271554928676000	16.1746869104107000	12.8040396461691000
C	19.5650000000000000	15.5090000000000000	11.4800000000000000
O	18.3750000000000000	15.1880000000000000	10.7639999999999000
C	20.2880000000000000	16.3999999999999000	10.4760000000000000
O	21.6909999999999000	16.5139999999999000	10.6959999999999000
C	20.0000000000000000	15.7439999999999000	9.1349999999999900
O	20.8159999999999000	14.6129999999999000	8.8699999999999900
C	18.5380000000000000	15.3140000000000000	9.3710000000000000
N	17.5139999999999000	16.1980000000000000	8.8030000000000000
C	17.0189999999999000	15.9610000000000000	7.5270000000000000
O	17.3019999999999000	14.9120000000000000	6.9489999999999900
N	16.2079999999999000	16.9729999999999000	6.9699999999999900
C	15.9230000000000000	18.0590000000000000	7.5990000000000000
N	15.1669999999999000	19.0040000000000000	7.0359999999999900
C	16.4050000000000000	18.3039999999999000	8.9199999999999900
C	17.1849999999999000	17.3610000000000000	9.4589999999999900
H	18.7585031322850000	15.4224111349960000	13.4465705151271000
H	20.1189126575123000	16.5771644174159000	13.2979229306799000
H	20.1230000000000000	14.5860000000000000	11.6479999999999000
H	19.8619999999999000	17.4050000000000000	10.4499999999999000
H	20.0640000000000000	16.4619999999999000	8.3149999999999900
H	21.7070000000000000	14.9250000000000000	8.6929999999999900
H	18.3410000000000000	14.3170000000000000	8.9740000000000000
H	14.9440000000000000	19.8520000000000000	7.5350000000000000
H	14.8040000000000000	18.8730000000000000	6.0990000000000000
H	16.1679999999999000	19.1999999999999000	9.4749999999999900
H	17.5940000000000000	17.4549999999999000	10.4499999999999000
H	16.2309377713521000	19.6531134729608000	13.4056594993179000
P	22.3049999999999000	17.7710000000000000	11.5380000000000000
O	21.5519999999999000	19.0489999999999000	11.4090000000000000
O	23.8449999999999000	17.8369999999999000	11.0229999999999000
O	22.4280000000000000	17.1960000000000000	13.0570000000000000
C	23.3900000000000000	16.2139999999999000	13.4009999999999000
C	22.7920000000000000	15.2319999999999000	14.4190000000000000
O	21.9119999999999000	14.3279999999999000	13.7579999999999000
C	23.8069999999999000	14.2880000000000000	15.0410000000000000
O	24.5410000000000000	14.9390000000000000	16.0770000000000000
C	22.9669999999999000	13.0969999999999000	15.4789999999999000
O	22.2220000000000000	13.3019999999999000	16.6679999999999000
C	21.9869999999999000	13.0250000000000000	14.2889999999999000
N	22.2650000000000000	12.0220000000000000	13.2560000000000000
C	22.1999999999999000	10.6809999999999000	13.6099999999999000
O	21.8440000000000000	10.3729999999999000	14.7490000000000000
N	22.5670000000000000	9.7490000000000000	12.6210000000000000
C	23.0770000000000000	10.0990000000000000	11.4969999999999000
N	23.4209999999999000	9.1760000000000000	10.5980000000000000
C	23.2549999999999000	11.4779999999999000	11.1560000000000000
C	22.8359999999999000	12.3819999999999000	12.0540000000000000

H	24.2459999999999000	16.7349999999999000	13.8330000000000000
H	23.7469999999999000	15.6699999999999000	12.5229999999999000
H	22.2459999999999000	15.7660000000000000	15.1989999999999000
H	24.5300000000000000	13.9819999999999000	14.2810000000000000
H	23.5689999999999000	12.1929999999999000	15.5480000000000000
H	22.8219999999999000	13.2439999999999000	17.4149999999999000
H	20.9669999999999000	12.7799999999999000	14.6020000000000000
H	23.7710000000000000	9.4339999999999900	9.6850000000000000
H	23.2789999999999000	8.1969999999999900	10.8130000000000000
H	23.6960000000000000	11.7949999999999000	10.2219999999999000
H	22.9170000000000000	13.4450000000000000	11.8770000000000000
H	24.3479999999999000	17.0019999999999000	11.0749999999999000
P	25.7850000000000000	14.2279999999999000	16.8659999999999000
O	26.5010000000000000	15.0980000000000000	17.8380000000000000
O	26.7180000000000000	13.6750000000000000	15.6579999999999000
O	25.1479999999999000	12.8599999999999000	17.4789999999999000
C	24.7600000000000000	12.7490000000000000	18.8389999999999000
C	24.9310000000000000	11.2919999999999000	19.3030000000000000
O	24.2020000000000000	10.4239999999999000	18.4409999999999000
C	26.3359999999999000	10.7219999999999000	19.1789999999999000
O	27.1580000000000000	11.1720000000000000	20.2530000000000000
C	26.0859999999999000	9.2210000000000000	19.1499999999999000
O	25.7609999999999000	8.6449999999999900	20.4059999999999000
C	24.8399999999999000	9.1820000000000000	18.2549999999999000
N	25.1359999999999000	8.9800000000000000	16.7789999999999000
C	25.3810000000000000	9.8970000000000000	15.8179999999999000
N	25.5650000000000000	9.3849999999999900	14.6270000000000000
C	25.4819999999999000	8.0619999999999900	14.8320000000000000
C	25.5850000000000000	6.9870000000000000	13.8670000000000000
O	25.7330000000000000	7.0599999999999900	12.6489999999999000
N	25.4690000000000000	5.7880000000000000	14.5229999999999000
C	25.2680000000000000	5.5770000000000000	15.8770000000000000
N	25.1900000000000000	4.3040000000000000	16.2719999999999000
N	25.1529999999999000	6.5419999999999900	16.7270000000000000
C	25.2600000000000000	7.7740000000000000	16.1499999999999000
H	23.7229999999999000	13.0640000000000000	18.9490000000000000
H	25.3520000000000000	13.4019999999999000	19.4830000000000000
H	24.5590000000000000	11.1760000000000000	20.3219999999999000
H	26.7890000000000000	11.0589999999999000	18.2439999999999000
H	26.8990000000000000	8.6790000000000000	18.6759999999999000
H	26.5749999999999000	8.5069999999999900	20.8960000000000000
H	24.1430000000000000	8.3940000000000000	18.5479999999999000
H	25.3939999999999000	10.9489999999999000	16.0489999999999000
H	25.5270000000000000	4.9720000000000000	13.9269999999999000
H	25.0390000000000000	4.0910000000000000	17.2459999999999000
H	25.2920000000000000	3.5440000000000000	15.6099999999999000
H	27.6690000000000000	13.8770000000000000	15.7420000000000000
P	28.7869999999999000	11.0259999999999000	20.2429999999999000
O	29.4890000000000000	11.7430000000000000	21.3410000000000000

O	29.2040000000000000	11.5160000000000000	18.7530000000000000
O	29.0579999999999000	9.4220000000000000	20.1890000000000000
C	29.0210000000000000	8.6460000000000000	21.3730000000000000
C	29.1589999999999000	7.1509999999999900	21.0360000000000000
O	28.1900000000000000	6.7999999999999900	20.0500000000000000
C	30.4430000000000000	6.7229999999999900	20.3389999999999000
O	31.5489999999999000	6.6269999999999900	21.2330000000000000
C	30.0330000000000000	5.4080000000000000	19.6859999999999000
O	29.8590000000000000	4.3140000000000000	20.5730000000000000
C	28.6600000000000000	5.8230000000000000	19.1520000000000000
N	28.7160000000000000	6.3849999999999900	17.7429999999999000
C	28.9250000000000000	7.6520000000000000	17.3219999999999000
N	28.9690000000000000	7.7800000000000000	16.0199999999999000
C	28.8449999999999000	6.5240000000000000	15.5739999999999000
C	28.8719999999999000	6.0449999999999900	14.2110000000000000
O	28.9789999999999000	6.6980000000000000	13.1760000000000000
N	28.7450000000000000	4.6790000000000000	14.2159999999999000
C	28.6329999999999000	3.8410000000000000	15.3109999999999000
N	28.5899999999999000	2.5320000000000000	15.0500000000000000
N	28.5889999999999000	4.2779999999999900	16.5240000000000000
C	28.6980000000000000	5.6369999999999900	16.6039999999999000
H	28.0799999999999000	8.8200000000000000	21.8979999999999000
H	29.8170000000000000	8.9540000000000000	22.0530000000000000
H	28.9989999999999000	6.5510000000000000	21.9329999999999000
H	30.7130000000000000	7.4680000000000000	19.5859999999999000
H	30.6859999999999000	5.1360000000000000	18.8619999999999000
H	29.7349999999999000	3.5150000000000000	20.0530000000000000
H	27.9469999999999000	4.9950000000000000	19.1370000000000000
H	29.0420000000000000	8.4619999999999900	18.0210000000000000
H	28.7530000000000000	4.2549999999999900	13.2970000000000000
H	28.4959999999999000	1.8720000000000000	15.8059999999999000
H	28.6769999999999000	2.1890000000000000	14.1010000000000000
H	29.8780000000000000	12.2219999999999000	18.7210000000000000
P	33.0840000000000000	6.3979999999999900	20.7109999999999000
O	34.1300000000000000	6.4880000000000000	21.7659999999999000
O	33.2449999999999000	7.4900000000000000	19.5199999999999000
O	33.0480000000000000	4.9870000000000000	19.8990000000000000
C	33.0949999999999000	3.7540000000000000	20.5949999999999000
C	33.0769999999999000	2.5819999999999900	19.5979999999999000
O	31.9899999999999000	2.7210000000000000	18.6840000000000000
C	34.2610000000000000	2.5059999999999900	18.6449999999999000
O	35.3879999999999000	1.9190000000000000	19.2860000000000000
C	33.7130000000000000	1.6430000000000000	17.5169999999999000
O	33.6049999999999000	0.2600000000000000	17.8150000000000000
C	32.3059999999999000	2.2380000000000000	17.3979999999999000
N	32.2040000000000000	3.3599999999999900	16.3820000000000000
C	32.2670000000000000	4.6970000000000000	16.5659999999999000
N	32.1649999999999000	5.3910000000000000	15.4610000000000000
C	32.0889999999999000	4.4580000000000000	14.5039999999999000

C	32.0060000000000000	4.6379999999999900	13.0709999999999000
O	31.9149999999999000	5.6859999999999900	12.4390000000000000
N	32.0720000000000000	3.4119999999999900	12.4619999999999000
C	32.1659999999999000	2.1720000000000000	13.0649999999999000
N	32.2770000000000000	1.1299999999999900	12.2370000000000000
N	32.1619999999999000	2.0120000000000000	14.3460000000000000
C	32.1370000000000000	3.1960000000000000	15.0280000000000000
H	32.2509999999999000	3.6800000000000000	21.2789999999999000
H	33.9939999999999000	3.7010000000000000	21.2119999999999000
H	32.9690000000000000	1.6410000000000000	20.1389999999999000
H	34.5230000000000000	3.5059999999999900	18.2920000000000000
H	34.2640000000000000	1.7989999999999900	16.5930000000000000
H	34.4859999999999000	-0.1210000000000000	17.8350000000000000
H	31.5609999999999000	1.4910000000000000	17.1140000000000000
H	32.3950000000000000	5.1189999999999900	17.5489999999999000
H	32.0730000000000000	3.4399999999999900	11.4510000000000000
H	32.3380000000000000	0.1940000000000000	12.6129999999999000
H	32.3149999999999000	1.2640000000000000	11.2370000000000000
H	34.0020000000000000	8.0990000000000000	19.6140000000000000
P	36.9209999999999000	2.2509999999999900	18.8230000000000000
O	37.9799999999999000	1.6030000000000000	19.6419999999999000
O	36.9699999999999000	3.8730000000000000	18.8210000000000000
O	36.9519999999999000	1.8799999999999900	17.2379999999999000
C	37.0439999999999000	0.5310000000000000	16.8140000000000000
C	36.9620000000000000	0.4340000000000000	15.2799999999999000
O	35.7610000000000000	1.0409999999999900	14.8079999999999000
C	38.0170000000000000	1.2100000000000000	14.5009999999999000
O	39.2710000000000000	0.5330000000000000	14.5109999999999000
C	37.3780000000000000	1.3420000000000000	13.1229999999999000
O	37.3470000000000000	0.1580000000000000	12.3450000000000000
C	35.9419999999999000	1.6739999999999900	13.5609999999999000
N	35.5870000000000000	3.0960000000000000	13.5999999999999000
C	35.4579999999999000	3.7770000000000000	12.3979999999999000
O	35.5349999999999000	3.1499999999999900	11.3390000000000000
N	35.2460000000000000	5.1680000000000000	12.4809999999999000
C	35.2869999999999000	5.8099999999999900	13.5939999999999000
N	35.0619999999999000	7.1239999999999900	13.6359999999999000
C	35.5369999999999000	5.1379999999999900	14.8300000000000000
C	35.6689999999999000	3.8060000000000000	14.7759999999999000
H	36.2419999999999000	-0.0550000000000000	17.2630000000000000
H	37.9799999999999000	0.0940000000000000	17.1660000000000000
H	36.9660000000000000	-0.6140000000000000	14.9800000000000000
H	38.1610000000000000	2.1890000000000000	14.9610000000000000
H	37.8070000000000000	2.1619999999999900	12.5559999999999000
H	38.2190000000000000	0.0140000000000000	11.9700000000000000
H	35.1869999999999000	1.2330000000000000	12.9049999999999000
H	35.0559999999999000	7.6200000000000000	14.5139999999999000
H	34.8359999999999000	7.6200000000000000	12.7829999999999000
H	35.5989999999999000	5.6559999999999900	15.7759999999999000

H	35.8389999999999000	3.2170000000000000	15.6590000000000000
H	37.6629999999999000	4.2670000000000000	19.3840000000000000
P	40.6730000000000000	1.2700000000000000	14.1020000000000000
O	41.9020000000000000	0.4720000000000000	14.3640000000000000
O	40.6109999999999000	2.6819999999999900	14.8989999999999000
O	40.4680000000000000	1.7190000000000000	12.5510000000000000
C	40.5720000000000000	0.7630000000000000	11.5079999999999000
C	40.2559999999999000	1.4290000000000000	10.1590000000000000
O	39.0399999999999000	2.1600000000000000	10.2639999999999000
C	41.2349999999999000	2.5019999999999900	9.7080000000000000
O	42.3539999999999000	1.8930000000000000	9.0660000000000000
C	40.3950000000000000	3.3559999999999900	8.7669999999999900
O	40.1910000000000000	2.8010000000000000	7.4770000000000000
C	39.0539999999999000	3.3450000000000000	9.5079999999999900
N	38.8149999999999000	4.5439999999999900	10.4039999999999000
C	38.8789999999999000	4.6490000000000000	11.7479999999999000
N	38.5090000000000000	5.8159999999999900	12.2059999999999000
C	38.2599999999999000	6.5339999999999900	11.1010000000000000
C	37.8059999999999000	7.8579999999999900	10.9230000000000000
N	37.4290000000000000	8.6720000000000000	11.9819999999999000
N	37.7120000000000000	8.3480000000000000	9.6600000000000000
C	38.0290000000000000	7.5320000000000000	8.6460000000000000
N	38.4110000000000000	6.2460000000000000	8.6839999999999900
C	38.5020000000000000	5.7949999999999900	9.9600000000000000
H	39.8819999999999000	-0.0590000000000000	11.6899999999999000
H	41.5730000000000000	0.3280000000000000	11.4890000000000000
H	40.1490000000000000	0.6700000000000000	9.3819999999999900
H	41.5880000000000000	3.0800000000000000	10.5649999999999000
H	40.7719999999999000	4.3700000000000000	8.7020000000000000
H	39.7490000000000000	3.4550000000000000	6.9279999999999900
H	38.2060000000000000	3.3119999999999900	8.8209999999999900
H	39.1739999999999000	3.8119999999999900	12.3599999999999000
H	37.0469999999999000	9.5899999999999900	11.7880000000000000
H	37.4930000000000000	8.3170000000000000	12.9260000000000000
H	37.9350000000000000	7.9610000000000000	7.6600000000000000
H	41.3440000000000000	2.8380000000000000	15.5239999999999000
P	43.7689999999999000	2.6690000000000000	8.8040000000000000
O	44.7619999999999000	1.9119999999999900	7.9939999999999900
O	44.2649999999999000	3.0339999999999900	10.3059999999999000
O	43.3509999999999000	4.1239999999999900	8.2050000000000000
C	43.1480000000000000	4.3300000000000000	6.8179999999999900
C	42.7859999999999000	5.8060000000000000	6.5609999999999900
O	41.6559999999999000	6.1890000000000000	7.3399999999999900
C	43.8299999999999000	6.8140000000000000	7.0140000000000000
O	44.8939999999999000	6.8670000000000000	6.0670000000000000
C	43.0319999999999000	8.1039999999999900	7.1859999999999900
O	42.6880000000000000	8.7660000000000000	5.9809999999999900
C	41.7419999999999000	7.5220000000000000	7.7919999999999900
N	41.5970000000000000	7.6139999999999900	9.2460000000000000

C	41.2019999999999000	8.8160000000000000	9.8149999999999900
O	41.0469999999999000	9.8019999999999900	9.0939999999999900
N	41.0170000000000000	8.8109999999999900	11.2159999999999000
C	41.3750000000000000	7.8209999999999900	11.9550000000000000
N	41.1989999999999000	7.8430000000000000	13.2780000000000000
C	41.9519999999999000	6.6410000000000000	11.3919999999999000
C	42.0349999999999000	6.5930000000000000	10.0559999999999000
H	42.3619999999999000	3.6770000000000000	6.4459999999999900
H	44.0510000000000000	4.0690000000000000	6.2649999999999900
H	42.5510000000000000	5.9550000000000000	5.5049999999999900
H	44.2529999999999000	6.4980000000000000	7.9690000000000000
H	43.5129999999999000	8.7829999999999900	7.8849999999999900
H	42.3200000000000000	9.6270000000000000	6.2000000000000000
H	40.8419999999999000	8.0069999999999900	7.4000000000000000
H	41.4629999999999000	7.0549999999999900	13.8469999999999000
H	40.7599999999999000	8.6419999999999900	13.7189999999999000
H	42.2999999999999000	5.8150000000000000	11.9939999999999000
H	42.4440000000000000	5.7480000000000000	9.5389999999999900
H	43.6319999999999000	3.5320000000000000	10.8550000000000000
P	46.3269999999999000	7.5880000000000000	6.3789999999999900
O	47.3819999999999000	7.3680000000000000	5.3510000000000000
O	46.7100000000000000	7.0460000000000000	7.8609999999999900
O	45.9489999999999000	9.1449999999999900	6.6479999999999900
C	45.6700000000000000	10.0180000000000000	5.5720000000000000
C	45.2559999999999000	11.3930000000000000	6.1180000000000000
O	44.1589999999999000	11.2720000000000000	7.0199999999999900
C	46.2929999999999000	12.1140000000000000	6.9729999999999900
O	47.2120000000000000	12.8109999999999000	6.1429999999999900
C	45.4609999999999000	13.0879999999999000	7.7900000000000000
O	45.0949999999999000	14.2430000000000000	7.0599999999999900
C	44.2199999999999000	12.2189999999999000	8.0609999999999900
N	44.1689999999999000	11.5570000000000000	9.3670000000000000
C	43.5540000000000000	12.1799999999999000	10.4440000000000000
O	43.1689999999999000	13.3450000000000000	10.3300000000000000
N	43.4470000000000000	11.4139999999999000	11.6259999999999000
C	44.0439999999999000	10.2840000000000000	11.7720000000000000
N	43.9750000000000000	9.6050000000000000	12.9160000000000000
C	44.8029999999999000	9.6989999999999900	10.7159999999999000
C	44.8370000000000000	10.3710000000000000	9.5600000000000000
H	44.8800000000000000	9.6059999999999900	4.9450000000000000
H	46.5459999999999000	10.1170000000000000	4.9299999999999900
H	44.9470000000000000	12.0350000000000000	5.2900000000000000
H	46.8250000000000000	11.4350000000000000	7.6410000000000000
H	45.9420000000000000	13.3640000000000000	8.7270000000000000
H	44.5439999999999000	14.8100000000000000	7.6090000000000000
H	43.2950000000000000	12.7970000000000000	7.9809999999999900
H	44.4680000000000000	8.7330000000000000	13.0329999999999000
H	43.4269999999999000	9.9760000000000000	13.6839999999999000
H	45.3400000000000000	8.7680000000000000	10.8219999999999000

H	45.3900000000000000	10.0180000000000000	8.7100000000000000
H	47.5900000000000000	6.6280000000000000	7.9340000000000000
P	48.7909999999999000	12.4190000000000000	6.0629999999999900
O	49.5039999999999000	12.9489999999999000	4.8700000000000000
O	48.8130000000000000	10.8010000000000000	6.2130000000000000
O	49.3499999999999000	12.9410000000000000	7.5019999999999900
C	50.7400000000000000	13.1470000000000000	7.6829999999999900
C	50.9650000000000000	13.9770000000000000	8.9570000000000000
O	50.6289999999999000	13.1829999999999000	10.0909999999999000
C	52.4249999999999000	14.3469999999999000	9.2200000000000000
O	52.4159999999999000	15.4710000000000000	10.0920000000000000
C	52.9969999999999000	13.1460000000000000	9.9529999999999900
O	54.1169999999999000	13.4130000000000000	10.7780000000000000
C	51.7689999999999000	12.7159999999999000	10.7530000000000000
N	51.6520000000000000	11.2460000000000000	11.0990000000000000
C	51.9420000000000000	10.1709999999999000	10.3339999999999000
N	51.7800000000000000	9.0250000000000000	10.9510000000000000
C	51.3659999999999000	9.3719999999999900	12.1769999999999000
C	51.0379999999999000	8.5190000000000000	13.3019999999999000
O	51.0810000000000000	7.2930000000000000	13.3750000000000000
N	50.6319999999999000	9.3010000000000000	14.3539999999999000
C	50.5390000000000000	10.6809999999999000	14.3940000000000000
N	50.0709999999999000	11.2010000000000000	15.5280000000000000
N	50.8619999999999000	11.4369999999999000	13.4000000000000000
C	51.2659999999999000	10.7319999999999000	12.3030000000000000
H	51.1409999999999000	13.6929999999999000	6.8259999999999900
H	51.2490000000000000	12.1829999999999000	7.7320000000000000
H	50.3380000000000000	14.8699999999999000	8.9179999999999900
H	52.9779999999999000	14.5730000000000000	8.3070000000000000
H	53.2449999999999000	12.3699999999999000	9.2289999999999900
H	54.1180000000000000	14.3499999999999000	10.9949999999999000
H	51.7680000000000000	13.2520000000000000	11.7040000000000000
H	52.2779999999999000	10.2859999999999000	9.3140000000000000
H	50.3599999999999000	8.7899999999999900	15.1790000000000000
H	49.9279999999999000	12.1999999999999000	15.5939999999999000
H	49.7280000000000000	10.6069999999999000	16.2689999999999000
H	49.2430000000000000	10.3200000000000000	5.4809999999999900
P	53.1390000000000000	16.8739999999999000	9.6890000000000000
O	53.5159999999999000	17.0470000000000000	8.2590000000000000
O	54.3800000000000000	16.9379999999999000	10.7309999999999000
O	52.1129999999999000	17.9860000000000000	10.2810000000000000
C	51.1009999999999000	18.5450000000000000	9.4619999999999900
C	50.0660000000000000	19.2639999999999000	10.3450000000000000
O	49.2959999999999000	18.2800000000000000	11.0280000000000000
C	50.6820000000000000	20.1129999999999000	11.4640000000000000
O	49.9229999999999000	21.3069999999999000	11.6400000000000000
C	50.5989999999999000	19.2310000000000000	12.7040000000000000
O	50.6089999999999000	19.9009999999999000	13.9510000000000000
C	49.2989999999999000	18.4810000000000000	12.4130000000000000

N	49.0849999999999000	17.2289999999999000	13.1310000000000000
C	48.6129999999999000	17.2940000000000000	14.4280000000000000
O	48.2589999999999000	18.3090000000000000	15.0280000000000000
N	48.5540000000000000	16.0829999999999000	15.0619999999999000
C	48.9440000000000000	14.8510000000000000	14.6319999999999000
O	48.8489999999999000	13.8520000000000000	15.3399999999999000
C	49.4750000000000000	14.8650000000000000	13.2820000000000000
C	49.5309999999999000	16.0309999999999000	12.6080000000000000
H	50.6109999999999000	17.7789999999999000	8.8559999999999900
H	51.5579999999999000	19.2530000000000000	8.7699999999999900
H	49.3969999999999000	19.8479999999999000	9.7149999999999900
H	51.7209999999999000	20.3880000000000000	11.2780000000000000
H	51.4099999999999000	18.5010000000000000	12.6869999999999000
H	51.4829999999999000	20.2699999999999000	14.0960000000000000
H	48.4209999999999000	19.0979999999999000	12.6259999999999000
H	48.1820000000000000	16.1090000000000000	16.0000000000000000
H	49.8139999999999000	13.9450000000000000	12.8339999999999000
H	49.9200000000000000	16.0919999999999000	11.6010000000000000
H	55.2429999999999000	17.1720000000000000	10.3369999999999000
P	50.3810000000000000	22.6870000000000000	10.8960000000000000
O	51.8489999999999000	22.9310000000000000	10.8559999999999000
O	49.5319999999999000	23.8270000000000000	11.6799999999999000
O	49.6540000000000000	22.5940000000000000	9.4380000000000000
C	48.2499999999999000	22.7480000000000000	9.3439999999999900
C	47.7219999999999000	22.2210000000000000	7.9989999999999900
O	47.8639999999999000	20.8069999999999000	7.9649999999999900
C	46.2219999999999000	22.4649999999999000	7.8209999999999900
O	46.0360000000000000	23.6600000000000000	7.0629999999999900
C	45.6540000000000000	21.1879999999999000	7.2039999999999900
O	45.7409999999999000	21.1239999999999000	5.7910000000000000
C	46.6370000000000000	20.1700000000000000	7.7980000000000000
N	46.2520000000000000	19.4439999999999000	9.0760000000000000
C	45.6180000000000000	19.9130000000000000	10.1709999999999000
N	45.4789999999999000	19.0249999999999000	11.1229999999999000
C	46.0309999999999000	17.9100000000000000	10.6210000000000000
C	46.1799999999999000	16.6090000000000000	11.1519999999999000
N	45.7109999999999000	16.2379999999999000	12.4079999999999000
N	46.7959999999999000	15.6639999999999000	10.3930000000000000
C	47.2329999999999000	16.0249999999999000	9.1790000000000000
N	47.1490000000000000	17.2149999999999000	8.5679999999999900
C	46.5249999999999000	18.1320000000000000	9.3480000000000000
H	48.0200000000000000	23.8109999999999000	9.4410000000000000
H	47.7560000000000000	22.2280000000000000	10.1669999999999000
H	48.2869999999999000	22.6439999999999000	7.1669999999999900
H	45.7649999999999000	22.6340000000000000	8.7970000000000000
H	44.6420000000000000	20.9819999999999000	7.5460000000000000
H	45.0050000000000000	21.6179999999999000	5.4199999999999900
H	46.8170000000000000	19.3780000000000000	7.0690000000000000
H	45.2809999999999000	20.9370000000000000	10.2260000000000000

H	45.8659999999999000	15.2889999999999000	12.7230000000000000
H	45.3689999999999000	16.9520000000000000	13.0389999999999000
H	47.7190000000000000	15.2449999999999000	8.6099999999999900
H	50.0649999999999000	24.5440000000000000	12.0719999999999000
P	44.6069999999999000	24.4480000000000000	6.9669999999999900
O	44.6610000000000000	25.7699999999999000	6.2889999999999900
O	44.1030000000000000	24.5049999999999000	8.5090000000000000
O	43.5970000000000000	23.3659999999999000	6.2949999999999900
C	43.4560000000000000	23.2800000000000000	4.8869999999999900
C	42.3440000000000000	22.2719999999999000	4.5419999999999900
O	42.6179999999999000	21.0519999999999000	5.2350000000000000
C	40.9380000000000000	22.6539999999999000	5.0229999999999900
O	40.0009999999999000	21.9710000000000000	4.1959999999999900
C	40.8419999999999000	22.0410000000000000	6.4080000000000000
O	39.5260000000000000	21.8520000000000000	6.9020000000000000
C	41.5720000000000000	20.7330000000000000	6.1090000000000000
N	42.0309999999999000	19.9080000000000000	7.2850000000000000
C	42.1779999999999000	20.2579999999999000	8.5779999999999900
N	42.5200000000000000	19.2600000000000000	9.3499999999999900
C	42.6420000000000000	18.2169999999999000	8.5199999999999900
C	42.9390000000000000	16.8569999999999000	8.7520000000000000
N	43.1549999999999000	16.3219999999999000	10.0150000000000000
N	42.9819999999999000	16.0189999999999000	7.6879999999999900
C	42.7610000000000000	16.5309999999999000	6.4699999999999900
N	42.4540000000000000	17.7890000000000000	6.1260000000000000
C	42.3950000000000000	18.5949999999999000	7.2140000000000000
H	44.3999999999999000	22.9710000000000000	4.4390000000000000
H	43.2180000000000000	24.2560000000000000	4.4610000000000000
H	42.3440000000000000	22.1039999999999000	3.4640000000000000
H	40.7509999999999000	23.7300000000000000	5.0090000000000000
H	41.4229999999999000	22.6209999999999000	7.1230000000000000
H	39.5750000000000000	21.6239999999999000	7.8339999999999900
H	40.9119999999999000	20.0659999999999000	5.5519999999999900
H	41.9799999999999000	21.2650000000000000	8.9149999999999900
H	43.2909999999999000	15.3239999999999000	10.1039999999999000
H	43.0630000000000000	16.9170000000000000	10.8300000000000000
H	42.8109999999999000	15.8239999999999000	5.6529999999999900
H	43.8800000000000000	25.3969999999999000	8.8390000000000000
P	38.8509999999999000	22.7650000000000000	3.3660000000000000
O	39.3389999999999000	23.5139999999999000	2.1760000000000000
O	38.1300000000000000	23.6720000000000000	4.5039999999999900
O	37.7530000000000000	21.5990000000000000	3.0569999999999900
C	37.2060000000000000	20.7830000000000000	4.0869999999999900
C	37.6079999999999000	19.3150000000000000	3.8530000000000000
O	38.8689999999999000	19.0120000000000000	4.4550000000000000
C	36.7559999999999000	18.2469999999999000	4.5250000000000000
O	35.4870000000000000	18.0229999999999000	3.9260000000000000
C	37.6439999999999000	17.0219999999999000	4.3929999999999900
O	37.7800000000000000	16.4890000000000000	3.0850000000000000

C	38.9579999999999000	17.6559999999999000	4.8510000000000000
N	39.1450000000000000	17.5459999999999000	6.3559999999999900
C	38.9169999999999000	18.4639999999999000	7.3170000000000000
N	39.1009999999999000	18.0159999999999000	8.5340000000000000
C	39.4139999999999000	16.7220000000000000	8.3640000000000000
C	39.6869999999999000	15.6850000000000000	9.2870000000000000
N	39.6920000000000000	15.8680000000000000	10.6649999999999000
N	39.9500000000000000	14.4429999999999000	8.8030000000000000
C	39.9399999999999000	14.2669999999999000	7.4760000000000000
N	39.6980000000000000	15.1630000000000000	6.5069999999999900
C	39.4450000000000000	16.3919999999999000	7.0199999999999900
H	36.1250000000000000	20.8850000000000000	4.0069999999999900
H	37.4889999999999000	21.1149999999999000	5.0869999999999900
H	37.6579999999999000	19.1239999999999000	2.7789999999999900
H	36.6090000000000000	18.4909999999999000	5.5810000000000000
H	37.3509999999999000	16.2379999999999000	5.0850000000000000
H	36.9709999999999000	16.0219999999999000	2.8590000000000000
H	39.8299999999999000	17.1960000000000000	4.3830000000000000
H	38.6019999999999000	19.4619999999999000	7.0549999999999900
H	39.8629999999999000	15.0730000000000000	11.2660000000000000
H	39.4969999999999000	16.7850000000000000	11.0459999999999000
H	40.1510000000000000	13.2629999999999000	7.1390000000000000
H	37.8969999999999000	23.2040000000000000	5.3289999999999900
P	34.1139999999999000	18.2680000000000000	4.7770000000000000
O	32.8509999999999000	18.0749999999999000	4.0149999999999900
O	34.3070000000000000	19.7570000000000000	5.3929999999999900
O	34.3040000000000000	17.3090000000000000	6.0860000000000000
C	33.7920000000000000	15.9849999999999000	6.1079999999999900
C	34.7589999999999000	14.9830000000000000	5.4500000000000000
O	35.9040000000000000	14.7769999999999000	6.2699999999999900
C	34.2070000000000000	13.5719999999999000	5.3200000000000000
O	33.3719999999999000	13.5169999999999000	4.1689999999999900
C	35.4579999999999000	12.7080000000000000	5.2309999999999900
O	36.1210000000000000	12.7409999999999000	3.9760000000000000
C	36.3290000000000000	13.4339999999999000	6.2779999999999900
N	36.3430000000000000	12.8949999999999000	7.6379999999999900
C	36.9590000000000000	11.6730000000000000	7.8440000000000000
O	37.5709999999999000	11.0210000000000000	6.9980000000000000
N	36.8290000000000000	11.1910000000000000	9.1199999999999900
C	36.1480000000000000	11.7029999999999000	10.1850000000000000
O	36.1330000000000000	11.1739999999999000	11.2929999999999000
C	35.4569999999999000	12.9350000000000000	9.8680000000000000
C	35.5810000000000000	13.4640000000000000	8.6379999999999900
H	32.8190000000000000	15.9550000000000000	5.6139999999999900
H	33.6199999999999000	15.7100000000000000	7.1479999999999900
H	35.0810000000000000	15.3469999999999000	4.4729999999999900
H	33.6270000000000000	13.3230000000000000	6.2130000000000000
H	35.2659999999999000	11.6809999999999000	5.5300000000000000
H	35.6300000000000000	12.1950000000000000	3.3570000000000000

H	37.3870000000000000	13.4469999999999000	5.9980000000000000
H	37.2939999999999000	10.3100000000000000	9.2929999999999900
H	34.8439999999999000	13.4109999999999000	10.6159999999999000
H	35.0840000000000000	14.3840000000000000	8.3750000000000000
H	33.5640000000000000	20.3709999999999000	5.2320000000000000
P	32.0820000000000000	12.5220000000000000	4.0629999999999900
O	31.1619999999999000	12.7989999999999000	2.9279999999999900
O	31.3900000000000000	12.6150000000000000	5.5289999999999900
O	32.7490000000000000	11.0399999999999000	4.0599999999999900
C	33.4149999999999000	10.5779999999999000	2.8969999999999900
C	34.0409999999999000	9.2029999999999900	3.1640000000000000
O	34.9239999999999000	9.2989999999999900	4.2729999999999900
C	33.0739999999999000	8.1240000000000000	3.6230000000000000
O	32.3969999999999000	7.5720000000000000	2.4980000000000000
C	33.9859999999999000	7.1449999999999900	4.3499999999999900
O	34.7659999999999000	6.3120000000000000	3.5070000000000000
C	34.9260000000000000	8.1349999999999900	5.0579999999999900
N	34.5330000000000000	8.4730000000000000	6.4829999999999900
C	33.9449999999999000	9.5790000000000000	6.9880000000000000
N	33.7899999999999000	9.5489999999999900	8.2889999999999900
C	34.2520000000000000	8.3399999999999900	8.6410000000000000
C	34.3230000000000000	7.7370000000000000	9.9559999999999900
O	34.0539999999999000	8.2360000000000000	11.0459999999999000
N	34.7640000000000000	6.4429999999999900	9.8279999999999900
C	35.1379999999999000	5.7850000000000000	8.6690000000000000
N	35.5060000000000000	4.5099999999999900	8.7980000000000000
N	35.1379999999999000	6.3540000000000000	7.5110000000000000
C	34.6779999999999000	7.6369999999999900	7.5490000000000000
H	34.1880000000000000	11.2859999999999000	2.6010000000000000
H	32.7130000000000000	10.5139999999999000	2.0619999999999900
H	34.6049999999999000	8.8689999999999900	2.2909999999999900
H	32.3340000000000000	8.5470000000000000	4.3070000000000000
H	33.4429999999999000	6.5549999999999900	5.0830000000000000
H	35.2150000000000000	5.6600000000000000	4.0510000000000000
H	35.9510000000000000	7.7619999999999900	5.1090000000000000
H	33.6619999999999000	10.4000000000000000	6.3470000000000000
H	34.8329999999999000	5.9329999999999900	10.6999999999999000
H	35.7909999999999000	3.9849999999999900	7.9859999999999900
H	35.4939999999999000	4.0499999999999900	9.7020000000000000
H	30.4340000000000000	12.8130000000000000	5.5259999999999900
P	30.9869999999999000	6.7560000000000000	2.6280000000000000
O	30.3769999999999000	6.3330000000000000	1.3380000000000000
O	30.0560000000000000	7.7500000000000000	3.5120000000000000
O	31.3099999999999000	5.5460000000000000	3.6650000000000000
C	31.9909999999999000	4.3780000000000000	3.2429999999999900
C	32.1809999999999000	3.4340000000000000	4.4429999999999900
O	32.8530000000000000	4.1059999999999900	5.5060000000000000
C	30.8949999999999000	2.9910000000000000	5.1230000000000000
O	30.2650000000000000	1.9690000000000000	4.3559999999999900

C	31.3539999999999000	2.5830000000000000	6.5170000000000000
O	32.0150000000000000	1.3310000000000000	6.5940000000000000
C	32.3890000000000000	3.6940000000000000	6.7709999999999900
N	31.9409999999999000	4.8289999999999900	7.5780000000000000
C	31.8679999999999000	4.6559999999999900	8.9469999999999900
O	32.1749999999999000	3.6339999999999900	9.5619999999999900
N	31.4089999999999000	5.7569999999999900	9.6189999999999900
C	30.9549999999999000	6.9520000000000000	9.1430000000000000
O	30.5919999999999000	7.8689999999999900	9.8740000000000000
C	30.9520000000000000	7.0129999999999900	7.6959999999999900
C	31.4289999999999000	5.9669999999999900	6.9960000000000000
H	32.9540000000000000	4.6360000000000000	2.8060000000000000
H	31.4220000000000000	3.8750000000000000	2.4590000000000000
H	32.7730000000000000	2.5659999999999900	4.1459999999999900
H	30.2049999999999000	3.8350000000000000	5.1810000000000000
H	30.5470000000000000	2.6429999999999900	7.2409999999999900
H	31.3509999999999000	0.6390000000000000	6.6310000000000000
H	33.2760000000000000	3.3230000000000000	7.2939999999999900
H	31.4089999999999000	5.6810000000000000	10.6280000000000000
H	30.5700000000000000	7.8910000000000000	7.1980000000000000
H	31.4420000000000000	5.9690000000000000	5.9230000000000000
H	29.2300000000000000	8.0380000000000000	3.0790000000000000
P	28.7190000000000000	1.4960000000000000	4.6029999999999900
O	28.1550000000000000	0.6100000000000000	3.5510000000000000
O	27.9190000000000000	2.8940000000000000	4.8060000000000000
O	28.7270000000000000	0.8620000000000000	6.0999999999999900
C	29.2160000000000000	-0.4470000000000000	6.3250000000000000
C	29.1969999999999000	-0.7460000000000000	7.8330000000000000
O	29.8919999999999000	0.2750000000000000	8.5399999999999900
C	27.8270000000000000	-0.7070000000000000	8.4979999999999900
O	27.1400000000000000	-1.9299999999999900	8.2509999999999900
C	28.1709999999999000	-0.4920000000000000	9.9649999999999900
O	28.6200000000000000	-1.6459999999999900	10.6579999999999000
C	29.3339999999999000	0.5010000000000000	9.8119999999999900
N	29.0049999999999000	1.9139999999999900	10.0069999999999000
C	28.9570000000000000	2.4300000000000000	11.2919999999999000
O	29.0530000000000000	1.6670000000000000	12.2550000000000000
N	28.7579999999999000	3.8199999999999900	11.3910000000000000
C	28.3659999999999000	4.5330000000000000	10.3949999999999000
N	28.1200000000000000	5.8350000000000000	10.5389999999999000
C	28.1999999999999000	3.9630000000000000	9.0950000000000000
C	28.5309999999999000	2.6720000000000000	8.9610000000000000
H	30.2289999999999000	-0.5420000000000000	5.9349999999999900
H	28.6060000000000000	-1.1759999999999900	5.7889999999999900
H	29.6829999999999000	-1.7050000000000000	8.0280000000000000
H	27.2480000000000000	0.1340000000000000	8.1129999999999900
H	27.3539999999999000	-0.0180000000000000	10.4960000000000000
H	27.8560000000000000	-2.1840000000000000	10.8789999999999000
H	30.1449999999999000	0.2980000000000000	10.5190000000000000

H	27.8170000000000000	6.4009999999999900	9.7609999999999900
H	28.2600000000000000	6.2720000000000000	11.4429999999999000
H	27.8339999999999000	4.5259999999999900	8.2490000000000000
H	28.4450000000000000	2.1480000000000000	8.0250000000000000
H	27.1709999999999000	3.0350000000000000	4.1950000000000000
P	25.5279999999999000	-2.0099999999999900	7.9889999999999900
O	25.0689999999999000	-3.2460000000000000	7.2990000000000000
O	25.1720000000000000	-0.6310000000000000	7.2069999999999900
O	24.8889999999999000	-1.7809999999999900	9.4689999999999900
C	25.0139999999999000	-2.7980000000000000	10.4499999999999000
C	24.8949999999999000	-2.2180000000000000	11.8680000000000000
O	25.8440000000000000	-1.1679999999999900	12.0280000000000000
C	23.6000000000000000	-1.4930000000000000	12.2059999999999000
O	22.5369999999999000	-2.4060000000000000	12.4589999999999000
C	24.0010000000000000	-0.6250000000000000	13.3989999999999000
O	24.1769999999999000	-1.3180000000000000	14.6240000000000000
C	25.3870000000000000	-0.1730000000000000	12.9120000000000000
N	25.4450000000000000	1.1619999999999900	12.3160000000000000
C	25.4780000000000000	2.2669999999999900	13.1539999999999000
O	25.4810000000000000	2.0990000000000000	14.3750000000000000
N	25.4639999999999000	3.5280000000000000	12.5250000000000000
C	25.1879999999999000	3.6669999999999900	11.2769999999999000
N	25.0820000000000000	4.8780000000000000	10.7270000000000000
C	25.0010000000000000	2.5369999999999900	10.4220000000000000
C	25.1430000000000000	1.3300000000000000	10.9830000000000000
H	25.9819999999999000	-3.2930000000000000	10.3559999999999000
H	24.2579999999999000	-3.5659999999999900	10.2850000000000000
H	25.0930000000000000	-2.9980000000000000	12.6059999999999000
H	23.2959999999999000	-0.8760000000000000	11.3580000000000000
H	23.3430000000000000	0.2320000000000000	13.5229999999999000
H	24.3309999999999000	-0.6740000000000000	15.3200000000000000
H	26.1350000000000000	-0.1560000000000000	13.7110000000000000
H	24.8249999999999000	4.9859999999999900	9.7579999999999900
H	25.2270000000000000	5.7000000000000000	11.2989999999999000
H	24.7639999999999000	2.6290000000000000	9.3719999999999900
H	25.0320000000000000	0.4180000000000000	10.4199999999999000
H	24.7149999999999000	-0.7460000000000000	6.3510000000000000
P	20.9810000000000000	-1.9159999999999900	12.5820000000000000
O	19.9869999999999000	-3.0019999999999900	12.7980000000000000
O	20.7500000000000000	-1.0629999999999900	11.2200000000000000
O	21.0060000000000000	-0.7610000000000000	13.7279999999999000
C	20.9499999999999000	-1.1030000000000000	15.1020000000000000
C	20.8629999999999000	0.1820000000000000	15.9420000000000000
O	21.9280000000000000	1.0700000000000000	15.6280000000000000
C	19.6589999999999000	1.0460000000000000	15.6289999999999000
O	18.5109999999999000	0.4740000000000000	16.2420000000000000
C	20.0599999999999000	2.4190000000000000	16.1430000000000000
O	19.9589999999999000	2.5750000000000000	17.5489999999999000
C	21.5459999999999000	2.4220000000000000	15.7319999999999000

N	21.8120000000000000	3.1280000000000000	14.4179999999999000
C	21.7880000000000000	2.6469999999999900	13.1549999999999000
N	21.9879999999999000	3.5609999999999900	12.2360000000000000
C	22.1200000000000000	4.6980000000000000	12.9350000000000000
C	22.2910000000000000	6.0529999999999900	12.4499999999999000
O	22.4220000000000000	6.4459999999999900	11.2919999999999000
N	22.2639999999999000	6.9160000000000000	13.5160000000000000
C	22.1149999999999000	6.6020000000000000	14.8559999999999000
N	22.0730000000000000	7.6299999999999900	15.7070000000000000
N	22.0079999999999000	5.3910000000000000	15.2829999999999000
C	22.0040000000000000	4.4699999999999900	14.2769999999999000
H	21.8159999999999000	-1.6940000000000000	15.3900000000000000
H	20.0779999999999000	-1.7290000000000000	15.3000000000000000
H	20.8999999999999000	-0.0590000000000000	17.0070000000000000
H	19.4890000000000000	1.0730000000000000	14.5510000000000000
H	19.5169999999999000	3.2130000000000000	15.6280000000000000
H	19.0289999999999000	2.6450000000000000	17.7820000000000000
H	22.1829999999999000	2.8950000000000000	16.4830000000000000
H	21.6000000000000000	1.6010000000000000	12.9649999999999000
H	22.3530000000000000	7.8940000000000000	13.2739999999999000
H	21.9750000000000000	7.4569999999999900	16.6960000000000000
H	22.0859999999999000	8.5869999999999900	15.3729999999999000
H	19.9969999999999000	-1.3530000000000000	10.6730000000000000
P	16.9939999999999000	0.9640000000000000	15.9019999999999000
O	15.9239999999999000	0.1130000000000000	16.4860000000000000
O	16.9750000000000000	1.0540000000000000	14.2810000000000000
O	16.9570000000000000	2.5249999999999900	16.3619999999999000
C	15.7149999999999000	3.1989999999999900	16.2689999999999000
C	15.8249999999999000	4.6570000000000000	16.7280000000000000
O	16.6140000000000000	5.4020000000000000	15.8089999999999000
C	14.5000000000000000	5.3940000000000000	16.6589999999999000
O	13.7330000000000000	5.1090000000000000	17.8249999999999000
C	14.9199999999999000	6.8460000000000000	16.5289999999999000
O	15.3309999999999000	7.4660000000000000	17.7379999999999000
C	16.1250000000000000	6.7030000000000000	15.5969999999999000
N	15.7754172635686000	6.8429339044553400	14.2041858534973000
C	15.5513554235977000	5.8185215422061800	13.3019756524432000
N	15.4008335213665000	6.2335235787090300	12.0676989064840000
C	15.5401514865558000	7.6000778977230600	12.1536886070073000
C	15.5340009884238000	8.6169975131274700	11.1860022499484000
N	15.2943860709310000	8.3514204885427600	9.8956072304692200
N	15.7787654671476000	9.8901677836993200	11.5750062995092000
C	15.9571771509670000	10.1190734746521000	12.8888427240931000
N	15.9552719348001000	9.2578883831420800	13.9189054915155000
C	15.7751982768646000	8.0026909114706400	13.4677402558975000
H	14.9920000000000000	2.6800000000000000	16.9020000000000000
H	15.3450000000000000	3.1490000000000000	15.2430000000000000
H	16.2620000000000000	4.7260000000000000	17.7259999999999000
H	13.9450000000000000	5.0759999999999900	15.7729999999999000

H	14.1570000000000000	7.4240000000000000	16.0259999999999000
H	15.4520000000000000	8.4049999999999900	17.5749999999999000
H	16.9130000000000000	7.4139999999999900	15.8469999999999000
H	15.6582627262823000	4.7944783730169900	13.5938200191503000
H	15.1092281020025000	9.1074616396973500	9.2585505972832600
H	14.9505028055408000	7.4333088070933700	9.6723237067234800
H	16.1033851143201000	11.1666700369327000	13.1524917936746000
H	16.3350000000000000	0.4610000000000000	13.8399999999999000
P	12.0980000000000000	5.1619999999999900	17.8410000000000000
O	11.4629999999999000	4.6369999999999900	19.0790000000000000
O	11.6750000000000000	4.3760000000000000	16.4849999999999000
O	11.7449999999999000	6.7110000000000000	17.4920000000000000
C	11.9580000000000000	7.7309999999999900	18.4510000000000000
C	11.9407500162455000	9.0953642389659700	17.7381171429706000
O	12.9284349155117000	9.0774945960170200	16.6719553342931000
C	10.6607192700711000	9.4792072743659900	16.9977265306007000
O	9.6863748014673000	9.9844547353791500	17.9270839873328000
C	11.1704764350782000	10.5236205789037000	15.9933077462927000
O	11.3226502761689000	11.7530262890285000	16.6998371244290000
C	12.5372656681165000	9.9348430102491000	15.6055105189545000
N	12.4599443643757000	9.1769745738066300	14.3384921419024000
C	12.5283108936835000	9.9040421077034300	13.1323379370612000
O	12.6387445995703000	11.1642882989695000	13.2076543970885000
N	12.4431322445109000	9.2437117978262400	11.9673288104126000
C	12.2513810997355000	7.9015880672154200	11.9582209720734000
N	12.1639980576985000	7.2847678232962500	10.7904625227940000
C	12.0993146521870000	7.1579473344363400	13.1694050088001000
C	12.2073064413705000	7.8300744694105400	14.3333606880380000
H	12.9350000000000000	7.6130000000000000	18.9200000000000000
H	11.2180000000000000	7.6500000000000000	19.2489999999999000
H	12.1830037333735000	9.8856644528202200	18.4630879673105000
H	10.2659953207692000	8.5992154120161300	16.4829329610271000
H	10.5395691868747000	10.6630540844819000	15.1088096924972000
H	11.7113821106939000	12.3761887679406000	16.0710722668339000
H	13.2834392393270000	10.7213087307263000	15.4469588695262000
H	12.0037873496215000	6.2934607166960700	10.7842443757679000
H	12.2974882662854000	7.7640620739064000	9.9045654685754500
H	11.8998829165967000	6.1000374746058000	13.1692487189284000
H	12.0865171942051000	7.3865791334095600	15.3168630329411000
H	11.0510000000000000	3.6360000000000000	16.6140000000000000
P	8.1361647985123700	10.0188121136993000	17.4865539935817000
O	7.3042280710025600	10.6406000829883000	18.5512925047492000
O	7.8936214866981800	8.5076448118657500	17.0779690429152000
O	8.0596666106737400	10.7562584115002000	16.0545107178314000
C	8.0628251344147400	12.1989106521499000	15.9952077071736000
C	7.8666552354124900	12.6590266761452000	14.5741348688112000
O	8.9441442587495100	12.1916688084576000	13.7286140389128000
C	6.6198462283245600	12.1714840636913000	13.8452067446600000
O	5.4609213083554900	12.8460032178675000	14.3605466215316000

C	6.9944015115119500	12.4777924571899000	12.3931637481949000
O	6.9349856323226300	13.8829429546794000	12.2145423405967000
C	8.4606714891415100	12.0028456782962000	12.3995455198543000
N	8.5998969698268900	10.5769046138762000	12.0246250346937000
C	8.6286865954882400	10.2837220966412000	10.6706964124926000
O	8.5337377467435700	11.1627333876999000	9.7944415281250800
N	8.7683118403565700	8.9682665677618600	10.3583589559004000
C	8.7296360938739100	7.8841478764606900	11.2466720181908000
O	8.7454562825324300	6.7371477692703300	10.8149359663894000
C	8.6849200052917200	8.2678650472446400	12.6318872841501000
C	8.6275039217645600	9.5713042526988300	12.9692032064714000
H	9.0209322675018000	12.5677842575310000	16.3799342591470000
H	7.2475475527021100	12.5962349452375000	16.6112223541476000
H	7.8475942203778100	13.7597852964634000	14.5886610850781000
H	6.5086827217582300	11.0943611855036000	13.9920678505138000
H	6.3857898447159700	11.9565243257563000	11.6441285262918000
H	6.8618393379076500	14.0727973049369000	11.2458017892111000
H	9.0514412712184700	12.5842127412823000	11.6881194318821000
H	8.8032769868249900	8.7430105085963700	9.3519595783201100
H	8.6791841363700700	7.4990037364237200	13.3881347364284000
H	8.5945251202484800	9.9236518445080700	13.9937682247398000
H	7.0547651904281600	8.3559288073495400	16.6057649816087000
P	4.0104724050753200	12.1661432607492000	14.1646784698410000
O	2.9864040524105400	12.8859243762700000	14.9644628734690000
O	4.2141543963015000	10.6110986596118000	14.4007050534403000
O	3.7802600885663500	12.1289517462520000	12.5742520537463000
C	3.4464522984352300	13.3710255547104000	11.9275913726180000
C	3.2010000000000000	13.0770000000000000	10.4580000000000000
O	4.3579999999999900	12.5090000000000000	9.8640000000000000
C	2.1680000000000000	12.0090000000000000	10.1539999999999000
O	0.8670000000000000	12.5579999999999000	10.3290000000000000
C	2.5129999999999900	11.6140000000000000	8.7249999999999900
O	2.1019999999999900	12.5530000000000000	7.7439999999999900
C	4.0490000000000000	11.6180000000000000	8.8239999999999900
N	4.6749999999999900	10.2720000000000000	9.1300000000000000
C	5.0019999999999900	9.7390000000000000	10.3260000000000000
N	5.5640000000000000	8.5600000000000000	10.2439999999999000
C	5.5659999999999900	8.2880000000000000	8.9309999999999900
C	6.0240000000000000	7.1660000000000000	8.2040000000000000
N	6.6600000000000000	6.0750000000000000	8.7899999999999900
N	5.8280000000000000	7.1479999999999900	6.8620000000000000
C	5.2300000000000000	8.2050000000000000	6.2960000000000000
N	4.7919999999999900	9.3369999999999900	6.8670000000000000
C	4.9880000000000000	9.3170000000000000	8.2070000000000000
H	4.2807486179421000	14.0807646239241000	12.0116652083975000
H	2.5413929807602300	13.7905348824792000	12.3810931834195000
H	2.9649999999999900	14.0120000000000000	9.9450000000000000
H	2.2940000000000000	11.1590000000000000	10.8279999999999000
H	2.1600000000000000	10.6150000000000000	8.4770000000000000

H	2.4220000000000000	12.2669999999999000	6.8840000000000000
H	4.5229999999999900	11.9809999999999000	7.9119999999999900
H	4.8179999999999900	10.2780000000000000	11.2420000000000000
H	7.0190000000000000	5.3430000000000000	8.1929999999999900
H	6.9770000000000000	6.1619999999999900	9.7449999999999900
H	5.0970000000000000	8.1370000000000000	5.2279999999999900
H	3.7482282670222400	10.2928704786465000	15.1918428470416000
P	-0.4200000000000000	11.6270000000000000	10.6989999999999000
O	-1.6650000000000000	12.3710000000000000	11.0310000000000000
O	0.1250000000000000	10.6660000000000000	11.8879999999999000
O	-0.5370000000000000	10.6180000000000000	9.4309999999999900
C	-1.0840000000000000	11.0809999999999000	8.2110000000000000
C	-1.0169999999999900	9.9730000000000000	7.1509999999999900
O	0.3390000000000000	9.5999999999999900	6.9249999999999900
C	-1.6220000000000000	8.6389999999999900	7.5599999999999900
O	-3.0449999999999900	8.6859999999999900	7.5410000000000000
C	-0.9720000000000000	7.6589999999999900	6.5890000000000000
O	-1.4790000000000000	7.6909999999999900	5.2640000000000000
C	0.4560000000000000	8.2379999999999900	6.5890000000000000
N	1.4170000000000000	7.5579999999999900	7.4520000000000000
C	2.0070000000000000	6.4059999999999900	6.9699999999999900
O	1.8839999999999900	5.9520000000000000	5.8330000000000000
N	2.7620000000000000	5.7409999999999900	7.8949999999999900
C	2.9600000000000000	5.9980000000000000	9.2180000000000000
O	3.6779999999999900	5.2980000000000000	9.9290000000000000
C	2.2519999999999900	7.1790000000000000	9.6709999999999900
C	1.5260000000000000	7.8890000000000000	8.7859999999999900
H	-0.5450000000000000	11.9629999999999000	7.8630000000000000
H	-2.1210000000000000	11.3870000000000000	8.3610000000000000
H	-1.4490000000000000	10.3249999999999000	6.2110000000000000
H	-1.3260000000000000	8.4060000000000000	8.5839999999999900
H	-0.9730000000000000	6.6440000000000000	6.9779999999999900
H	-1.1010000000000000	6.9550000000000000	4.7740000000000000
H	0.9090000000000000	8.2319999999999900	5.5919999999999900
H	3.2229999999999900	4.9100000000000000	7.5480000000000000
H	2.3159999999999900	7.4760000000000000	10.7070000000000000
H	0.9790000000000000	8.7699999999999900	9.0779999999999900
H	-0.3750000000000000	10.7189999999999000	12.7260000000000000
P	-3.9660000000000000	7.5419999999999900	8.2579999999999900
O	-5.4219999999999900	7.8479999999999900	8.3160000000000000
O	-3.2690000000000000	7.3399999999999900	9.7100000000000000
O	-3.5830000000000000	6.1680000000000000	7.4770000000000000
C	-4.1230000000000000	5.9139999999999900	6.1929999999999900
C	-3.5680000000000000	4.5880000000000000	5.6529999999999900
O	-2.1450000000000000	4.6040000000000000	5.6950000000000000
C	-3.8690000000000000	3.3639999999999900	6.4989999999999900
O	-5.2149999999999900	2.9359999999999900	6.3070000000000000
C	-2.7989999999999900	2.3769999999999900	6.0499999999999900
O	-3.0129999999999900	1.7909999999999900	4.7759999999999900

C	-1.6010000000000000	3.3319999999999900	5.9429999999999900
N	-0.7330000000000000	3.3690000000000000	7.1849999999999900
C	-0.7720000000000000	4.1929999999999900	8.2550000000000000
N	0.1370000000000000	3.9260000000000000	9.1609999999999900
C	0.7640000000000000	2.8420000000000000	8.6799999999999900
C	1.8410000000000000	2.0720000000000000	9.2669999999999900
O	2.4889999999999900	2.3069999999999900	10.2840000000000000
N	2.0590000000000000	0.9540000000000000	8.5009999999999900
C	1.4310000000000000	0.6010000000000000	7.3209999999999900
N	1.7909999999999900	-0.5649999999999990	6.7800000000000000
N	0.5360000000000000	1.3390000000000000	6.7569999999999900
C	0.2240000000000000	2.4480000000000000	7.4880000000000000
H	-3.8790000000000000	6.7290000000000000	5.5129999999999900
H	-5.2119999999999900	5.8689999999999900	6.2420000000000000
H	-3.8990000000000000	4.4329999999999900	4.6230000000000000
H	-3.7509999999999900	3.6090000000000000	7.5570000000000000
H	-2.6059999999999900	1.6130000000000000	6.8019999999999900
H	-2.3540000000000000	1.1050000000000000	4.6399999999999900
H	-0.9399999999999990	3.0790000000000000	5.1100000000000000
H	-1.4990000000000000	4.9880000000000000	8.3170000000000000
H	2.7650000000000000	0.3220000000000000	8.8580000000000000
H	1.3759999999999900	-0.8630000000000000	5.9100000000000000
H	2.4350000000000000	-1.1879999999999900	7.2539999999999900
H	-3.8630000000000000	7.4610000000000000	10.4760000000000000
P	-5.9230000000000000	1.8080000000000000	7.2539999999999900
O	-7.3689999999999900	1.5730000000000000	6.9880000000000000
O	-5.5979999999999900	2.3239999999999900	8.7590000000000000
O	-4.9720000000000000	0.4980000000000000	7.1090000000000000
C	-5.1260000000000000	-0.3970000000000000	6.0220000000000000
C	-4.2169999999999900	-1.6190000000000000	6.2320000000000000
O	-2.8679999999999900	-1.2100000000000000	6.4429999999999900
C	-4.4920000000000000	-2.4089999999999900	7.5010000000000000
O	-5.6589999999999900	-3.2080000000000000	7.3380000000000000
C	-3.1940000000000000	-3.1800000000000000	7.7210000000000000
O	-3.0040000000000000	-4.2990000000000000	6.8719999999999900
C	-2.1880000000000000	-2.0850000000000000	7.3170000000000000
N	-1.5569999999999900	-1.3600000000000000	8.4160000000000000
C	-0.5030000000000000	-1.9710000000000000	9.0690000000000000
O	-0.0070000000000000	-3.0600000000000000	8.7820000000000000
N	-0.0230000000000000	-1.2549999999999900	10.1310000000000000
C	-0.4630000000000000	-0.0840000000000000	10.6699999999999000
O	0.0920000000000000	0.4610000000000000	11.6199999999999000
C	-1.6390000000000000	0.4370000000000000	10.0030000000000000
C	-2.1269999999999900	-0.2160000000000000	8.9320000000000000
H	-4.8890000000000000	0.1000000000000000	5.0830000000000000
H	-6.1639999999999900	-0.7220000000000000	5.9420000000000000
H	-4.2610000000000000	-2.2740000000000000	5.3600000000000000
H	-4.6719999999999900	-1.7190000000000000	8.3279999999999900
H	-3.0619999999999900	-3.4560000000000000	8.7650000000000000

H	-2.2229999999999900	-4.7770000000000000	7.1650000000000000
H	-1.3540000000000000	-2.4800000000000000	6.7290000000000000
H	0.7700000000000000	-1.6750000000000000	10.5999999999999000
H	-2.1099999999999900	1.3360000000000000	10.3719999999999000
H	-3.0040000000000000	0.1240000000000000	8.4100000000000000
H	-6.3799999999999900	2.4700000000000000	9.3239999999999900
P	-6.4500000000000000	-3.8690000000000000	8.6050000000000000
O	-7.7240000000000000	-4.5590000000000000	8.2680000000000000
O	-6.6130000000000000	-2.6290000000000000	9.6400000000000000
O	-5.3319999999999900	-4.8010000000000000	9.3279999999999900
C	-5.0339999999999900	-6.0880000000000000	8.8170000000000000
C	-3.9870000000000000	-6.7640000000000000	9.7159999999999900
O	-2.8340000000000000	-5.9379999999999900	9.8390000000000000
C	-4.4009999999999900	-6.9180000000000000	11.1699999999999000
O	-5.2859999999999900	-8.0239999999999900	11.2929999999999000
C	-3.0760000000000000	-7.0910000000000000	11.8979999999999000
O	-2.5040000000000000	-8.3859999999999900	11.8100000000000000
C	-2.2109999999999900	-6.1020000000000000	11.0899999999999000
N	-1.9470000000000000	-4.8120000000000000	11.7279999999999000
C	-0.9560000000000000	-4.7320000000000000	12.6950000000000000
O	-0.3300000000000000	-5.7480000000000000	13.0050000000000000
N	-0.7550000000000000	-3.4670000000000000	13.2799999999999000
C	-1.5560000000000000	-2.4830000000000000	13.0839999999999000
N	-1.3550000000000000	-1.3069999999999900	13.6820000000000000
C	-2.6829999999999900	-2.5990000000000000	12.2089999999999000
C	-2.8270000000000000	-3.7669999999999900	11.5670000000000000
H	-4.6689999999999900	-6.0190000000000000	7.7930000000000000
H	-5.9370000000000000	-6.6989999999999900	8.7810000000000000
H	-3.6930000000000000	-7.7270000000000000	9.2949999999999900
H	-4.9070000000000000	-6.0119999999999900	11.5090000000000000
H	-3.1539999999999900	-6.7690000000000000	12.9350000000000000
H	-1.7320000000000000	-8.4179999999999900	12.3800000000000000
H	-1.2220000000000000	-6.5069999999999900	10.8520000000000000
H	-1.9740000000000000	-0.5290000000000000	13.5150000000000000
H	-0.5570000000000000	-1.1759999999999900	14.2910000000000000
H	-3.3919999999999900	-1.8000000000000000	12.0570000000000000
H	-3.6440000000000000	-3.9569999999999900	10.8900000000000000
H	-7.5309999999999900	-2.4279999999999900	9.9019999999999900
P	-6.4269999999999900	-8.0960000000000000	12.4580000000000000
O	-7.3769999999999900	-9.2340000000000000	12.3379999999999000
O	-7.1250000000000000	-6.6310000000000000	12.4060000000000000
O	-5.5690000000000000	-8.0540000000000000	13.8399999999999000
C	-4.8840000000000000	-9.2200000000000000	14.2669999999999000
C	-4.0129999999999900	-8.9209999999999900	15.4969999999999000
O	-3.0800000000000000	-7.8949999999999900	15.1880000000000000
C	-4.7370000000000000	-8.3369999999999900	16.7079999999999000
O	-5.4690000000000000	-9.3000000000000000	17.4460000000000000
C	-3.6070000000000000	-7.6639999999999900	17.4879999999999000
O	-2.8630000000000000	-8.5370000000000000	18.3270000000000000

C	-2.7120000000000000	-7.1689999999999900	16.3329999999999000
N	-2.7559999999999900	-5.7279999999999900	16.0749999999999000
C	-1.9470000000000000	-4.8849999999999900	16.8290000000000000
O	-1.1060000000000000	-5.3620000000000000	17.5919999999999000
N	-2.1829999999999900	-3.5019999999999900	16.6879999999999000
C	-3.1869999999999900	-3.0369999999999900	16.0369999999999000
N	-3.4460000000000000	-1.7270000000000000	16.0300000000000000
C	-4.0650000000000000	-3.8959999999999900	15.3000000000000000
C	-3.8029999999999900	-5.2089999999999900	15.3490000000000000
H	-4.2580000000000000	-9.6029999999999900	13.4600000000000000
H	-5.6010000000000000	-10.0069999999999000	14.5009999999999000
H	-3.4580000000000000	-9.8170000000000000	15.7829999999999000
H	-5.4299999999999900	-7.5620000000000000	16.3719999999999000
H	-4.8650000000000000	-9.9879999999999900	17.7349999999999000
H	-3.9830000000000000	-6.8200000000000000	18.0700000000000000
H	-3.3750000000000000	-8.7070000000000000	19.1219999999999000
H	-1.6570000000000000	-7.4160000000000000	16.4810000000000000
H	-4.2400000000000000	-1.3560000000000000	15.5320000000000000
H	-2.8340000000000000	-1.1000000000000000	16.5380000000000000
H	-4.8970000000000000	-3.5240000000000000	14.7210000000000000
H	-4.4050000000000000	-5.9400000000000000	14.8279999999999000
H	-8.0899999999999900	-6.6379999999999900	12.2609999999999000
N	10.7184634651119000	12.5083516569459000	6.0548788202338900
C	10.9354657032786000	11.5351797658504000	5.1100051289333200
N	11.5109585015228000	10.4591447265353000	5.6059529084249000
C	11.6784321572728000	10.7428881941059000	6.9559520528611400
C	12.1398171006391000	9.9443867018606700	8.0395588862839000
O	12.5511687200546000	8.7785965095345600	7.9955190066233700
N	12.0485365254762000	10.6330182726069000	9.2682426158260300
C	11.5895291593943000	11.9116221365931000	9.4399951772318000
N	11.5598545307291000	12.4086302437317000	10.6889012263103000
N	11.1542368636803000	12.6505244619560000	8.4239272032305100
C	11.1904913545757000	12.0134648113475000	7.2370672941422500
H	10.3283047791948000	13.4480641598931000	5.9437525034863600
H	10.5934984284489000	11.6373320541032000	4.0899550622497900
H	12.3399981604171000	10.1273080053817000	10.1212956319752000
H	11.1319291811353000	13.3172733511937000	10.8192791941633000
H	11.9644893681430000	11.9230645570421000	11.4900812311734000

S4.5 RNA-3F06

O	3.9120000000000	4.1480000000000	20.8919999999999
C	4.6840000000000	3.6790000000000	21.9819999999999
C	4.6190000000000	2.1480000000000	22.1600000000000
O	3.2820000000000	1.7220000000000	22.4349999999999
C	4.9390000000000	1.2970000000000	20.9370000000000
O	6.3000000000000	1.3930000000000	20.5090000000000
C	4.3230000000000	-0.0550000000000	21.3210000000000

O	4.9420000000000	-0.7930000000000	22.3619999999999
C	2.9860000000000	0.4630000000000	21.8689999999999
N	1.8910000000000	0.6080000000000	20.8240000000000
C	1.3710000000000	1.7380000000000	20.2970000000000
N	0.4470000000000	1.5240000000000	19.3930000000000
C	0.3720000000000	0.1870000000000	19.3150000000000
C	-0.4840000000000	-0.6250000000000	18.4740000000000
O	-1.3320000000000	-0.2620000000000	17.6600000000000
N	-0.2110000000000	-1.9490000000000	18.7049999999999
C	0.7110000000000	-2.4830000000000	19.5869999999999
N	0.7900000000000	-3.8160000000000	19.6099999999999
N	1.4610000000000	-1.7530000000000	20.3460000000000
C	1.2530000000000	-0.4130000000000	20.1739999999999
H	4.3540000000000	3.9070000000000	20.0749999999999
H	4.3440000000000	4.1710000000000	22.8939999999999
H	5.7180000000000	3.9910000000000	21.8369999999999
H	5.2550000000000	1.8590000000000	22.9989999999999
H	4.4040000000000	1.6870000000000	20.0689999999999
H	4.1490000000000	-0.6910000000000	20.4600000000000
H	5.7390000000000	-1.2020000000000	22.0169999999999
H	2.5780000000000	-0.1870000000000	22.6460000000000
H	1.7050000000000	2.7090000000000	20.6299999999999
H	-0.7580000000000	-2.5940000000000	18.1509999999999
H	1.4390000000000	-4.2710000000000	20.2330000000000
H	0.2140000000000	-4.3820000000000	18.9989999999999
P	7.5200000000000	0.4370000000000	21.0139999999999
O	7.7260000000000	0.3880000000000	22.4879999999999
O	8.7870000000000	0.9870000000000	20.1610000000000
O	7.1860000000000	-0.9820000000000	20.2890000000000
C	7.5550000000000	-2.2070000000000	20.8990000000000
C	6.9080000000000	-3.3790000000000	20.1469999999999
O	5.5030000000000	-3.1790000000000	20.0339999999999
C	7.2820000000000	-3.5030000000000	18.6829999999999
O	8.6030000000000	-4.0130000000000	18.5390000000000
C	6.1690000000000	-4.3870000000000	18.1310000000000
O	6.2620000000000	-5.7590000000000	18.4830000000000
C	4.9720000000000	-3.7840000000000	18.8769999999999
N	4.1990000000000	-2.7660000000000	18.0650000000000
C	4.3720000000000	-1.4320000000000	17.9549999999999
N	3.4890000000000	-0.8470000000000	17.1799999999999
C	2.7590000000000	-1.8650000000000	16.7020000000000
C	1.6410000000000	-1.8330000000000	15.7829999999999
O	1.0520000000000	-0.8560000000000	15.3260000000000
N	1.2910000000000	-3.1200000000000	15.4600000000000
C	1.8330000000000	-4.2930000000000	15.9529999999999
N	1.3540000000000	-5.4290000000000	15.4410000000000
N	2.7600000000000	-4.3120000000000	16.8509999999999
C	3.2010000000000	-3.0640000000000	17.1840000000000
H	7.2300000000000	-2.2260000000000	21.9400000000000

H	8.6410000000000	-2.3110000000000	20.9100000000000
H	7.1040000000000	-4.3140000000000	20.6750000000000
H	7.2640000000000	-2.5170000000000	18.2130000000000
H	6.0390000000000	-4.2680000000000	17.0569999999999
H	5.5860000000000	-6.2450000000000	18.0040000000000
H	4.2530000000000	-4.5420000000000	19.1969999999999
H	5.1600000000000	-0.9320000000000	18.4980000000000
H	0.5470000000000	-3.2040000000000	14.7780000000000
H	1.6990000000000	-6.3150000000000	15.7799999999999
H	0.6810000000000	-5.4210000000000	14.6850000000000
H	8.6520000000000	1.0470000000000	19.1969999999999
P	9.3490000000000	-4.0830000000000	17.0869999999999
O	10.7360000000000	-4.6210000000000	17.1069999999999
O	9.2330000000000	-2.5610000000000	16.5380000000000
O	8.2980000000000	-4.8860000000000	16.1419999999999
C	8.3530000000000	-6.2960000000000	16.0159999999999
C	7.5370000000000	-6.7100000000000	14.7840000000000
O	6.1870000000000	-6.2680000000000	14.8989999999999
C	7.9460000000000	-6.0040000000000	13.5079999999999
O	9.1870000000000	-6.5010000000000	13.0190000000000
C	6.7320000000000	-6.1710000000000	12.6090000000000
O	6.6070000000000	-7.4540000000000	12.0160000000000
C	5.6140000000000	-5.9710000000000	13.6449999999999
N	5.0550000000000	-4.5620000000000	13.6549999999999
C	5.5330000000000	-3.4640000000000	14.2780000000000
N	4.8520000000000	-2.3740000000000	14.0250000000000
C	3.9020000000000	-2.7720000000000	13.1649999999999
C	2.8710000000000	-2.0640000000000	12.5079999999999
N	2.6480000000000	-0.7020000000000	12.6850000000000
N	2.0630000000000	-2.7450000000000	11.6549999999999
C	2.2880000000000	-4.0550000000000	11.4840000000000
N	3.2190000000000	-4.8340000000000	12.0519999999999
C	4.0040000000000	-4.1270000000000	12.9009999999999
H	7.9770000000000	-6.7850000000000	16.9130000000000
H	9.3840000000000	-6.6330000000000	15.8930000000000
H	7.5550000000000	-7.7950000000000	14.6579999999999
H	8.1080000000000	-4.9430000000000	13.7089999999999
H	6.6860000000000	-5.3830000000000	11.8590000000000
H	7.2400000000000	-7.5170000000000	11.2970000000000
H	4.7730000000000	-6.6490000000000	13.4870000000000
H	6.4040000000000	-3.5230000000000	14.9120000000000
H	1.8430000000000	-0.2760000000000	12.2420000000000
H	3.1410000000000	-0.2240000000000	13.4250000000000
H	1.6230000000000	-4.5520000000000	10.7929999999999
H	10.0809999999999	-2.1470000000000	16.2880000000000
P	9.8910000000000	-5.8720000000000	11.6899999999999
O	11.2400000000000	-6.4070000000000	11.3670000000000
O	9.8490000000000	-4.2720000000000	11.9719999999999
O	8.7690000000000	-6.0930000000000	10.5350000000000

C	8.7820000000000	-5.2900000000000	9.3750000000000
C	7.4650000000000	-5.4890000000000	8.6140000000000
O	6.3360000000000	-5.1750000000000	9.4230000000000
C	7.3030000000000	-4.5300000000000	7.4510000000000
O	8.0060000000000	-5.0690000000000	6.3410000000000
C	5.7990000000000	-4.4320000000000	7.2580000000000
O	5.1930000000000	-5.5470000000000	6.6240000000000
C	5.3800000000000	-4.3970000000000	8.7430000000000
N	5.2050000000000	-3.0820000000000	9.3730000000000
C	4.1780000000000	-2.2600000000000	8.9290000000000
O	3.3890000000000	-2.6840000000000	8.0830000000000
N	4.1180000000000	-0.9670000000000	9.4880000000000
C	5.0170000000000	-0.5190000000000	10.2919999999999
N	4.9360000000000	0.7130000000000	10.8010000000000
C	6.1240000000000	-1.3250000000000	10.6969999999999
C	6.1670000000000	-2.5760000000000	10.2200000000000
H	9.6290000000000	-5.5800000000000	8.7510000000000
H	8.9140000000000	-4.2390000000000	9.6350000000000
H	7.3680000000000	-6.5250000000000	8.2830000000000
H	7.6990000000000	-3.5470000000000	7.7150000000000
H	5.5270000000000	-3.5090000000000	6.7470000000000
H	5.3990000000000	-5.5170000000000	5.6870000000000
H	4.4160000000000	-4.8860000000000	8.9160000000000
H	5.6290000000000	1.0450000000000	11.4540000000000
H	4.1490000000000	1.3060000000000	10.5679999999999
H	6.8920000000000	-0.9700000000000	11.3689999999999
H	6.9610000000000	-3.2540000000000	10.4920000000000
H	10.7219999999999	-3.8340000000000	11.9939999999999
P	8.9450000000000	-4.1400000000000	5.3850000000000
O	9.8730000000000	-4.8820000000000	4.4890000000000
O	9.6680000000000	-3.1020000000000	6.4050000000000
O	7.8410000000000	-3.2240000000000	4.6250000000000
C	6.9500000000000	-3.8510000000000	3.7200000000000
C	5.8890000000000	-2.8540000000000	3.2440000000000
O	5.0910000000000	-2.4160000000000	4.3340000000000
C	6.4440000000000	-1.5380000000000	2.7380000000000
O	6.9750000000000	-1.7250000000000	1.4320000000000
C	5.2410000000000	-0.6140000000000	2.8280000000000
O	4.2630000000000	-0.7950000000000	1.8160000000000
C	4.6640000000000	-1.0860000000000	4.1750000000000
N	5.1150000000000	-0.2680000000000	5.3670000000000
C	6.0700000000000	-0.5300000000000	6.2840000000000
N	6.1840000000000	0.3940000000000	7.2050000000000
C	5.2800000000000	1.3220000000000	6.8550000000000
C	4.9120000000000	2.5530000000000	7.4420000000000
N	5.4610000000000	3.0340000000000	8.6260000000000
N	3.9720000000000	3.3090000000000	6.8160000000000
C	3.4360000000000	2.8410000000000	5.6820000000000
N	3.6830000000000	1.6890000000000	5.0430000000000

C	4.6230000000000	0.9600000000000	5.6930000000000
H	6.4640000000000	-4.7090000000000	4.1840000000000
H	7.5090000000000	-4.2320000000000	2.8630000000000
H	5.2390000000000	-3.3210000000000	2.5020000000000
H	7.2380000000000	-1.1850000000000	3.3990000000000
H	5.5410000000000	0.4290000000000	2.8920000000000
H	3.6140000000000	-0.0900000000000	1.8870000000000
H	3.5720000000000	-1.0720000000000	4.1840000000000
H	6.6460000000000	-1.4420000000000	6.2370000000000
H	5.1000000000000	3.8930000000000	9.0200000000000
H	6.1060000000000	2.4510000000000	9.1390000000000
H	2.6930000000000	3.4750000000000	5.2210000000000
H	10.6379999999999	-3.0380000000000	6.3130000000000
P	8.1630000000000	-0.7750000000000	0.8390000000000
O	8.7400000000000	-1.2200000000000	-0.4580000000000
O	9.2340000000000	-0.6820000000000	2.0550000000000
O	7.4910000000000	0.7040000000000	0.8210000000000
C	6.5040000000000	1.0080000000000	-0.1470000000000
C	5.9310000000000	2.4070000000000	0.1110000000000
O	5.3580000000000	2.4830000000000	1.4110000000000
C	6.9610000000000	3.5220000000000	0.1790000000000
O	7.3930000000000	3.8660000000000	-1.1320000000000
C	6.2130000000000	4.6320000000000	0.9050000000000
O	5.2520000000000	5.3250000000000	0.1230000000000
C	5.4780000000000	3.7760000000000	1.9560000000000
N	6.1314767239090	3.7923452834340	3.2538241543969
C	5.7713453481661	4.8250555324447	4.1065118692319
O	4.9769221784458	5.6971578289213	3.7636416638782
N	6.3570050820001	4.8205375090727	5.3248108046137
C	7.3404307110647	3.9409557731500	5.7675571633169
O	7.8154404691505	4.0780283812410	6.8760348011043
C	7.6772980270791	2.9071853351167	4.8184437299060
C	7.0700962264446	2.8667379730034	3.6214528450313
H	5.7050000000000	0.2670000000000	-0.1260000000000
H	6.9410000000000	0.9640000000000	-1.1460000000000
H	5.1600000000000	2.6360000000000	-0.6280000000000
H	7.8220000000000	3.1970000000000	0.7660000000000
H	6.9010000000000	5.3240000000000	1.3870000000000
H	5.7080000000000	5.9450000000000	-0.4520000000000
H	4.4490000000000	4.1110000000000	2.1250000000000
H	6.1059013730668	5.6153303508329	5.9498749625855
H	8.4263432682922	2.1861281187356	5.0945626322176
H	7.2778477007637	2.1066663877981	2.8817851299359
H	10.1379999999999	-0.9760000000000	1.8340000000000
P	8.9000000000000	4.4220000000000	-1.4260000000000
O	9.2220000000000	4.6260000000000	-2.8630000000000
O	9.8690000000000	3.3720000000000	-0.6530000000000
O	8.9950000000000	5.7640000000000	-0.5100000000000
C	8.2890000000000	6.9120000000000	-0.9420000000000

C	8.3224559234171	8.0803033736267	0.0376230988799
O	7.7111995881536	7.6545881471254	1.2778286252631
C	9.7005487081474	8.5579410355668	0.4904810489043
O	10.3160335889391	9.4334520743585	-0.4591981762499
C	9.3555045733946	9.3275595210629	1.7842899391448
O	9.0007853166144	10.6745720151811	1.5196320320531
C	8.1005265100665	8.5757661368449	2.2897860492926
N	8.3783557250641	7.7816743410452	3.4718996941349
C	8.7321332304910	6.4450825420231	3.4818721019188
N	8.9693469138278	5.9840816217323	4.6808752694151
C	8.7712884400556	7.0656881535244	5.5084461146228
C	8.9226700710432	7.2231316188175	6.8989072667406
N	9.2835150405811	6.2597151941031	7.7292928804949
N	8.6968542500319	8.4706849664406	7.4061790420669
C	8.3352956804984	9.4600490350692	6.5972267720173
N	8.1487642688093	9.4335513535611	5.2656273572664
C	8.3918079944305	8.1954171991979	4.7862782492954
H	7.2420000000000	6.6690000000000	-1.1200000000000
H	8.6860000000000	7.2540000000000	-1.8990000000000
H	7.7328117979061	8.9076395323484	-0.3936057874741
H	10.3345160379468	7.6939988507305	0.7007918298816
H	10.1261298050829	9.2899250594410	2.5640945568565
H	9.7555267739754	11.0520762232875	1.0292704634246
H	7.2910091304968	9.2747666299353	2.5182563638255
H	8.7758400290233	5.9198078016996	2.5432439499687
H	9.2252181434364	6.4713704695335	8.7273953632479
H	9.1678618228840	5.3050586026486	7.3948349785195
H	8.1733600779705	10.4278085309747	7.0807047594235
H	9.6930000000000	3.2440000000000	0.2980000000000
P	11.7170595378573	9.2490145443248	-1.2326337086834
O	12.4371968541498	10.5347201043372	-1.3945340839021
O	11.3857135445084	8.6235420426940	-2.6634209628331
O	12.4278740405282	8.1244007557122	-0.3579586371728
C	13.8290469294670	7.7517616256237	-0.5046083220306
C	14.4729112280896	7.6721658625315	0.8656081284809
O	14.8252581945457	9.0108168248043	1.3267223572852
C	13.5504809211603	7.0621169682498	1.9319279314371
O	14.2826594647481	6.2689545137348	2.8843929231318
C	13.0294230088901	8.3035260525646	2.6431187570317
O	12.4801506457952	8.0167927584255	3.8926162057409
C	14.2690928418900	9.2210314471530	2.6203797826274
N	13.9494765264270	10.6464192201507	2.7546903307639
C	14.2157510215644	11.2782775803099	3.9688119799194
O	14.6867416085338	10.6829664979714	4.9364993045797
N	13.8975766728862	12.6165832364984	4.0045957571718
C	13.1667557408264	13.3211058988094	3.0578567563013
O	12.7971241324187	14.4897323340940	3.2840734034542
C	12.8985975104441	12.5906038909249	1.8510356324190
C	13.3126807733882	11.3060611757461	1.7402951595700

H	14.3629998466575	8.4844726401253	-1.1177638422161
H	13.8504519470017	6.7638688658297	-0.9750708855219
H	15.4005855754646	7.0885883479467	0.7808760910248
H	12.7630671316699	6.4466962969694	1.4909008325842
H	12.3042129433145	8.7694080463575	1.9652109840273
H	12.0090763018532	8.8088445934051	4.1995070394198
H	14.9728588286588	8.9872848019944	3.4279663782378
H	14.0469731829313	13.0718404353878	4.9202866799912
H	12.3260112672882	13.0507867974917	1.0577754286490
H	13.1789427708957	10.7477965142341	0.8180758458069
H	11.0483846926702	7.7103791644484	-2.6311717048798
P	14.5632820497375	4.7006400215436	2.5989708552449
O	13.8616818410707	4.1594913052114	1.4022212768497
O	14.2872392332055	4.0297963343216	4.0115095826043
O	16.1505508077580	4.5418001205773	2.4799738333450
C	16.9860066442356	5.2220680529773	3.4697831962718
C	18.2558692810147	5.6662262937094	2.7806493663137
O	17.8846165229940	6.3944276690416	1.6073193619196
C	19.0667092648422	6.6859466855281	3.5861576510017
O	19.9448321102313	6.0349137100533	4.5246038135678
C	19.8540821853618	7.4398863327401	2.5031798977196
O	21.0649478992624	6.7678575985047	2.1742622483914
C	18.9084002882142	7.3566080844485	1.2968871585941
N	18.2740000000000	8.5990000000000	0.9700000000000
C	17.8880000000000	8.9670000000000	-0.2680000000000
N	17.3930000000000	10.1769999999999	-0.3330000000000
C	17.4050000000000	10.6050000000000	0.9390000000000
C	17.0189999999999	11.8330000000000	1.5200000000000
N	16.5350000000000	12.9060000000000	0.7850000000000
N	17.1380000000000	11.9809999999999	2.8660000000000
C	17.5930000000000	10.9399999999999	3.5710000000000
N	17.9939999999999	9.7380000000000	3.1370000000000
C	17.8949999999999	9.6300000000000	1.7900000000000
H	17.2166301224555	4.4947227762107	4.2560154327883
H	16.4549386037238	6.1004887435667	3.8521556367126
H	18.8911972290316	4.8072424361753	2.5104731479885
H	18.3972636848800	7.4142246536030	4.0581157319223
H	20.0255734789650	8.4887017581246	2.7840128404724
H	21.4248512572877	6.4477654934317	3.0181429164426
H	19.4766214754808	7.0251572030499	0.4146492870595
H	18.0210000000000	8.2990000000000	-1.1070000000000
H	16.2979999999999	13.7639999999999	1.2660000000000
H	16.4009999999999	12.8100000000000	-0.2120000000000
H	17.6589999999999	11.0960000000000	4.6390000000000
H	13.5051516905727	4.3961394053360	4.4635253039567
P	20.0743235598218	6.5777920786876	6.0514977513313
O	21.1366964917981	5.8157135837835	6.7585239672570
O	18.5962387784030	6.5714193341191	6.6291449812093
O	20.4148273507524	8.1350212727089	5.9472538805080

C	19.4476292201321	9.2103376193262	5.9770651143090
C	19.4032124673182	9.8709787217294	7.3577512736757
O	18.5399617934039	9.1304130607502	8.2023899224737
C	20.7462106256515	9.9324721375785	8.1008648323459
O	21.4306067094009	11.0992585699772	7.7750498457460
C	20.3149765708991	9.9274641070684	9.5893655235761
O	20.3310305799852	11.2045370067562	10.1511653465194
C	18.8734210041159	9.3485044943600	9.5429559909009
N	18.7467022730870	8.0935895552136	10.2441210024739
C	18.2814128833256	6.9110421299996	9.7254396116030
N	18.2093232158703	5.9523046631555	10.5944004052617
C	18.6365974197236	6.5018892580183	11.7737491821169
C	18.6831405186989	6.0240585228066	13.0978270562141
N	18.3427343429487	4.7872689205166	13.4443983781072
N	19.0660290430467	6.8917881152053	14.0565619495902
C	19.3823111601845	8.1276286708565	13.7230576077920
N	19.3575753416678	8.6997025296639	12.5308294546492
C	18.9718149557389	7.8502588973869	11.5667768544043
H	18.4345914277602	8.8543965112269	5.7842814862640
H	19.7392301718576	9.8780204718589	5.1592852014646
H	19.0224943626549	10.8996106435166	7.2453825209670
H	21.3524312685941	9.0444920213638	7.9016300899393
H	20.9434782756797	9.2625768557050	10.1949802284831
H	20.7146923212251	11.8096094728366	9.4826735245594
H	18.1927686492626	10.0780325836716	10.0225241851023
H	18.0039011767761	6.8315430198026	8.6954170167158
H	18.2054939995698	4.5842415509552	14.4221622860205
H	18.0007126917310	4.1670354705189	12.7273517090880
H	19.6827600330867	8.7808882497237	14.5348243546087
H	18.1158244410148	5.7417869505938	6.4468334630631
P	22.9186729131794	11.0447658442853	7.1446650499930
O	23.7465931927701	9.9575423800605	7.6838872525429
O	23.3909521441436	12.4982666731263	7.4059565450130
O	22.7381686871676	11.0189132195439	5.5515595584260
C	22.8804849716077	9.8558169270406	4.7497032922950
C	22.3829999999999	10.1809999999999	3.3370000000000
O	21.0390000000000	10.6460000000000	3.3780000000000
C	23.1159999999999	11.3339999999999	2.6680000000000
O	24.3079999999999	10.8439999999999	2.0620000000000
C	22.0940000000000	11.8710000000000	1.6760000000000
O	21.9130000000000	11.0820000000000	0.5100000000000
C	20.8219999999999	11.7509999999999	2.5350000000000
N	20.3640000000000	12.9280000000000	3.2760000000000
C	20.0090000000000	14.0470000000000	2.5440000000000
O	19.9220000000000	14.1099999999999	1.3180000000000
N	19.7480000000000	15.1530000000000	3.3070000000000
C	19.7899999999999	15.3209999999999	4.6580000000000
O	19.5360000000000	16.3909999999999	5.2050000000000
C	20.1589999999999	14.1059999999999	5.3580000000000

C	20.4299999999999	12.9909999999999	4.6530000000000
H	22.3007596256970	9.0289159077320	5.1625258024842
H	23.9325128540006	9.5657027834383	4.6944917464901
H	22.4179999999999	9.2940000000000	2.7000000000000
H	23.3739999999999	12.0969999999999	3.4060000000000
H	22.2979999999999	12.9060000000000	1.4200000000000
H	22.6619999999999	11.2159999999999	-0.0720000000000
H	19.9510000000000	11.5060000000000	1.9240000000000
H	19.4759999999999	15.9770000000000	2.7870000000000
H	20.1939999999999	14.1010000000000	6.4350000000000
H	20.6789999999999	12.0649999999999	5.1450000000000
H	22.7024966943143	13.1586329386638	7.2308786985658
P	25.5979999999999	11.7989999999999	1.7640000000000
O	26.7970000000000	11.0920000000000	1.2370000000000
O	25.8380000000000	12.5690000000000	3.1720000000000
O	25.0070000000000	12.9700000000000	0.8020000000000
C	24.7549999999999	12.7210000000000	-0.5700000000000
C	24.1269999999999	13.9680000000000	-1.2160000000000
O	22.9570000000000	14.3620000000000	-0.5050000000000
C	24.9690000000000	15.2279999999999	-1.1240000000000
O	26.0479999999999	15.1820000000000	-2.0530000000000
C	23.9549999999999	16.3509999999999	-1.3090000000000
O	23.5100000000000	16.5509999999999	-2.6400000000000
C	22.7869999999999	15.7629999999999	-0.4920000000000
N	22.6140000000000	16.2869999999999	0.8660000000000
C	22.1269999999999	17.5809999999999	1.0230000000000
O	21.8290000000000	18.2340000000000	0.0210000000000
N	22.0309999999999	18.0549999999999	2.3470000000000
C	22.5459999999999	17.4280000000000	3.3430000000000
N	22.4950000000000	17.9400000000000	4.5750000000000
C	23.1810000000000	16.1570000000000	3.1770000000000
C	23.1859999999999	15.6440000000000	1.9400000000000
H	24.0949999999999	11.8640000000000	-0.6860000000000
H	25.6849999999999	12.4640000000000	-1.0800000000000
H	23.8619999999999	13.7609999999999	-2.2550000000000
H	25.4200000000000	15.2919999999999	-0.1320000000000
H	24.3039999999999	17.2809999999999	-0.8670000000000
H	22.9589999999999	17.3380000000000	-2.6640000000000
H	21.8129999999999	15.9320000000000	-0.9620000000000
H	22.8949999999999	17.4490000000000	5.3570000000000
H	22.0640000000000	18.8410000000000	4.7170000000000
H	23.6370000000000	15.6189999999999	3.9940000000000
H	23.6389999999999	14.6929999999999	1.7180000000000
H	26.7540000000000	12.5389999999999	3.5090000000000
P	27.1819999999999	16.3550000000000	-2.1380000000000
O	28.3309999999999	16.0640000000000	-3.0370000000000
O	27.5919999999999	16.5910000000000	-0.5850000000000
O	26.3369999999999	17.6980000000000	-2.4960000000000
C	26.1419999999999	18.1030000000000	-3.8410000000000

C	25.8689999999999	19.6140000000000	-3.8740000000000
O	24.7139999999999	19.9130000000000	-3.0990000000000
C	26.9080000000000	20.4669999999999	-3.1680000000000
O	28.0869999999999	20.6020000000000	-3.9550000000000
C	26.1589999999999	21.7590000000000	-2.8790000000000
O	26.0210000000000	22.6219999999999	-3.9960000000000
C	24.7860000000000	21.1840000000000	-2.4990000000000
N	24.5869999999999	21.0560000000000	-1.0020000000000
C	24.9319999999999	20.0530000000000	-0.1670000000000
N	24.6610000000000	20.2959999999999	1.0940000000000
C	24.1479999999999	21.5360000000000	1.0800000000000
C	23.6950000000000	22.3520000000000	2.1880000000000
O	23.6179999999999	22.0569999999999	3.3790000000000
N	23.3329999999999	23.5889999999999	1.7140000000000
C	23.3509999999999	24.0309999999999	0.4030000000000
N	22.9720000000000	25.2929999999999	0.1910000000000
N	23.7169999999999	23.2860000000000	-0.5820000000000
C	24.1110000000000	22.0420000000000	-0.1910000000000
H	25.3150000000000	17.5519999999999	-4.2850000000000
H	27.0199999999999	17.8880000000000	-4.4500000000000
H	25.7119999999999	19.9540000000000	-4.9000000000000
H	27.2070000000000	19.9899999999999	-2.2320000000000
H	26.5820000000000	22.2910000000000	-2.0280000000000
H	26.8670000000000	23.0470000000000	-4.1590000000000
H	23.9609999999999	21.7869999999999	-2.8830000000000
H	25.3990000000000	19.1589999999999	-0.5470000000000
H	23.0120000000000	24.2369999999999	2.4220000000000
H	22.9639999999999	25.6619999999999	-0.7480000000000
H	22.7100000000000	25.8990000000000	0.9600000000000
H	28.5420000000000	16.4840000000000	-0.3860000000000
P	29.4319999999999	21.3099999999999	-3.3610000000000
O	30.5760000000000	21.3979999999999	-4.3070000000000
O	29.7349999999999	20.4510000000000	-2.0160000000000
O	28.8960000000000	22.7390000000000	-2.7940000000000
C	29.6370000000000	23.4349999999999	-1.8130000000000
C	28.8769999999999	24.7100000000000	-1.4240000000000
O	27.6000000000000	24.3760000000000	-0.8810000000000
C	29.4909999999999	25.4669999999999	-0.2600000000000
O	30.6570000000000	26.1769999999999	-0.6720000000000
C	28.3309999999999	26.3129999999999	0.2510000000000
O	27.9770000000000	27.4220000000000	-0.5600000000000
C	27.2119999999999	25.2560000000000	0.1500000000000
N	26.8750000000000	24.5390000000000	1.3830000000000
C	26.2770000000000	25.2500000000000	2.4170000000000
O	25.9160000000000	26.4119999999999	2.2160000000000
N	26.1340000000000	24.5779999999999	3.6470000000000
C	26.6370000000000	23.4130000000000	3.8600000000000
N	26.5180000000000	22.8200000000000	5.0490000000000
C	27.3249999999999	22.6990000000000	2.8290000000000

C	27.4110000000000	23.2950000000000	1.6320000000000
H	30.6179999999999	23.6909999999999	-2.2170000000000
H	29.7950000000000	22.8009999999999	-0.9380000000000
H	28.7459999999999	25.3599999999999	-2.2900000000000
H	29.8060000000000	24.7519999999999	0.5040000000000
H	28.4810000000000	26.6230000000000	1.2810000000000
H	27.3109999999999	27.9359999999999	-0.0970000000000
H	26.2549999999999	25.6780000000000	-0.1720000000000
H	26.9029999999999	21.9009999999999	5.2060000000000
H	26.0169999999999	23.2809999999999	5.7970000000000
H	27.7519999999999	21.7190000000000	2.9830000000000
H	27.8990000000000	22.8210000000000	0.7940000000000
H	30.5960000000000	19.9909999999999	-2.0050000000000
P	31.7089999999999	26.8339999999999	0.3930000000000
O	32.9769999999999	27.3419999999999	-0.1990000000000
O	31.9110000000000	25.6679999999999	1.5040000000000
O	30.8309999999999	27.9450000000000	1.1890000000000
C	30.5539999999999	29.1980000000000	0.5940000000000
C	29.8410000000000	30.0990000000000	1.6130000000000
O	28.6799999999999	29.4589999999999	2.1300000000000
C	30.6230000000000	30.3900000000000	2.8830000000000
O	31.5500000000000	31.4379999999999	2.6290000000000
C	29.5369999999999	30.7880000000000	3.8660000000000
O	29.0130000000000	32.0949999999999	3.6850000000000
C	28.4680000000000	29.7489999999999	3.4920000000000
N	28.5289999999999	28.4619999999999	4.2980000000000
C	29.0760000000000	27.2689999999999	3.9800000000000
N	28.9220000000000	26.3490000000000	4.9000000000000
C	28.2940000000000	26.9939999999999	5.8920000000000
C	27.8949999999999	26.4909999999999	7.1870000000000
O	27.9639999999999	25.3410000000000	7.6140000000000
N	27.3949999999999	27.5289999999999	7.9310000000000
C	27.2270000000000	28.8470000000000	7.5440000000000
N	26.7270000000000	29.6750000000000	8.4630000000000
N	27.5279999999999	29.2749999999999	6.3640000000000
C	28.0719999999999	28.3060000000000	5.5720000000000
H	29.9390000000000	29.0659999999999	-0.2950000000000
H	31.4759999999999	29.6769999999999	0.2630000000000
H	29.5390000000000	31.0309999999999	1.1320000000000
H	31.1350000000000	29.4929999999999	3.2380000000000
H	29.8539999999999	30.6479999999999	4.8980000000000
H	29.6619999999999	32.7299999999999	3.9960000000000
H	27.4549999999999	30.1419999999999	3.6090000000000
H	29.5790000000000	27.1189999999999	3.0410000000000
H	27.1110000000000	27.2740000000000	8.8700000000000
H	26.5779999999999	30.6469999999999	8.2390000000000
H	26.5240000000000	29.3449999999999	9.4010000000000
H	32.8389999999999	25.4239999999999	1.6860000000000
P	33.1210000000000	31.3470000000000	3.0690000000000

O	34.0339999999999	32.2650000000000	2.3380000000000
O	33.4939999999999	29.7719999999999	2.9420000000000
O	33.0469999999999	31.5960000000000	4.6740000000000
C	32.7070000000000	32.8870000000000	5.1560000000000
C	32.3040000000000	32.8350000000000	6.6350000000000
O	31.1340000000000	32.0390000000000	6.7990000000000
C	33.2790000000000	32.1240000000000	7.5600000000000
O	34.4249999999999	32.9279999999999	7.8190000000000
C	32.4149999999999	31.8090000000000	8.7730000000000
O	32.1150000000000	32.9149999999999	9.6110000000000
C	31.1329999999999	31.3689999999999	8.0400000000000
N	30.9639999999999	29.9280000000000	7.8690000000000
C	30.5869999999999	29.2029999999999	8.9830000000000
O	30.1909999999999	29.6670000000000	10.0519999999999
N	30.7240000000000	27.8500000000000	8.8370000000000
C	31.2220000000000	27.1230000000000	7.7970000000000
O	31.2899999999999	25.8979999999999	7.8200000000000
C	31.6370000000000	27.9499999999999	6.6830000000000
C	31.4939999999999	29.2860000000000	6.7680000000000
H	31.8859999999999	33.3100000000000	4.5750000000000
H	33.5600000000000	33.5559999999999	5.0230000000000
H	32.0900000000000	33.8440000000000	6.9960000000000
H	33.6210000000000	31.2029999999999	7.0860000000000
H	32.8239999999999	30.9849999999999	9.3500000000000
H	32.8810000000000	33.1019999999999	10.1570000000000
H	30.2210000000000	31.6980000000000	8.5470000000000
H	30.4340000000000	27.3069999999999	9.6400000000000
H	32.0519999999999	27.4879999999999	5.8000000000000
H	31.7929999999999	29.9370000000000	5.9630000000000
H	34.2389999999999	29.5689999999999	2.3430000000000
P	35.8310000000000	32.2929999999999	8.3640000000000
O	36.9889999999999	33.2269999999999	8.3520000000000
O	36.0349999999999	30.9619999999999	7.4570000000000
O	35.4549999999999	31.6870000000000	9.8260000000000
C	35.2719999999999	32.5690000000000	10.9179999999999
C	34.7069999999999	31.8120000000000	12.1280000000000
O	33.5499999999999	31.0710000000000	11.7460000000000
C	35.5790000000000	30.7049999999999	12.6969999999999
O	36.6469999999999	31.2160000000000	13.4870000000000
C	34.5630000000000	29.8859999999999	13.4840000000000
O	34.0709999999999	30.4899999999999	14.6730000000000
C	33.4320000000000	29.8599999999999	12.4529999999999
N	33.5129999999999	28.6709999999999	11.5139999999999
C	33.9819999999999	28.5730000000000	10.2509999999999
N	33.9530000000000	27.3539999999999	9.7700000000000
C	33.4819999999999	26.6149999999999	10.7840000000000
C	33.2719999999999	25.1829999999999	10.8559999999999
O	33.3870000000000	24.3430000000000	9.9680000000000
N	32.9099999999999	24.8490000000000	12.1379999999999

C	32.7090000000000	25.6990000000000	13.2100000000000
N	32.3840000000000	25.1329999999999	14.3729999999999
N	32.8250000000000	26.9800000000000	13.1170000000000
C	33.2239999999999	27.3900000000000	11.8789999999999
H	34.5880000000000	33.3710000000000	10.6359999999999
H	36.2180000000000	33.0469999999999	11.1780000000000
H	34.4399999999999	32.5249999999999	12.9100000000000
H	36.0150000000000	30.1230000000000	11.8829999999999
H	34.9099999999999	28.8719999999999	13.6790000000000
H	33.5270000000000	31.2450000000000	14.4369999999999
H	32.4440000000000	29.8200000000000	12.9190000000000
H	34.3500000000000	29.4379999999999	9.7240000000000
H	32.7710000000000	23.8590000000000	12.2959999999999
H	32.2160000000000	25.7139999999999	15.1799999999999
H	32.3280000000000	24.1239999999999	14.4680000000000
H	36.8920000000000	30.9029999999999	6.9930000000000
P	37.9219999999999	30.2830000000000	13.9120000000000
O	38.9549999999999	30.9589999999999	14.7420000000000
O	38.4699999999999	29.7310000000000	12.4870000000000
O	37.2550000000000	28.9540000000000	14.5770000000000
C	36.8179999999999	28.9630000000000	15.9250000000000
C	36.4620000000000	27.5369999999999	16.3700000000000
O	35.5200000000000	26.9269999999999	15.4890000000000
C	37.6059999999999	26.5489999999999	16.2800000000000
O	38.5579999999999	26.7860000000000	17.3099999999999
C	36.8849999999999	25.2079999999999	16.3210000000000
O	36.3329999999999	24.8460000000000	17.5770000000000
C	35.7259999999999	25.5339999999999	15.3680000000000
N	36.0099999999999	25.1709999999999	13.9230000000000
C	36.4059999999999	25.9639999999999	12.9030000000000
N	36.6090000000000	25.3170000000000	11.7810000000000
C	36.3459999999999	24.0390000000000	12.0879999999999
C	36.4099999999999	22.8649999999999	11.2390000000000
O	36.7010000000000	22.7890000000000	10.0480000000000
N	36.0949999999999	21.7560000000000	11.9830000000000
C	35.7859999999999	21.7020000000000	13.3300000000000
N	35.5989999999999	20.4789999999999	13.8399999999999
N	35.6929999999999	22.7549999999999	14.0730000000000
C	35.9869999999999	23.9080000000000	13.4009999999999
H	35.9630000000000	29.6219999999999	16.0489999999999
H	37.6000000000000	29.3610000000000	16.5740000000000
H	36.0499999999999	27.5590000000000	17.3810000000000
H	38.1229999999999	26.6789999999999	15.3260000000000
H	37.4930000000000	24.4020000000000	15.9179999999999
H	37.0369999999999	24.5090000000000	18.1350000000000
H	34.8059999999999	25.0159999999999	15.6470000000000
H	36.5279999999999	27.0279999999999	13.0440000000000
H	36.1280000000000	20.8870000000000	11.4730000000000
H	35.3830000000000	20.3769999999999	14.8190000000000

H	35.6769999999999	19.6550000000000	13.2620000000000
H	39.4139999999999	29.9050000000000	12.3109999999999
P	40.0390000000000	26.1000000000000	17.2979999999999
O	40.9819999999999	26.6140000000000	18.3279999999999
O	40.5429999999999	26.2910000000000	15.7680000000000
O	39.7269999999999	24.5070000000000	17.3870000000000
C	39.3449999999999	23.9469999999999	18.6290000000000
C	39.1899999999999	22.4299999999999	18.4849999999999
O	38.3179999999999	22.0949999999999	17.4100000000000
C	40.4530000000000	21.6990000000000	18.0740000000000
O	41.3040000000000	21.5799999999999	19.2079999999999
C	39.9029999999999	20.3700000000000	17.5859999999999
O	39.4939999999999	19.4950000000000	18.6250000000000
C	38.6599999999999	20.8590000000000	16.8260000000000
N	38.8609999999999	21.0279999999999	15.3300000000000
C	39.0260000000000	22.1580000000000	14.6120000000000
N	39.1169999999999	21.9559999999999	13.3209999999999
C	39.0679999999999	20.6219999999999	13.1899999999999
C	39.1240000000000	19.7830000000000	12.0530000000000
N	39.2070000000000	20.2650000000000	10.7509999999999
N	39.1189999999999	18.4370000000000	12.2420000000000
C	39.0480000000000	17.9819999999999	13.4990000000000
N	38.9399999999999	18.6720000000000	14.6449999999999
C	38.9540000000000	20.0079999999999	14.4239999999999
H	38.4189999999999	24.3949999999999	18.9830000000000
H	40.0959999999999	24.1660000000000	19.3900000000000
H	38.7899999999999	22.0139999999999	19.4119999999999
H	40.9620000000000	22.2360000000000	17.2699999999999
H	40.5859999999999	19.8739999999999	16.9020000000000
H	39.1569999999999	18.6840000000000	18.2250000000000
H	37.8119999999999	20.1810000000000	16.9430000000000
H	39.0519999999999	23.1219999999999	15.0960000000000
H	39.2349999999999	19.6000000000000	9.9880000000000
H	39.1659999999999	21.2609999999999	10.5899999999999
H	39.0989999999999	16.9100000000000	13.6099999999999
H	41.3950000000000	26.7579999999999	15.6679999999999
P	42.9320000000000	21.6239999999999	19.0910000000000
O	43.6529999999999	21.7100000000000	20.3889999999999
O	43.2189999999999	22.8560000000000	18.0740000000000
O	43.2849999999999	20.3109999999999	18.1980000000000
C	43.0319999999999	19.0169999999999	18.7180000000000
C	43.2340000000000	17.9720000000000	17.6129999999999
O	42.4169999999999	18.3309999999999	16.5000000000000
C	44.6610000000000	17.9059999999999	17.0530000000000
O	45.0219999999999	16.5450000000000	16.8859999999999
C	44.6000000000000	18.6090000000000	15.7110000000000
O	45.4780000000000	18.1769999999999	14.6920000000000
C	43.1529999999999	18.3449999999999	15.3140000000000
N	42.6090000000000	19.2979999999999	14.3590000000000

C	42.5319999999999	18.9310000000000	13.0310000000000
O	42.5910000000000	17.7890000000000	12.5790000000000
N	42.4179999999999	19.9899999999999	12.1739999999999
C	42.3710000000000	21.3279999999999	12.4250000000000
O	42.2989999999999	22.1660000000000	11.5299999999999
C	42.4020000000000	21.6310000000000	13.8430000000000
C	42.5089999999999	20.6219999999999	14.7279999999999
H	42.0129999999999	18.9529999999999	19.1000000000000
H	43.6910000000000	18.8120000000000	19.5629999999999
H	42.9229999999999	16.9959999999999	17.9920000000000
H	45.4089999999999	18.3859999999999	17.6890000000000
H	44.7340000000000	19.6789999999999	15.8750000000000
H	46.3779999999999	18.4319999999999	14.9260000000000
H	43.0450000000000	17.3389999999999	14.8970000000000
H	42.3939999999999	19.7369999999999	11.1940000000000
H	42.3359999999999	22.6580000000000	14.1720000000000
H	42.5480000000000	20.7850000000000	15.7959999999999
H	43.7790000000000	23.5689999999999	18.4349999999999
P	45.8089999999999	15.7750000000000	18.0859999999999
O	45.4390000000000	16.1829999999999	19.4699999999999
O	47.3689999999999	15.9990000000000	17.7070000000000
O	45.5579999999999	14.2119999999999	17.7270000000000
C	44.3890000000000	13.5670000000000	18.1990000000000
C	44.5399999999999	12.0470000000000	18.0489999999999
O	44.5020000000000	11.7070000000000	16.6720000000000
C	45.8770000000000	11.4879999999999	18.5199999999999
O	45.6499999999999	10.2059999999999	19.0919999999999
C	46.7399999999999	11.3889999999999	17.2680000000000
O	47.7139999999999	10.3610000000000	17.3019999999999
C	45.6910000000000	11.1560000000000	16.1780000000000
N	46.0150000000000	11.8030000000000	14.8450000000000
C	45.7610000000000	13.0649999999999	14.4410000000000
N	46.2040000000000	13.3330000000000	13.2379999999999
C	46.7980000000000	12.1959999999999	12.8439999999999
C	47.4849999999999	11.8539999999999	11.6560000000000
N	47.7000000000000	12.7449999999999	10.6120000000000
N	47.9650000000000	10.5890000000000	11.5250000000000
C	47.7689999999999	9.7380000000000	12.5419999999999
N	47.1679999999999	9.9490000000000	13.7219999999999
C	46.6929999999999	11.2159999999999	13.8149999999999
H	43.5149999999999	13.9309999999999	17.6580000000000
H	44.2250000000000	13.8019999999999	19.2530000000000
H	43.6950000000000	11.5640000000000	18.5420000000000
H	46.3680000000000	12.1199999999999	19.2650000000000
H	47.2220000000000	12.3510000000000	17.0829999999999
H	48.3229999999999	10.5459999999999	18.0210000000000
H	45.5119999999999	10.0930000000000	16.0030000000000
H	45.2349999999999	13.7550000000000	15.0860000000000
H	48.2790000000000	12.4529999999999	9.8390000000000

H	47.4200000000000	13.7119999999999	10.7270000000000
H	48.1629999999999	8.7440000000000	12.3930000000000
H	47.9290000000000	16.3219999999999	18.4400000000000
P	45.5709999999999	10.0530000000000	20.7130000000000
O	46.8170000000000	10.4429999999999	21.4269999999999
O	45.1289999999999	8.5040000000000	20.9149999999999
O	44.2529999999999	10.9410000000000	21.1020000000000
C	42.9900000000000	10.3640000000000	21.4089999999999
C	42.2689999999999	11.1929999999999	22.4879999999999
O	43.1280000000000	11.2949999999999	23.6260000000000
C	41.9560000000000	12.6530000000000	22.1219999999999
O	40.7710000000000	13.0389999999999	22.8180000000000
C	43.1210000000000	13.4380000000000	22.6990000000000
O	42.9109999999999	14.8179999999999	22.9439999999999
C	43.3310000000000	12.6329999999999	23.9830000000000
N	44.5970000000000	12.7980000000000	24.6849999999999
C	44.6069999999999	13.1980000000000	26.0079999999999
O	43.6310000000000	13.4979999999999	26.6939999999999
N	45.8590000000000	13.2530000000000	26.5599999999999
C	47.0739999999999	12.9680000000000	26.0100000000000
O	48.1250000000000	13.0649999999999	26.6370000000000
C	46.9799999999999	12.5399999999999	24.6269999999999
C	45.7700000000000	12.4770000000000	24.0399999999999
H	43.0949999999999	9.3470000000000	21.7890000000000
H	42.3870000000000	10.2949999999999	20.5030000000000
H	41.3560000000000	10.6669999999999	22.7710000000000
H	41.8290000000000	12.8369999999999	21.0530000000000
H	43.9919999999999	13.3209999999999	22.0530000000000
H	42.0300000000000	14.9299999999999	23.3109999999999
H	42.5279999999999	12.8699999999999	24.6870000000000
H	45.8860000000000	13.5510000000000	27.5229999999999
H	47.8759999999999	12.2739999999999	24.0850000000000
H	45.6379999999999	12.1560000000000	23.0169999999999
H	45.7479999999999	7.9610000000000	21.4400000000000
P	39.3029999999999	12.9749999999999	22.1129999999999
O	38.1469999999999	13.2100000000000	23.0180000000000
O	39.2979999999999	11.5359999999999	21.3599999999999
O	39.4519999999999	14.0280000000000	20.8859999999999
C	39.4099999999999	15.4250000000000	21.1099999999999
C	39.2389999999999	16.1469999999999	19.7650000000000
O	40.3890000000000	15.9239999999999	18.9540000000000
C	38.1099999999999	15.6250000000000	18.8840000000000
O	36.8699999999999	16.2049999999999	19.2809999999999
C	38.5609999999999	15.9789999999999	17.4740000000000
O	38.3439999999999	17.3369999999999	17.1419999999999
C	40.0709999999999	15.7319999999999	17.5990000000000
N	40.5409999999999	14.3539999999999	17.1720000000000
C	40.4359999999999	13.1869999999999	17.8419999999999
N	40.9489999999999	12.1620000000000	17.2109999999999

C	41.3659999999999	12.6820000000000	16.0509999999999
C	42.0020000000000	12.0039999999999	14.9459999999999
O	42.3049999999999	10.8170000000000	14.8550000000000
N	42.2199999999999	12.9039999999999	13.9359999999999
C	41.9219999999999	14.2530000000000	13.9269999999999
N	42.2299999999999	14.8979999999999	12.8010000000000
N	41.3789999999999	14.8620000000000	14.9290000000000
C	41.1129999999999	14.0250000000000	15.9749999999999
H	40.3220000000000	15.7509999999999	21.6110000000000
H	38.5759999999999	15.6790000000000	21.7680000000000
H	39.1409999999999	17.2199999999999	19.9299999999999
H	38.0120000000000	14.5429999999999	18.9860000000000
H	38.1340000000000	15.3179999999999	16.7240000000000
H	38.5170000000000	17.4649999999999	16.2029999999999
H	40.6439999999999	16.4540000000000	17.0150000000000
H	39.9799999999999	13.1379999999999	18.8150000000000
H	42.6450000000000	12.5120000000000	13.1050000000000
H	42.1000000000000	15.8989999999999	12.7370000000000
H	42.6099999999999	14.3989999999999	12.0069999999999
H	38.5210000000000	10.9740000000000	21.5450000000000
P	35.4279999999999	15.6739999999999	18.7259999999999
O	34.2379999999999	16.3769999999999	19.2749999999999
O	35.4720000000000	14.0839999999999	19.0509999999999
O	35.5709999999999	15.7100000000000	17.1050000000000
C	35.1890000000000	16.8560000000000	16.3640000000000
C	35.3609999999999	16.5710000000000	14.8620000000000
O	36.7049999999999	16.1619999999999	14.6039999999999
C	34.5919999999999	15.3719999999999	14.3260000000000
O	33.2269999999999	15.6460000000000	14.0320000000000
C	35.3609999999999	14.9990000000000	13.0709999999999
O	35.1540000000000	15.8680000000000	11.9659999999999
C	36.7869999999999	15.1739999999999	13.6020000000000
N	37.3819999999999	13.8960000000000	14.1720000000000
C	37.0979999999999	13.2759999999999	15.3399999999999
N	37.6649999999999	12.1069999999999	15.4760000000000
C	38.3220000000000	11.9350000000000	14.3230000000000
C	39.0570000000000	10.7759999999999	13.8740000000000
O	39.2449999999999	9.7120000000000	14.4610000000000
N	39.5529999999999	11.0220000000000	12.6189999999999
C	39.3950000000000	12.1600000000000	11.8550000000000
N	39.9729999999999	12.1310000000000	10.6530000000000
N	38.7280000000000	13.1899999999999	12.2569999999999
C	38.1989999999999	13.0259999999999	13.5039999999999
H	35.7899999999999	17.7160000000000	16.6589999999999
H	34.1510000000000	17.1119999999999	16.5770000000000
H	35.1360000000000	17.4660000000000	14.2769999999999
H	34.6409999999999	14.5630000000000	15.0549999999999
H	35.1820000000000	13.9600000000000	12.7959999999999
H	34.2719999999999	15.7110000000000	11.6189999999999

H	37.4659999999999	15.5250000000000	12.8239999999999
H	36.4389999999999	13.7230000000000	16.0670000000000
H	40.0790000000000	10.2609999999999	12.2089999999999
H	39.9170000000000	12.9380000000000	10.0429999999999
H	40.4479999999999	11.3019999999999	10.3200000000000
H	34.7310000000000	13.7509999999999	19.5919999999999
P	32.0660000000000	14.5860000000000	14.4770000000000
O	32.5420000000000	13.2360000000000	14.8859999999999
O	31.0629999999999	14.5770000000000	13.1999999999999
O	31.2390000000000	15.3979999999999	15.6229999999999
C	30.4100000000000	16.5000000000000	15.2949999999999
C	30.8109999999999	17.7459999999999	16.0970000000000
O	32.0409999999999	18.2789999999999	15.6150000000000
C	29.8560000000000	18.9089999999999	15.8949999999999
O	28.6960000000000	18.7469999999999	16.7079999999999
C	30.7059999999999	20.1359999999999	16.1859999999999
O	30.9710000000000	20.3790000000000	17.5579999999999
C	32.0170000000000	19.6840000000000	15.5069999999999
N	32.2670000000000	20.1409999999999	14.1349999999999
C	32.3669999999999	21.5049999999999	13.8949999999999
O	32.3329999999999	22.2860000000000	14.8469999999999
N	32.5230000000000	21.9009999999999	12.5510000000000
C	32.5369999999999	21.0609999999999	11.5790000000000
N	32.7190000000000	21.4720000000000	10.3219999999999
C	32.3750000000000	19.6570000000000	11.8030000000000
C	32.2419999999999	19.2579999999999	13.0749999999999
H	29.3900000000000	16.2119999999999	15.5510000000000
H	30.4170000000000	16.7250000000000	14.2270000000000
H	30.9110000000000	17.5120000000000	17.1589999999999
H	29.5199999999999	18.9250000000000	14.8559999999999
H	30.2940000000000	21.0219999999999	15.7100000000000
H	31.4220000000000	21.2240000000000	17.6389999999999
H	32.9050000000000	20.0399999999999	16.0390000000000
H	32.7349999999999	20.8140000000000	9.5590000000000
H	32.8629999999999	22.4570000000000	10.1310000000000
H	32.3629999999999	18.9349999999999	11.0000000000000
H	32.1270000000000	18.2149999999999	13.3330000000000
H	30.8520000000000	13.6910000000000	12.8460000000000
P	27.3730000000000	19.6939999999999	16.5629999999999
O	26.2450000000000	19.3460000000000	17.4690000000000
O	27.0180000000000	19.5990000000000	14.9819999999999
O	27.9450000000000	21.2100000000000	16.7109999999999
C	27.9780000000000	21.8700000000000	17.9660000000000
C	27.9729999999999	23.3889999999999	17.7369999999999
O	29.0829999999999	23.7650000000000	16.9280000000000
C	26.8120000000000	23.9089999999999	16.9070000000000
O	25.6320000000000	23.9959999999999	17.6990000000000
C	27.3380000000000	25.2409999999999	16.3939999999999
O	27.3599999999999	26.2899999999999	17.3490000000000

C	28.7830000000000	24.8369999999999	16.0650000000000
N	28.9929999999999	24.4050000000000	14.6259999999999
C	28.9460000000000	23.1690000000000	14.0879999999999
N	29.1990000000000	23.1380000000000	12.8040000000000
C	29.3840000000000	24.4250000000000	12.4779999999999
C	29.6969999999999	25.0479999999999	11.2490000000000
N	29.9110000000000	24.3399999999999	10.0709999999999
N	29.8049999999999	26.4020000000000	11.2240000000000
C	29.6069999999999	27.0740000000000	12.3659999999999
N	29.3200000000000	26.6000000000000	13.5869999999999
C	29.2289999999999	25.2480000000000	13.5809999999999
H	28.8619999999999	21.5680000000000	18.5259999999999
H	27.1170000000000	21.5990000000000	18.5779999999999
H	28.0339999999999	23.9149999999999	18.6920000000000
H	26.6149999999999	23.2300000000000	16.0749999999999
H	26.8270000000000	25.5539999999999	15.4879999999999
H	27.5779999999999	27.1110000000000	16.8990000000000
H	29.4929999999999	25.6439999999999	16.2590000000000
H	28.7349999999999	22.3090000000000	14.7010000000000
H	30.1580000000000	24.8430000000000	9.2270000000000
H	29.8389999999999	23.3339999999999	10.0790000000000
H	29.7010000000000	28.1479999999999	12.2949999999999
H	27.7280000000000	19.8629999999999	14.3670000000000
P	24.1409999999999	24.0680000000000	17.0360000000000
O	23.0139999999999	24.0219999999999	18.0060000000000
O	24.1469999999999	22.8539999999999	15.9570000000000
O	24.1849999999999	25.3930000000000	16.0940000000000
C	24.0509999999999	26.6810000000000	16.6660000000000
C	24.2259999999999	27.7600000000000	15.5839999999999
O	25.4810000000000	27.6009999999999	14.9280000000000
C	23.2729999999999	27.6679999999999	14.4030000000000
O	21.9660000000000	28.1159999999999	14.7390000000000
C	23.9819999999999	28.4909999999999	13.3339999999999
O	23.9490000000000	29.8949999999999	13.5269999999999
C	25.4179999999999	27.9969999999999	13.5770000000000
N	25.8829999999999	26.9439999999999	12.6730000000000
C	26.2650000000000	27.2959999999999	11.3859999999999
O	26.2989999999999	28.4849999999999	11.0679999999999
N	26.5670000000000	26.2340000000000	10.5090000000000
C	26.3359999999999	25.0060000000000	10.8070000000000
N	26.5839999999999	24.0320000000000	9.9290000000000
C	25.8249999999999	24.6329999999999	12.0869999999999
C	25.6250000000000	25.6230000000000	12.9649999999999
H	24.7880000000000	26.8219999999999	17.4570000000000
H	23.0719999999999	26.7809999999999	17.1370000000000
H	24.1799999999999	28.7510000000000	16.0380000000000
H	23.1849999999999	26.6250000000000	14.0909999999999
H	23.6490000000000	28.2229999999999	12.3330000000000
H	23.0869999999999	30.2210000000000	13.2620000000000

H	26.1630000000000	28.7940000000000	13.4870000000000
H	26.3909999999999	23.0680000000000	10.1519999999999
H	26.9619999999999	24.2710000000000	9.0200000000000
H	25.6080000000000	23.6099999999999	12.3569999999999
H	25.2390000000000	25.4409999999999	13.9529999999999
H	23.4690000000000	22.1670000000000	16.1020000000000
P	20.6649999999999	27.7630000000000	13.8109999999999
O	19.3539999999999	28.1739999999999	14.3819999999999
O	20.8090000000000	26.1690000000000	13.5419999999999
O	21.0199999999999	28.4119999999999	12.3610000000000
C	20.8659999999999	29.8049999999999	12.1609999999999
C	21.3249999999999	30.1969999999999	10.7469999999999
O	22.6380000000000	29.7049999999999	10.4939999999999
C	20.5629999999999	29.5470000000000	9.6080000000000
O	19.2719999999999	30.1250000000000	9.4490000000000
C	21.5150000000000	29.7300000000000	8.4330000000000
O	21.6140000000000	31.0509999999999	7.9260000000000
C	22.8369999999999	29.3900000000000	9.1350000000000
N	23.2310000000000	27.9359999999999	8.9790000000000
C	23.0199999999999	26.8810000000000	9.7940000000000
N	23.4480000000000	25.7420000000000	9.3030000000000
C	23.9359999999999	26.0710000000000	8.0970000000000
C	24.5249999999999	25.2190000000000	7.0840000000000
O	24.7719999999999	24.0159999999999	7.1430000000000
N	24.7899999999999	25.9699999999999	5.9660000000000
C	24.5869999999999	27.3270000000000	5.7910000000000
N	24.9250000000000	27.8329999999999	4.6020000000000
N	24.1020000000000	28.0880000000000	6.7130000000000
C	23.7920000000000	27.4080000000000	7.8540000000000
H	21.4400000000000	30.3569999999999	12.9049999999999
H	19.8240000000000	30.0949999999999	12.3040000000000
H	21.3150000000000	31.2830000000000	10.6419999999999
H	20.4149999999999	28.4860000000000	9.8280000000000
H	21.3150000000000	29.0189999999999	7.6330000000000
H	22.1380000000000	31.0309999999999	7.1210000000000
H	23.6750000000000	29.9849999999999	8.7660000000000
H	22.5330000000000	27.0060000000000	10.7490000000000
H	25.1879999999999	25.4579999999999	5.1900000000000
H	24.8129999999999	28.8200000000000	4.4270000000000
H	25.2830000000000	27.2409999999999	3.8610000000000
H	20.0330000000000	25.6350000000000	13.7980000000000
P	18.1409999999999	29.4840000000000	8.4570000000000
O	16.8000000000000	30.1239999999999	8.5300000000000
O	18.1490000000000	27.9070000000000	8.8430000000000
O	18.8329999999999	29.4989999999999	6.9860000000000
C	18.8550000000000	30.6879999999999	6.2170000000000
C	19.4579999999999	30.3949999999999	4.8350000000000
O	20.7190000000000	29.7439999999999	4.9550000000000
C	18.6879999999999	29.3850000000000	4.0010000000000

O	17.5219999999999	29.9860000000000	3.4520000000000
C	19.7109999999999	28.9490000000000	2.9630000000000
O	19.9520000000000	29.8880000000000	1.9260000000000
C	20.9559999999999	28.8730000000000	3.8690000000000
N	21.3380000000000	27.5320000000000	4.3170000000000
C	21.9540000000000	26.6819999999999	3.4090000000000
O	22.2169999999999	27.0949999999999	2.2800000000000
N	22.2210000000000	25.3719999999999	3.8560000000000
C	21.7699999999999	24.9200000000000	4.9720000000000
N	22.0120000000000	23.6619999999999	5.3450000000000
C	21.0180000000000	25.7480000000000	5.8630000000000
C	20.8359999999999	27.0229999999999	5.4930000000000
H	19.4269999999999	31.4609999999999	6.7270000000000
H	17.8449999999999	31.0839999999999	6.1010000000000
H	19.5839999999999	31.3279999999999	4.2800000000000
H	18.3889999999999	28.5429999999999	4.6300000000000
H	19.4680000000000	27.9699999999999	2.5520000000000
H	19.2070000000000	29.8730000000000	1.3210000000000
H	21.8539999999999	29.2680000000000	3.3860000000000
H	21.6950000000000	23.3180000000000	6.2380000000000
H	22.5530000000000	23.0549999999999	4.7410000000000
H	20.6110000000000	25.3870000000000	6.7960000000000
H	20.2820000000000	27.7240000000000	6.0960000000000
H	17.2820000000000	27.5380000000000	9.0970000000000
P	16.2100000000000	29.1039999999999	3.0430000000000
O	15.0289999999999	29.8979999999999	2.6110000000000
O	15.9520000000000	28.1729999999999	4.3470000000000
O	16.7789999999999	28.0509999999999	1.9380000000000
C	16.9669999999999	28.4869999999999	0.6030000000000
C	17.5549999999999	27.3679999999999	-0.2690000000000
O	18.7830000000000	26.9020000000000	0.2800000000000
C	16.7680000000000	26.0689999999999	-0.3150000000000
O	15.5839999999999	26.1870000000000	-1.0980000000000
C	17.8049999999999	25.0899999999999	-0.8490000000000
O	18.1329999999999	25.2300000000000	-2.2220000000000
C	19.0189999999999	25.5459999999999	-0.0240000000000
N	19.1980000000000	24.7429999999999	1.2460000000000
C	18.7899999999999	25.0139999999999	2.5030000000000
N	19.0390000000000	24.0530000000000	3.3560000000000
C	19.6149999999999	23.0919999999999	2.6150000000000
C	20.0820000000000	21.7989999999999	2.9400000000000
N	20.0279999999999	21.2689999999999	4.2250000000000
N	20.5859999999999	21.0219999999999	1.9450000000000
C	20.6129999999999	21.5289999999999	0.7050000000000
N	20.2130000000000	22.7349999999999	0.2760000000000
C	19.7220000000000	23.4840000000000	1.2930000000000
H	17.6290000000000	29.3530000000000	0.5850000000000
H	16.0169999999999	28.8180000000000	0.1830000000000
H	17.7310000000000	27.7469999999999	-1.2770000000000

H	16.4579999999999	25.7989999999999	0.6970000000000
H	17.5479999999999	24.0599999999999	-0.6100000000000
H	18.6969999999999	24.4959999999999	-2.4790000000000
H	19.9570000000000	25.4710000000000	-0.5780000000000
H	18.2959999999999	25.9460000000000	2.7360000000000
H	20.3140000000000	20.3129999999999	4.3630000000000
H	19.6030000000000	21.8120000000000	4.9620000000000
H	21.0199999999999	20.8750000000000	-0.0530000000000
H	15.0589999999999	28.2429999999999	4.7360000000000
P	14.4090000000000	25.0479999999999	-1.0910000000000
O	13.1730000000000	25.4140000000000	-1.8350000000000
O	14.1750000000000	24.7500000000000	0.4870000000000
O	15.1440000000000	23.6920000000000	-1.6120000000000
C	15.4459999999999	23.5369999999999	-2.9870000000000
C	16.2010000000000	22.2169999999999	-3.2110000000000
O	17.2600000000000	22.0749999999999	-2.2710000000000
C	15.3940000000000	20.9600000000000	-2.9410000000000
O	14.5359999999999	20.6909999999999	-4.0460000000000
C	16.4770000000000	19.9089999999999	-2.7400000000000
O	17.1099999999999	19.4699999999999	-3.9330000000000
C	17.4950000000000	20.7289999999999	-1.9330000000000
N	17.3979999999999	20.5339999999999	-0.4310000000000
C	16.8670000000000	21.3290000000000	0.5220000000000
N	16.9929999999999	20.8520000000000	1.7360000000000
C	17.5990000000000	19.6670000000000	1.5660000000000
C	18.0300000000000	18.6799999999999	2.4840000000000
N	17.9130000000000	18.8069999999999	3.8650000000000
N	18.5869999999999	17.5440000000000	1.9920000000000
C	18.7139999999999	17.4259999999999	0.6640000000000
N	18.3859999999999	18.3000000000000	-0.2970000000000
C	17.8299999999999	19.4200000000000	0.2260000000000
H	16.0440000000000	24.3760000000000	-3.3370000000000
H	14.5320000000000	23.5509999999999	-3.5820000000000
H	16.6080000000000	22.1909999999999	-4.2240000000000
H	14.7910000000000	21.0889999999999	-2.0390000000000
H	16.1149999999999	19.0670000000000	-2.1520000000000
H	16.5030000000000	18.9039999999999	-4.4140000000000
H	18.5259999999999	20.4899999999999	-2.2020000000000
H	16.4130000000000	22.2740000000000	0.2650000000000
H	18.2469999999999	18.0560000000000	4.4540000000000
H	17.4649999999999	19.6260000000000	4.2500000000000
H	19.1600000000000	16.5060000000000	0.3190000000000
H	13.2539999999999	24.8430000000000	0.7950000000000
P	13.2040000000000	19.7540000000000	-3.9180000000000
O	12.4309999999999	19.5790000000000	-5.1780000000000
O	12.3729999999999	20.4439999999999	-2.7050000000000
O	13.7460000000000	18.3730000000000	-3.2500000000000
C	14.3590000000000	17.3850000000000	-4.0570000000000
C	14.7699999999999	16.1810000000000	-3.1970000000000

O	15.6300000000000	16.5850000000000	-2.1380000000000
C	13.6479999999999	15.4990000000000	-2.4340000000000
O	12.9659999999999	14.5869999999999	-3.2830000000000
C	14.3719999999999	14.7820000000000	-1.3080000000000
O	15.0169999999999	13.5809999999999	-1.7070000000000
C	15.4269999999999	15.8480000000000	-0.9570000000000
N	15.0099999999999	16.7940000000000	0.1550000000000
C	14.3140000000000	17.9480000000000	0.0720000000000
N	14.0890000000000	18.5199999999999	1.2290000000000
C	14.6470000000000	17.6810000000000	2.1140000000000
C	14.6999999999999	17.7899999999999	3.5560000000000
O	14.2460000000000	18.6759999999999	4.2760000000000
N	15.3789999999999	16.7100000000000	4.0600000000000
C	15.9179999999999	15.6470000000000	3.3620000000000
N	16.4860000000000	14.6920000000000	4.1010000000000
N	15.8710000000000	15.5579999999999	2.0750000000000
C	15.2219999999999	16.6060000000000	1.4910000000000
H	15.2300000000000	17.7940000000000	-4.5650000000000
H	13.6739999999999	17.0590000000000	-4.8420000000000
H	15.3040000000000	15.4540000000000	-3.8120000000000
H	12.9469999999999	16.2139999999999	-1.9960000000000
H	13.7110000000000	14.6020000000000	-0.4580000000000
H	14.3450000000000	12.9280000000000	-1.9200000000000
H	16.3810000000000	15.4060000000000	-0.6620000000000
H	13.9930000000000	18.3350000000000	-0.8800000000000
H	15.4849999999999	16.7049999999999	5.0660000000000
H	16.8539999999999	13.8610000000000	3.6560000000000
H	16.5740000000000	14.7880000000000	5.1050000000000
H	12.8550000000000	20.5609999999999	-1.8650000000000
P	11.4600000000000	14.8650000000000	-3.8460000000000
O	11.1809999999999	14.3190000000000	-5.2020000000000
O	11.2390000000000	16.4680000000000	-3.6950000000000
O	10.5389999999999	14.2439999999999	-2.6590000000000
C	10.5709999999999	12.8439999999999	-2.4400000000000
C	9.6120615798276	12.4478392896540	-1.3284774063472
O	10.0658084153658	12.9306201010262	-0.0602467798318
C	8.1746440465214	12.9509971280229	-1.4866578067842
O	7.3040249984229	11.9724004388712	-0.9303576922093
C	8.1316329043112	14.1901650192455	-0.5683270696792
O	6.8746097166865	14.5010264328853	-0.0619439610170
C	9.0680944161981	13.6980963096441	0.5592912815465
N	9.6779159316283	14.7585299598471	1.3260518927675
C	9.3095154051978	14.8945867706712	2.6489724425872
O	8.5057630850929	14.1134106743125	3.1943585665024
N	9.8740575519561	15.9459783261864	3.3058448068513
C	10.8490589972303	16.8115613972207	2.8114388061589
O	11.3657561737543	17.6405996711983	3.5650439661115
C	11.1608068579674	16.6254310632004	1.4103410536606
C	10.5855216985759	15.6109594785105	0.7332112516759

H	11.5809999999999	12.5269999999999	-2.1720000000000
H	10.3119999999999	12.3179999999999	-3.3610000000000
H	9.6085796886882	11.3537065368904	-1.3016025964176
H	7.9093688352053	13.1594515121775	-2.5220875039461
H	8.5900859713127	15.0374937804163	-1.0920328269856
H	6.3754912184826	15.0091559471388	-0.7247510529182
H	8.4691884556807	13.0869430848119	1.2591999360043
H	9.7118719406785	15.9902726496437	4.3212162208167
H	11.8887594772251	17.2975308513597	0.9726813465279
H	10.8087431163650	15.3813533632846	-0.3028383260485
H	11.0210000000000	16.9409999999999	-4.5220000000000
P	6.4609478805616	11.0290173534250	-1.8783757934204
O	5.8460839051674	9.9396438863675	-1.1050150045935
O	7.4618980172595	10.6336568910333	-3.0210123109894
O	5.4228452714017	11.9964631578053	-2.6315443599896
C	4.4279984429745	12.5738492178786	-1.7895679299716
C	3.7120000000000	13.6969999999999	-2.5520000000000
O	2.7850000000000	13.1560000000000	-3.4880000000000
C	2.8700000000000	14.5679999999999	-1.6110000000000
O	2.9310000000000	15.9339999999999	-2.0130000000000
C	1.4540000000000	14.0399999999999	-1.7750000000000
O	0.3970000000000	14.9369999999999	-1.4900000000000
C	1.4910000000000	13.6180000000000	-3.2460000000000
N	0.5140000000000	12.6300000000000	-3.6880000000000
C	-0.2950000000000	12.8970000000000	-4.7770000000000
O	-0.2950000000000	13.9209999999999	-5.4600000000000
N	-1.1690000000000	11.8859999999999	-5.0790000000000
C	-1.3350000000000	10.6669999999999	-4.4910000000000
O	-2.1820000000000	9.8600000000000	-4.8650000000000
C	-0.4120000000000	10.4380000000000	-3.3970000000000
C	0.4560000000000	11.4090000000000	-3.0540000000000
H	4.9103267135457	12.9823890905162	-0.8989786421122
H	3.7225012123577	11.7946218523000	-1.4920243908410
H	4.4600000000000	14.2870000000000	-3.0790000000000
H	3.1670000000000	14.5009999999999	-0.5640000000000
H	1.3450000000000	13.1539999999999	-1.1490000000000
H	0.7020000000000	15.8309999999999	-1.6650000000000
H	1.3790000000000	14.5099999999999	-3.8690000000000
H	-1.7830000000000	12.0730000000000	-5.8560000000000
H	-0.4230000000000	9.4900000000000	-2.8790000000000
H	1.1790000000000	11.2820000000000	-2.2610000000000
H	7.0815034581913	10.0707310549358	-3.7016823929825
P	4.1080000000000	16.9349999999999	-1.4750000000000
O	3.8990000000000	18.3750000000000	-1.7840000000000
O	5.4660000000000	16.3109999999999	-2.1080000000000
O	4.2390000000000	16.5740000000000	0.1080000000000
C	3.2380000000000	16.9980000000000	1.0170000000000
C	3.6165037905649	16.5593715909121	2.4332791668005
O	3.5782935090919	15.1357650339355	2.5129323180664

C	5.0201211215783	16.9993879710720	2.9264534488067
O	4.8927120342133	17.7005348534684	4.1491696150083
C	5.7526168456845	15.6591908643931	3.1768081067405
O	6.6299559208981	15.7051269209644	4.2543571155450
C	4.5408425536012	14.7436902239586	3.4650399702613
N	4.8040000000000	13.3270000000000	3.3760000000000
C	4.4650000000000	12.5229999999999	4.4500000000000
O	4.0570000000000	12.8970000000000	5.5470000000000
N	4.5980000000000	11.1809999999999	4.2080000000000
C	4.9620000000000	10.5320000000000	3.0660000000000
O	4.9720000000000	9.3080000000000	2.9710000000000
C	5.3060000000000	11.4380000000000	1.9870000000000
C	5.2060000000000	12.7650000000000	2.1860000000000
H	2.2730000000000	16.5650000000000	0.7480000000000
H	3.1240000000000	18.0809999999999	0.9600000000000
H	2.8756125109341	16.9801246106298	3.1265800997461
H	5.5290810313121	17.6279106402680	2.1920875105937
H	6.2455069149449	15.3425729517972	2.2522989958041
H	7.4022965006133	15.1237471751446	4.0536249440961
H	4.1847993321750	14.9339116868200	4.4903410520419
H	4.3650000000000	10.5809999999999	4.9850000000000
H	5.6220000000000	11.0370000000000	1.0360000000000
H	5.4170000000000	13.4809999999999	1.4100000000000
H	5.9340000000000	16.8999999999999	-2.7310000000000
P	5.9754777545963	18.8778233386723	4.4264322157638
O	5.8088549280075	20.0105837391231	3.5046505800847
O	7.3998690095771	18.2514723026152	4.4397235145145
O	5.7130039720558	19.2007218831501	5.9630899123082
C	4.6461944917924	20.0486317659953	6.3565547740412
C	4.2890000000000	19.7190000000000	7.8040000000000
O	3.8670000000000	18.3619999999999	7.8930000000000
C	5.4510000000000	19.7480000000000	8.7840000000000
O	5.7260000000000	21.0650000000000	9.2460000000000
C	4.9760000000000	18.8520000000000	9.9100000000000
O	4.0190000000000	19.4359999999999	10.7799999999999
C	4.3060000000000	17.7340000000000	9.0770000000000
N	5.2286921552471	16.6540614060618	8.7702037971766
C	5.7298369071779	15.9034116511862	9.8522845240384
O	5.4649399255355	16.2638525967564	11.0155548202270
N	6.4924915953288	14.8139019418722	9.5698490569104
C	6.6474689675098	14.4162411551632	8.2965271406190
N	7.3792205909456	13.3222062247244	8.0498025940608
C	6.1031160194983	15.1398141202737	7.1969691458796
C	5.3828238463304	16.2465854162388	7.4814511049353
H	3.7761849249506	19.8873010364649	5.7190201829749
H	4.9565166385668	21.0948724199681	6.2846673802394
H	3.4700000000000	20.3490000000000	8.1580000000000
H	6.3460000000000	19.3129999999999	8.3370000000000
H	5.8260000000000	18.4879999999999	10.4770000000000

H	4.4600000000000	20.0889999999999	11.3279999999999
H	3.4100000000000	17.3410000000000	9.5660000000000
H	7.4107168525501	12.9567809295031	7.1134629120263
H	7.7094572258964	12.7241248414076	8.7982961855640
H	6.1963884397105	14.7973992520748	6.1785457466162
H	4.8898107803950	16.8670374046488	6.7410255735350
H	7.3941043266984	17.2933543068790	4.6654703161990
P	7.1860000000000	21.7710000000000	9.0390000000000
O	7.2170000000000	23.2319999999999	9.3180000000000
O	7.6230000000000	21.3500000000000	7.5320000000000
O	8.1660000000000	20.8769999999999	9.9870000000000
C	7.9930000000000	20.9100000000000	11.3970000000000
C	8.5822107255628	19.5998472599485	11.9462636389489
O	7.9401049524442	18.5381217737036	11.2130738241645
C	10.0844332887050	19.2871326697639	11.7420649473708
O	10.9478349666327	19.8597501065572	12.6981949402895
C	10.0703473464338	17.7501269455538	11.8529774068043
O	10.1000240159838	17.3802597072865	13.2005805300713
C	8.7327810278116	17.3980868187209	11.1749049711451
N	9.0219839199988	16.9601626926508	9.8083970766376
C	9.5663263739710	15.6887430656843	9.7011043511909
O	9.8616613368009	15.0135216083570	10.6983512979186
N	9.7791462504668	15.2700734109664	8.4135467738293
C	9.4729393670458	15.9766455783622	7.2556813881108
O	9.6023679667499	15.4387010881849	6.1492607984428
C	9.0413195799776	17.3382004272896	7.4658014175755
C	8.8212779358462	17.7652442076016	8.7255771095984
H	6.9330000000000	20.9710000000000	11.6470000000000
H	8.4550000000000	21.8129999999999	11.7989999999999
H	8.3488409909367	19.5154558417362	13.0151771996891
H	10.3823195705153	19.5913984838550	10.7338391234191
H	10.8939983098634	17.2862150713947	11.3041426295302
H	10.1087650444499	16.4137012159703	13.2413274245669
H	8.2285252279201	16.5572778503087	11.6827741879121
H	10.1445511838929	14.3151111635485	8.3150762031633
H	8.9491249947962	17.9750052343484	6.5942451656002
H	8.5090515115979	18.7609816752914	9.0285432606997
H	7.5910000000000	20.3979999999999	7.3210000000000
P	12.5343365293408	19.5688408639171	12.6188819171940
O	13.2274028606309	20.3275382026702	13.6574417391931
O	12.9657280684602	19.8754085033165	11.1481601512531
O	12.8069114049474	17.9752410575361	12.7143611114199
C	13.0801482048850	17.3381125027952	13.9377311494372
C	13.8676019689017	16.0408752652636	13.6998638706878
O	13.1347065441598	15.1692382003403	12.7910769895389
C	15.2280369143322	16.2238383908727	13.0284158587649
O	16.2191718493812	16.5628059947810	14.0085004881293
C	15.4538760916490	14.8447342251181	12.4005910220183
O	15.7720733047049	13.8866697635112	13.4131699562344

C	14.0403733978472	14.5054485560659	11.9080114772613
N	13.7813014024000	15.0199103229541	10.5582182335972
C	13.2354077270311	16.2631168525627	10.3118327824181
N	13.0603294823590	16.5147981754102	9.0395413713151
C	13.5097777475922	15.3827958389445	8.3956396383496
C	13.5743365874182	15.0240347803567	7.0337412678877
N	13.1975868695977	15.8251770881990	6.0349265350666
N	14.0458298085717	13.7887111680483	6.7379730352572
C	14.4384725432542	12.9792215456467	7.7249737890825
N	14.4368442504539	13.2069135729614	9.0435095123297
C	13.9568668808250	14.4285377628949	9.3188278447025
H	12.1380812862575	17.0982408104732	14.4429556404996
H	13.6725856279317	17.9996574050562	14.5808886064409
H	14.0154576962938	15.5207557253491	14.6565037820139
H	15.1814702776335	16.9899921569563	12.2537540174825
H	16.2014155824199	14.8261343597505	11.5934923272954
H	16.4646236677776	14.3107428749473	13.9483169943744
H	13.8670277429325	13.4228432972084	11.9228334390547
H	12.9674320252098	16.8438926096626	11.1842466076250
H	13.1447887963243	15.4649034183997	5.0852510772631
H	12.8585228318299	16.7542433165232	6.2186403894295
H	14.7909521163199	11.9983356173712	7.4060961464058
H	12.8490291258463	19.1908090129438	10.4704074246474
P	17.3240648130546	17.7252936897528	13.7180900240093
O	18.0215265996929	18.0375484057026	14.9910202994824
O	16.5820869974529	18.8712428512145	12.9042509085038
O	18.2367116363388	17.1975792042501	12.5194784831100
C	19.2159396572863	16.1707912975690	12.8049353481532
C	19.5650000000000	15.5090000000000	11.4800000000000
O	18.3750000000000	15.1880000000000	10.7639999999999
C	20.2880000000000	16.3999999999999	10.4760000000000
O	21.6909999999999	16.5139999999999	10.6959999999999
C	20.0000000000000	15.7439999999999	9.1350000000000
O	20.8159999999999	14.6129999999999	8.8700000000000
C	18.5380000000000	15.3140000000000	9.3710000000000
N	17.5139999999999	16.1980000000000	8.8030000000000
C	17.0189999999999	15.9610000000000	7.5270000000000
O	17.3019999999999	14.9120000000000	6.9490000000000
N	16.2079999999999	16.9729999999999	6.9700000000000
C	15.9230000000000	18.0590000000000	7.5990000000000
N	15.1669999999999	19.0040000000000	7.0360000000000
C	16.4050000000000	18.3039999999999	8.9200000000000
C	17.1849999999999	17.3610000000000	9.4590000000000
H	18.7968084752679	15.4055614644593	13.4669747384212
H	20.0927553344532	16.6249928662104	13.2814558143472
H	20.1230000000000	14.5860000000000	11.6479999999999
H	19.8619999999999	17.4050000000000	10.4499999999999
H	20.0640000000000	16.4619999999999	8.3150000000000
H	21.7070000000000	14.9250000000000	8.6930000000000

H	18.3410000000000	14.3170000000000	8.9740000000000
H	14.9440000000000	19.8520000000000	7.5350000000000
H	14.8040000000000	18.8730000000000	6.0990000000000
H	16.1679999999999	19.1999999999999	9.4750000000000
H	17.5940000000000	17.4549999999999	10.4499999999999
H	15.9612850384310	19.4151469945841	13.4188708576749
P	22.3049999999999	17.7710000000000	11.5380000000000
O	21.5519999999999	19.0489999999999	11.4090000000000
O	23.8449999999999	17.8369999999999	11.0229999999999
O	22.4280000000000	17.1960000000000	13.0570000000000
C	23.3900000000000	16.2139999999999	13.4009999999999
C	22.7920000000000	15.2319999999999	14.4190000000000
O	21.9119999999999	14.3279999999999	13.7579999999999
C	23.8069999999999	14.2880000000000	15.0410000000000
O	24.5410000000000	14.9390000000000	16.0770000000000
C	22.9669999999999	13.0969999999999	15.4789999999999
O	22.2220000000000	13.3019999999999	16.6679999999999
C	21.9869999999999	13.0250000000000	14.2889999999999
N	22.2650000000000	12.0220000000000	13.2560000000000
C	22.1999999999999	10.6809999999999	13.6099999999999
O	21.8440000000000	10.3729999999999	14.7490000000000
N	22.5670000000000	9.7490000000000	12.6210000000000
C	23.0770000000000	10.0990000000000	11.4969999999999
N	23.4209999999999	9.1760000000000	10.5980000000000
C	23.2549999999999	11.4779999999999	11.1560000000000
C	22.8359999999999	12.3819999999999	12.0540000000000
H	24.2459999999999	16.7349999999999	13.8330000000000
H	23.7469999999999	15.6699999999999	12.5229999999999
H	22.2459999999999	15.7660000000000	15.1989999999999
H	24.5300000000000	13.9819999999999	14.2810000000000
H	23.5689999999999	12.1929999999999	15.5480000000000
H	22.8219999999999	13.2439999999999	17.4149999999999
H	20.9669999999999	12.7799999999999	14.6020000000000
H	23.7710000000000	9.4340000000000	9.6850000000000
H	23.2789999999999	8.1970000000000	10.8130000000000
H	23.6960000000000	11.7949999999999	10.2219999999999
H	22.9170000000000	13.4450000000000	11.8770000000000
H	24.3479999999999	17.0019999999999	11.0749999999999
P	25.7850000000000	14.2279999999999	16.8659999999999
O	26.5010000000000	15.0980000000000	17.8380000000000
O	26.7180000000000	13.6750000000000	15.6579999999999
O	25.1479999999999	12.8599999999999	17.4789999999999
C	24.7600000000000	12.7490000000000	18.8389999999999
C	24.9310000000000	11.2919999999999	19.3030000000000
O	24.2020000000000	10.4239999999999	18.4409999999999
C	26.3359999999999	10.7219999999999	19.1789999999999
O	27.1580000000000	11.1720000000000	20.2530000000000
C	26.0859999999999	9.2210000000000	19.1499999999999
O	25.7609999999999	8.6450000000000	20.4059999999999

C	24.8399999999999	9.1820000000000	18.2549999999999
N	25.1359999999999	8.9800000000000	16.7789999999999
C	25.3810000000000	9.8970000000000	15.8179999999999
N	25.5650000000000	9.3850000000000	14.6270000000000
C	25.4819999999999	8.0620000000000	14.8320000000000
C	25.5850000000000	6.9870000000000	13.8670000000000
O	25.7330000000000	7.0600000000000	12.6489999999999
N	25.4690000000000	5.7880000000000	14.5229999999999
C	25.2680000000000	5.5770000000000	15.8770000000000
N	25.1900000000000	4.3040000000000	16.2719999999999
N	25.1529999999999	6.5420000000000	16.7270000000000
C	25.2600000000000	7.7740000000000	16.1499999999999
H	23.7229999999999	13.0640000000000	18.9490000000000
H	25.3520000000000	13.4019999999999	19.4830000000000
H	24.5590000000000	11.1760000000000	20.3219999999999
H	26.7890000000000	11.0589999999999	18.2439999999999
H	26.8990000000000	8.6790000000000	18.6759999999999
H	26.5749999999999	8.5070000000000	20.8960000000000
H	24.1430000000000	8.3940000000000	18.5479999999999
H	25.3939999999999	10.9489999999999	16.0489999999999
H	25.5270000000000	4.9720000000000	13.9269999999999
H	25.0390000000000	4.0910000000000	17.2459999999999
H	25.2920000000000	3.5440000000000	15.6099999999999
H	27.6690000000000	13.8770000000000	15.7420000000000
P	28.7869999999999	11.0259999999999	20.2429999999999
O	29.4890000000000	11.7430000000000	21.3410000000000
O	29.2040000000000	11.5160000000000	18.7530000000000
O	29.0579999999999	9.4220000000000	20.1890000000000
C	29.0210000000000	8.6460000000000	21.3730000000000
C	29.1589999999999	7.1510000000000	21.0360000000000
O	28.1900000000000	6.8000000000000	20.0500000000000
C	30.4430000000000	6.7230000000000	20.3389999999999
O	31.5489999999999	6.6270000000000	21.2330000000000
C	30.0330000000000	5.4080000000000	19.6859999999999
O	29.8590000000000	4.3140000000000	20.5730000000000
C	28.6600000000000	5.8230000000000	19.1520000000000
N	28.7160000000000	6.3850000000000	17.7429999999999
C	28.9250000000000	7.6520000000000	17.3219999999999
N	28.9690000000000	7.7800000000000	16.0199999999999
C	28.8449999999999	6.5240000000000	15.5739999999999
C	28.8719999999999	6.0450000000000	14.2110000000000
O	28.9789999999999	6.6980000000000	13.1760000000000
N	28.7450000000000	4.6790000000000	14.2159999999999
C	28.6329999999999	3.8410000000000	15.3109999999999
N	28.5899999999999	2.5320000000000	15.0500000000000
N	28.5889999999999	4.2780000000000	16.5240000000000
C	28.6980000000000	5.6370000000000	16.6039999999999
H	28.0799999999999	8.8200000000000	21.8979999999999
H	29.8170000000000	8.9540000000000	22.0530000000000

H	28.9989999999999	6.5510000000000	21.9329999999999
H	30.7130000000000	7.4680000000000	19.5859999999999
H	30.6859999999999	5.1360000000000	18.8619999999999
H	29.7349999999999	3.5150000000000	20.0530000000000
H	27.9469999999999	4.9950000000000	19.1370000000000
H	29.0420000000000	8.4620000000000	18.0210000000000
H	28.7530000000000	4.2550000000000	13.2970000000000
H	28.4959999999999	1.8720000000000	15.8059999999999
H	28.6769999999999	2.1890000000000	14.1010000000000
H	29.8780000000000	12.2219999999999	18.7210000000000
P	33.0840000000000	6.3980000000000	20.7109999999999
O	34.1300000000000	6.4880000000000	21.7659999999999
O	33.2449999999999	7.4900000000000	19.5199999999999
O	33.0480000000000	4.9870000000000	19.8990000000000
C	33.0949999999999	3.7540000000000	20.5949999999999
C	33.0769999999999	2.5820000000000	19.5979999999999
O	31.9899999999999	2.7210000000000	18.6840000000000
C	34.2610000000000	2.5060000000000	18.6449999999999
O	35.3879999999999	1.9190000000000	19.2860000000000
C	33.7130000000000	1.6430000000000	17.5169999999999
O	33.6049999999999	0.2600000000000	17.8150000000000
C	32.3059999999999	2.2380000000000	17.3979999999999
N	32.2040000000000	3.3600000000000	16.3820000000000
C	32.2670000000000	4.6970000000000	16.5659999999999
N	32.1649999999999	5.3910000000000	15.4610000000000
C	32.0889999999999	4.4580000000000	14.5039999999999
C	32.0060000000000	4.6380000000000	13.0709999999999
O	31.9149999999999	5.6860000000000	12.4390000000000
N	32.0720000000000	3.4120000000000	12.4619999999999
C	32.1659999999999	2.1720000000000	13.0649999999999
N	32.2770000000000	1.1300000000000	12.2370000000000
N	32.1619999999999	2.0120000000000	14.3460000000000
C	32.1370000000000	3.1960000000000	15.0280000000000
H	32.2509999999999	3.6800000000000	21.2789999999999
H	33.9939999999999	3.7010000000000	21.2119999999999
H	32.9690000000000	1.6410000000000	20.1389999999999
H	34.5230000000000	3.5060000000000	18.2920000000000
H	34.2640000000000	1.7990000000000	16.5930000000000
H	34.4859999999999	-0.1210000000000	17.8350000000000
H	31.5609999999999	1.4910000000000	17.1140000000000
H	32.3950000000000	5.1190000000000	17.5489999999999
H	32.0730000000000	3.4400000000000	11.4510000000000
H	32.3380000000000	0.1940000000000	12.6129999999999
H	32.3149999999999	1.2640000000000	11.2370000000000
H	34.0020000000000	8.0990000000000	19.6140000000000
P	36.9209999999999	2.2510000000000	18.8230000000000
O	37.9799999999999	1.6030000000000	19.6419999999999
O	36.9699999999999	3.8730000000000	18.8210000000000
O	36.9519999999999	1.8800000000000	17.2379999999999

C	37.0439999999999	0.5310000000000	16.8140000000000
C	36.9620000000000	0.4340000000000	15.2799999999999
O	35.7610000000000	1.0410000000000	14.8079999999999
C	38.0170000000000	1.2100000000000	14.5009999999999
O	39.2710000000000	0.5330000000000	14.5109999999999
C	37.3780000000000	1.3420000000000	13.1229999999999
O	37.3470000000000	0.1580000000000	12.3450000000000
C	35.9419999999999	1.6740000000000	13.5609999999999
N	35.5870000000000	3.0960000000000	13.5999999999999
C	35.4579999999999	3.7770000000000	12.3979999999999
O	35.5349999999999	3.1500000000000	11.3390000000000
N	35.2460000000000	5.1680000000000	12.4809999999999
C	35.2869999999999	5.8100000000000	13.5939999999999
N	35.0619999999999	7.1240000000000	13.6359999999999
C	35.5369999999999	5.1380000000000	14.8300000000000
C	35.6689999999999	3.8060000000000	14.7759999999999
H	36.2419999999999	-0.0550000000000	17.2630000000000
H	37.9799999999999	0.0940000000000	17.1660000000000
H	36.9660000000000	-0.6140000000000	14.9800000000000
H	38.1610000000000	2.1890000000000	14.9610000000000
H	37.8070000000000	2.1620000000000	12.5559999999999
H	38.2190000000000	0.0140000000000	11.9700000000000
H	35.1869999999999	1.2330000000000	12.9049999999999
H	35.0559999999999	7.6200000000000	14.5139999999999
H	34.8359999999999	7.6200000000000	12.7829999999999
H	35.5989999999999	5.6560000000000	15.7759999999999
H	35.8389999999999	3.2170000000000	15.6590000000000
H	37.6629999999999	4.2670000000000	19.3840000000000
P	40.6730000000000	1.2700000000000	14.1020000000000
O	41.9020000000000	0.4720000000000	14.3640000000000
O	40.6109999999999	2.6820000000000	14.8989999999999
O	40.4680000000000	1.7190000000000	12.5510000000000
C	40.5720000000000	0.7630000000000	11.5079999999999
C	40.2559999999999	1.4290000000000	10.1590000000000
O	39.0399999999999	2.1600000000000	10.2639999999999
C	41.2349999999999	2.5020000000000	9.7080000000000
O	42.3539999999999	1.8930000000000	9.0660000000000
C	40.3950000000000	3.3560000000000	8.7670000000000
O	40.1910000000000	2.8010000000000	7.4770000000000
C	39.0539999999999	3.3450000000000	9.5080000000000
N	38.8149999999999	4.5440000000000	10.4039999999999
C	38.8789999999999	4.6490000000000	11.7479999999999
N	38.5090000000000	5.8160000000000	12.2059999999999
C	38.2599999999999	6.5340000000000	11.1010000000000
C	37.8059999999999	7.8580000000000	10.9230000000000
N	37.4290000000000	8.6720000000000	11.9819999999999
N	37.7120000000000	8.3480000000000	9.6600000000000
C	38.0290000000000	7.5320000000000	8.6460000000000
N	38.4110000000000	6.2460000000000	8.6840000000000

C	38.5020000000000	5.7950000000000	9.9600000000000
H	39.8819999999999	-0.0590000000000	11.6899999999999
H	41.5730000000000	0.3280000000000	11.4890000000000
H	40.1490000000000	0.6700000000000	9.3820000000000
H	41.5880000000000	3.0800000000000	10.5649999999999
H	40.7719999999999	4.3700000000000	8.7020000000000
H	39.7490000000000	3.4550000000000	6.9280000000000
H	38.2060000000000	3.3120000000000	8.8210000000000
H	39.1739999999999	3.8120000000000	12.3599999999999
H	37.0469999999999	9.5900000000000	11.7880000000000
H	37.4930000000000	8.3170000000000	12.9260000000000
H	37.9350000000000	7.9610000000000	7.6600000000000
H	41.3440000000000	2.8380000000000	15.5239999999999
P	43.7689999999999	2.6690000000000	8.8040000000000
O	44.7619999999999	1.9120000000000	7.9940000000000
O	44.2649999999999	3.0340000000000	10.3059999999999
O	43.3509999999999	4.1240000000000	8.2050000000000
C	43.1480000000000	4.3300000000000	6.8180000000000
C	42.7859999999999	5.8060000000000	6.5610000000000
O	41.6559999999999	6.1890000000000	7.3400000000000
C	43.8299999999999	6.8140000000000	7.0140000000000
O	44.8939999999999	6.8670000000000	6.0670000000000
C	43.0319999999999	8.1040000000000	7.1860000000000
O	42.6880000000000	8.7660000000000	5.9810000000000
C	41.7419999999999	7.5220000000000	7.7920000000000
N	41.5970000000000	7.6140000000000	9.2460000000000
C	41.2019999999999	8.8160000000000	9.8150000000000
O	41.0469999999999	9.8020000000000	9.0940000000000
N	41.0170000000000	8.8110000000000	11.2159999999999
C	41.3750000000000	7.8210000000000	11.9550000000000
N	41.1989999999999	7.8430000000000	13.2780000000000
C	41.9519999999999	6.6410000000000	11.3919999999999
C	42.0349999999999	6.5930000000000	10.0559999999999
H	42.3619999999999	3.6770000000000	6.4460000000000
H	44.0510000000000	4.0690000000000	6.2650000000000
H	42.5510000000000	5.9550000000000	5.5050000000000
H	44.2529999999999	6.4980000000000	7.9690000000000
H	43.5129999999999	8.7830000000000	7.8850000000000
H	42.3200000000000	9.6270000000000	6.2000000000000
H	40.8419999999999	8.0070000000000	7.4000000000000
H	41.4629999999999	7.0550000000000	13.8469999999999
H	40.7599999999999	8.6420000000000	13.7189999999999
H	42.2999999999999	5.8150000000000	11.9939999999999
H	42.4440000000000	5.7480000000000	9.5390000000000
H	43.6319999999999	3.5320000000000	10.8550000000000
P	46.3269999999999	7.5880000000000	6.3790000000000
O	47.3819999999999	7.3680000000000	5.3510000000000
O	46.7100000000000	7.0460000000000	7.8610000000000
O	45.9489999999999	9.1450000000000	6.6480000000000

C	45.6700000000000	10.0180000000000	5.5720000000000
C	45.2559999999999	11.3930000000000	6.1180000000000
O	44.1589999999999	11.2720000000000	7.0200000000000
C	46.2929999999999	12.1140000000000	6.9730000000000
O	47.2120000000000	12.8109999999999	6.1430000000000
C	45.4609999999999	13.0879999999999	7.7900000000000
O	45.0949999999999	14.2430000000000	7.0600000000000
C	44.2199999999999	12.2189999999999	8.0610000000000
N	44.1689999999999	11.5570000000000	9.3670000000000
C	43.5540000000000	12.1799999999999	10.4440000000000
O	43.1689999999999	13.3450000000000	10.3300000000000
N	43.4470000000000	11.4139999999999	11.6259999999999
C	44.0439999999999	10.2840000000000	11.7720000000000
N	43.9750000000000	9.6050000000000	12.9160000000000
C	44.8029999999999	9.6990000000000	10.7159999999999
C	44.8370000000000	10.3710000000000	9.5600000000000
H	44.8800000000000	9.6060000000000	4.9450000000000
H	46.5459999999999	10.1170000000000	4.9300000000000
H	44.9470000000000	12.0350000000000	5.2900000000000
H	46.8250000000000	11.4350000000000	7.6410000000000
H	45.9420000000000	13.3640000000000	8.7270000000000
H	44.5439999999999	14.8100000000000	7.6090000000000
H	43.2950000000000	12.7970000000000	7.9810000000000
H	44.4680000000000	8.7330000000000	13.0329999999999
H	43.4269999999999	9.9760000000000	13.6839999999999
H	45.3400000000000	8.7680000000000	10.8219999999999
H	45.3900000000000	10.0180000000000	8.7100000000000
H	47.5900000000000	6.6280000000000	7.9340000000000
P	48.7909999999999	12.4190000000000	6.0630000000000
O	49.5039999999999	12.9489999999999	4.8700000000000
O	48.8130000000000	10.8010000000000	6.2130000000000
O	49.3499999999999	12.9410000000000	7.5020000000000
C	50.7400000000000	13.1470000000000	7.6830000000000
C	50.9650000000000	13.9770000000000	8.9570000000000
O	50.6289999999999	13.1829999999999	10.0909999999999
C	52.4249999999999	14.3469999999999	9.2200000000000
O	52.4159999999999	15.4710000000000	10.0920000000000
C	52.9969999999999	13.1460000000000	9.9530000000000
O	54.1169999999999	13.4130000000000	10.7780000000000
C	51.7689999999999	12.7159999999999	10.7530000000000
N	51.6520000000000	11.2460000000000	11.0990000000000
C	51.9420000000000	10.1709999999999	10.3339999999999
N	51.7800000000000	9.0250000000000	10.9510000000000
C	51.3659999999999	9.3720000000000	12.1769999999999
C	51.0379999999999	8.5190000000000	13.3019999999999
O	51.0810000000000	7.2930000000000	13.3750000000000
N	50.6319999999999	9.3010000000000	14.3539999999999
C	50.5390000000000	10.6809999999999	14.3940000000000
N	50.0709999999999	11.2010000000000	15.5280000000000

N	50.8619999999999	11.4369999999999	13.4000000000000
C	51.2659999999999	10.7319999999999	12.3030000000000
H	51.1409999999999	13.6929999999999	6.8260000000000
H	51.2490000000000	12.1829999999999	7.7320000000000
H	50.3380000000000	14.8699999999999	8.9180000000000
H	52.9779999999999	14.5730000000000	8.3070000000000
H	53.2449999999999	12.3699999999999	9.2290000000000
H	54.1180000000000	14.3499999999999	10.9949999999999
H	51.7680000000000	13.2520000000000	11.7040000000000
H	52.2779999999999	10.2859999999999	9.3140000000000
H	50.3599999999999	8.7900000000000	15.1790000000000
H	49.9279999999999	12.1999999999999	15.5939999999999
H	49.7280000000000	10.6069999999999	16.2689999999999
H	49.2430000000000	10.3200000000000	5.4810000000000
P	53.1390000000000	16.8739999999999	9.6890000000000
O	53.5159999999999	17.0470000000000	8.2590000000000
O	54.3800000000000	16.9379999999999	10.7309999999999
O	52.1129999999999	17.9860000000000	10.2810000000000
C	51.1009999999999	18.5450000000000	9.4620000000000
C	50.0660000000000	19.2639999999999	10.3450000000000
O	49.2959999999999	18.2800000000000	11.0280000000000
C	50.6820000000000	20.1129999999999	11.4640000000000
O	49.9229999999999	21.3069999999999	11.6400000000000
C	50.5989999999999	19.2310000000000	12.7040000000000
O	50.6089999999999	19.9009999999999	13.9510000000000
C	49.2989999999999	18.4810000000000	12.4130000000000
N	49.0849999999999	17.2289999999999	13.1310000000000
C	48.6129999999999	17.2940000000000	14.4280000000000
O	48.2589999999999	18.3090000000000	15.0280000000000
N	48.5540000000000	16.0829999999999	15.0619999999999
C	48.9440000000000	14.8510000000000	14.6319999999999
O	48.8489999999999	13.8520000000000	15.3399999999999
C	49.4750000000000	14.8650000000000	13.2820000000000
C	49.5309999999999	16.0309999999999	12.6080000000000
H	50.6109999999999	17.7789999999999	8.8560000000000
H	51.5579999999999	19.2530000000000	8.7700000000000
H	49.3969999999999	19.8479999999999	9.7150000000000
H	51.7209999999999	20.3880000000000	11.2780000000000
H	51.4099999999999	18.5010000000000	12.6869999999999
H	51.4829999999999	20.2699999999999	14.0960000000000
H	48.4209999999999	19.0979999999999	12.6259999999999
H	48.1820000000000	16.1090000000000	16.0000000000000
H	49.8139999999999	13.9450000000000	12.8339999999999
H	49.9200000000000	16.0919999999999	11.6010000000000
H	55.2429999999999	17.1720000000000	10.3369999999999
P	50.3810000000000	22.6870000000000	10.8960000000000
O	51.8489999999999	22.9310000000000	10.8559999999999
O	49.5319999999999	23.8270000000000	11.6799999999999
O	49.6540000000000	22.5940000000000	9.4380000000000

C	48.2499999999999	22.7480000000000	9.3440000000000
C	47.7219999999999	22.2210000000000	7.9990000000000
O	47.8639999999999	20.8069999999999	7.9650000000000
C	46.2219999999999	22.4649999999999	7.8210000000000
O	46.0360000000000	23.6600000000000	7.0630000000000
C	45.6540000000000	21.1879999999999	7.2040000000000
O	45.7409999999999	21.1239999999999	5.7910000000000
C	46.6370000000000	20.1700000000000	7.7980000000000
N	46.2520000000000	19.4439999999999	9.0760000000000
C	45.6180000000000	19.9130000000000	10.1709999999999
N	45.4789999999999	19.0249999999999	11.1229999999999
C	46.0309999999999	17.9100000000000	10.6210000000000
C	46.1799999999999	16.6090000000000	11.1519999999999
N	45.7109999999999	16.2379999999999	12.4079999999999
N	46.7959999999999	15.6639999999999	10.3930000000000
C	47.2329999999999	16.0249999999999	9.1790000000000
N	47.1490000000000	17.2149999999999	8.5680000000000
C	46.5249999999999	18.1320000000000	9.3480000000000
H	48.0200000000000	23.8109999999999	9.4410000000000
H	47.7560000000000	22.2280000000000	10.1669999999999
H	48.2869999999999	22.6439999999999	7.1670000000000
H	45.7649999999999	22.6340000000000	8.7970000000000
H	44.6420000000000	20.9819999999999	7.5460000000000
H	45.0050000000000	21.6179999999999	5.4200000000000
H	46.8170000000000	19.3780000000000	7.0690000000000
H	45.2809999999999	20.9370000000000	10.2260000000000
H	45.8659999999999	15.2889999999999	12.7230000000000
H	45.3689999999999	16.9520000000000	13.0389999999999
H	47.7190000000000	15.2449999999999	8.6100000000000
H	50.0649999999999	24.5440000000000	12.0719999999999
P	44.6069999999999	24.4480000000000	6.9670000000000
O	44.6610000000000	25.7699999999999	6.2890000000000
O	44.1030000000000	24.5049999999999	8.5090000000000
O	43.5970000000000	23.3659999999999	6.2950000000000
C	43.4560000000000	23.2800000000000	4.8870000000000
C	42.3440000000000	22.2719999999999	4.5420000000000
O	42.6179999999999	21.0519999999999	5.2350000000000
C	40.9380000000000	22.6539999999999	5.0230000000000
O	40.0009999999999	21.9710000000000	4.1960000000000
C	40.8419999999999	22.0410000000000	6.4080000000000
O	39.5260000000000	21.8520000000000	6.9020000000000
C	41.5720000000000	20.7330000000000	6.1090000000000
N	42.0309999999999	19.9080000000000	7.2850000000000
C	42.1779999999999	20.2579999999999	8.5780000000000
N	42.5200000000000	19.2600000000000	9.3500000000000
C	42.6420000000000	18.2169999999999	8.5200000000000
C	42.9390000000000	16.8569999999999	8.7520000000000
N	43.1549999999999	16.3219999999999	10.0150000000000
N	42.9819999999999	16.0189999999999	7.6880000000000

C	42.7610000000000	16.5309999999999	6.4700000000000
N	42.4540000000000	17.7890000000000	6.1260000000000
C	42.3950000000000	18.5949999999999	7.2140000000000
H	44.3999999999999	22.9710000000000	4.4390000000000
H	43.2180000000000	24.2560000000000	4.4610000000000
H	42.3440000000000	22.1039999999999	3.4640000000000
H	40.7509999999999	23.7300000000000	5.0090000000000
H	41.4229999999999	22.6209999999999	7.1230000000000
H	39.5750000000000	21.6239999999999	7.8340000000000
H	40.9119999999999	20.0659999999999	5.5520000000000
H	41.9799999999999	21.2650000000000	8.9150000000000
H	43.2909999999999	15.3239999999999	10.1039999999999
H	43.0630000000000	16.9170000000000	10.8300000000000
H	42.8109999999999	15.8239999999999	5.6530000000000
H	43.8800000000000	25.3969999999999	8.8390000000000
P	38.8509999999999	22.7650000000000	3.3660000000000
O	39.3389999999999	23.5139999999999	2.1760000000000
O	38.1300000000000	23.6720000000000	4.5040000000000
O	37.7530000000000	21.5990000000000	3.0570000000000
C	37.2060000000000	20.7830000000000	4.0870000000000
C	37.6079999999999	19.3150000000000	3.8530000000000
O	38.8689999999999	19.0120000000000	4.4550000000000
C	36.7559999999999	18.2469999999999	4.5250000000000
O	35.4870000000000	18.0229999999999	3.9260000000000
C	37.6439999999999	17.0219999999999	4.3930000000000
O	37.7800000000000	16.4890000000000	3.0850000000000
C	38.9579999999999	17.6559999999999	4.8510000000000
N	39.1450000000000	17.5459999999999	6.3560000000000
C	38.9169999999999	18.4639999999999	7.3170000000000
N	39.1009999999999	18.0159999999999	8.5340000000000
C	39.4139999999999	16.7220000000000	8.3640000000000
C	39.6869999999999	15.6850000000000	9.2870000000000
N	39.6920000000000	15.8680000000000	10.6649999999999
N	39.9500000000000	14.4429999999999	8.8030000000000
C	39.9399999999999	14.2669999999999	7.4760000000000
N	39.6980000000000	15.1630000000000	6.5070000000000
C	39.4450000000000	16.3919999999999	7.0200000000000
H	36.1250000000000	20.8850000000000	4.0070000000000
H	37.4889999999999	21.1149999999999	5.0870000000000
H	37.6579999999999	19.1239999999999	2.7790000000000
H	36.6090000000000	18.4909999999999	5.5810000000000
H	37.3509999999999	16.2379999999999	5.0850000000000
H	36.9709999999999	16.0219999999999	2.8590000000000
H	39.8299999999999	17.1960000000000	4.3830000000000
H	38.6019999999999	19.4619999999999	7.0550000000000
H	39.8629999999999	15.0730000000000	11.2660000000000
H	39.4969999999999	16.7850000000000	11.0459999999999
H	40.1510000000000	13.2629999999999	7.1390000000000
H	37.8969999999999	23.2040000000000	5.3290000000000

P	34.1139999999999	18.2680000000000	4.7770000000000
O	32.8509999999999	18.0749999999999	4.0150000000000
O	34.3070000000000	19.7570000000000	5.3930000000000
O	34.3040000000000	17.3090000000000	6.0860000000000
C	33.7920000000000	15.9849999999999	6.1080000000000
C	34.7589999999999	14.9830000000000	5.4500000000000
O	35.9040000000000	14.7769999999999	6.2700000000000
C	34.2070000000000	13.5719999999999	5.3200000000000
O	33.3719999999999	13.5169999999999	4.1690000000000
C	35.4579999999999	12.7080000000000	5.2310000000000
O	36.1210000000000	12.7409999999999	3.9760000000000
C	36.3290000000000	13.4339999999999	6.2780000000000
N	36.3430000000000	12.8949999999999	7.6380000000000
C	36.9590000000000	11.6730000000000	7.8440000000000
O	37.5709999999999	11.0210000000000	6.9980000000000
N	36.8290000000000	11.1910000000000	9.1200000000000
C	36.1480000000000	11.7029999999999	10.1850000000000
O	36.1330000000000	11.1739999999999	11.2929999999999
C	35.4569999999999	12.9350000000000	9.8680000000000
C	35.5810000000000	13.4640000000000	8.6380000000000
H	32.8190000000000	15.9550000000000	5.6140000000000
H	33.6199999999999	15.7100000000000	7.1480000000000
H	35.0810000000000	15.3469999999999	4.4730000000000
H	33.6270000000000	13.3230000000000	6.2130000000000
H	35.2659999999999	11.6809999999999	5.5300000000000
H	35.6300000000000	12.1950000000000	3.3570000000000
H	37.3870000000000	13.4469999999999	5.9980000000000
H	37.2939999999999	10.3100000000000	9.2930000000000
H	34.8439999999999	13.4109999999999	10.6159999999999
H	35.0840000000000	14.3840000000000	8.3750000000000
H	33.5640000000000	20.3709999999999	5.2320000000000
P	32.0820000000000	12.5220000000000	4.0630000000000
O	31.1619999999999	12.7989999999999	2.9280000000000
O	31.3900000000000	12.6150000000000	5.5290000000000
O	32.7490000000000	11.0399999999999	4.0600000000000
C	33.4149999999999	10.5779999999999	2.8970000000000
C	34.0409999999999	9.2030000000000	3.1640000000000
O	34.9239999999999	9.2990000000000	4.2730000000000
C	33.0739999999999	8.1240000000000	3.6230000000000
O	32.3969999999999	7.5720000000000	2.4980000000000
C	33.9859999999999	7.1450000000000	4.3500000000000
O	34.7659999999999	6.3120000000000	3.5070000000000
C	34.9260000000000	8.1350000000000	5.0580000000000
N	34.5330000000000	8.4730000000000	6.4830000000000
C	33.9449999999999	9.5790000000000	6.9880000000000
N	33.7899999999999	9.5490000000000	8.2890000000000
C	34.2520000000000	8.3400000000000	8.6410000000000
C	34.3230000000000	7.7370000000000	9.9560000000000
O	34.0539999999999	8.2360000000000	11.0459999999999

N	34.7640000000000	6.4430000000000	9.8280000000000
C	35.1379999999999	5.7850000000000	8.6690000000000
N	35.5060000000000	4.5100000000000	8.7980000000000
N	35.1379999999999	6.3540000000000	7.5110000000000
C	34.6779999999999	7.6370000000000	7.5490000000000
H	34.1880000000000	11.2859999999999	2.6010000000000
H	32.7130000000000	10.5139999999999	2.0620000000000
H	34.6049999999999	8.8690000000000	2.2910000000000
H	32.3340000000000	8.5470000000000	4.3070000000000
H	33.4429999999999	6.5550000000000	5.0830000000000
H	35.2150000000000	5.6600000000000	4.0510000000000
H	35.9510000000000	7.7620000000000	5.1090000000000
H	33.6619999999999	10.4000000000000	6.3470000000000
H	34.8329999999999	5.9330000000000	10.6999999999999
H	35.7909999999999	3.9850000000000	7.9860000000000
H	35.4939999999999	4.0500000000000	9.7020000000000
H	30.4340000000000	12.8130000000000	5.5260000000000
P	30.9869999999999	6.7560000000000	2.6280000000000
O	30.3769999999999	6.3330000000000	1.3380000000000
O	30.0560000000000	7.7500000000000	3.5120000000000
O	31.3099999999999	5.5460000000000	3.6650000000000
C	31.9909999999999	4.3780000000000	3.2430000000000
C	32.1809999999999	3.4340000000000	4.4430000000000
O	32.8530000000000	4.1060000000000	5.5060000000000
C	30.8949999999999	2.9910000000000	5.1230000000000
O	30.2650000000000	1.9690000000000	4.3560000000000
C	31.3539999999999	2.5830000000000	6.5170000000000
O	32.0150000000000	1.3310000000000	6.5940000000000
C	32.3890000000000	3.6940000000000	6.7710000000000
N	31.9409999999999	4.8290000000000	7.5780000000000
C	31.8679999999999	4.6560000000000	8.9470000000000
O	32.1749999999999	3.6340000000000	9.5620000000000
N	31.4089999999999	5.7570000000000	9.6190000000000
C	30.9549999999999	6.9520000000000	9.1430000000000
O	30.5919999999999	7.8690000000000	9.8740000000000
C	30.9520000000000	7.0130000000000	7.6960000000000
C	31.4289999999999	5.9670000000000	6.9960000000000
H	32.9540000000000	4.6360000000000	2.8060000000000
H	31.4220000000000	3.8750000000000	2.4590000000000
H	32.7730000000000	2.5660000000000	4.1460000000000
H	30.2049999999999	3.8350000000000	5.1810000000000
H	30.5470000000000	2.6430000000000	7.2410000000000
H	31.3509999999999	0.6390000000000	6.6310000000000
H	33.2760000000000	3.3230000000000	7.2940000000000
H	31.4089999999999	5.6810000000000	10.6280000000000
H	30.5700000000000	7.8910000000000	7.1980000000000
H	31.4420000000000	5.9690000000000	5.9230000000000
H	29.2300000000000	8.0380000000000	3.0790000000000
P	28.7190000000000	1.4960000000000	4.6030000000000

O	28.1550000000000	0.6100000000000	3.5510000000000
O	27.9190000000000	2.8940000000000	4.8060000000000
O	28.7270000000000	0.8620000000000	6.1000000000000
C	29.2160000000000	-0.4470000000000	6.3250000000000
C	29.1969999999999	-0.7460000000000	7.8330000000000
O	29.8919999999999	0.2750000000000	8.5400000000000
C	27.8270000000000	-0.7070000000000	8.4980000000000
O	27.1400000000000	-1.9300000000000	8.2510000000000
C	28.1709999999999	-0.4920000000000	9.9650000000000
O	28.6200000000000	-1.6460000000000	10.6579999999999
C	29.3339999999999	0.5010000000000	9.8120000000000
N	29.0049999999999	1.9140000000000	10.0069999999999
C	28.9570000000000	2.4300000000000	11.2919999999999
O	29.0530000000000	1.6670000000000	12.2550000000000
N	28.7579999999999	3.8200000000000	11.3910000000000
C	28.3659999999999	4.5330000000000	10.3949999999999
N	28.1200000000000	5.8350000000000	10.5389999999999
C	28.1999999999999	3.9630000000000	9.0950000000000
C	28.5309999999999	2.6720000000000	8.9610000000000
H	30.2289999999999	-0.5420000000000	5.9350000000000
H	28.6060000000000	-1.1760000000000	5.7890000000000
H	29.6829999999999	-1.7050000000000	8.0280000000000
H	27.2480000000000	0.1340000000000	8.1130000000000
H	27.3539999999999	-0.0180000000000	10.4960000000000
H	27.8560000000000	-2.1840000000000	10.8789999999999
H	30.1449999999999	0.2980000000000	10.5190000000000
H	27.8170000000000	6.4010000000000	9.7610000000000
H	28.2600000000000	6.2720000000000	11.4429999999999
H	27.8339999999999	4.5260000000000	8.2490000000000
H	28.4450000000000	2.1480000000000	8.0250000000000
H	27.1709999999999	3.0350000000000	4.1950000000000
P	25.5279999999999	-2.0100000000000	7.9890000000000
O	25.0689999999999	-3.2460000000000	7.2990000000000
O	25.1720000000000	-0.6310000000000	7.2070000000000
O	24.8889999999999	-1.7810000000000	9.4690000000000
C	25.0139999999999	-2.7980000000000	10.4499999999999
C	24.8949999999999	-2.2180000000000	11.8680000000000
O	25.8440000000000	-1.1680000000000	12.0280000000000
C	23.6000000000000	-1.4930000000000	12.2059999999999
O	22.5369999999999	-2.4060000000000	12.4589999999999
C	24.0010000000000	-0.6250000000000	13.3989999999999
O	24.1769999999999	-1.3180000000000	14.6240000000000
C	25.3870000000000	-0.1730000000000	12.9120000000000
N	25.4450000000000	1.1620000000000	12.3160000000000
C	25.4780000000000	2.2670000000000	13.1539999999999
O	25.4810000000000	2.0990000000000	14.3750000000000
N	25.4639999999999	3.5280000000000	12.5250000000000
C	25.1879999999999	3.6670000000000	11.2769999999999
N	25.0820000000000	4.8780000000000	10.7270000000000

C	25.0010000000000	2.5370000000000	10.4220000000000
C	25.1430000000000	1.3300000000000	10.9830000000000
H	25.9819999999999	-3.2930000000000	10.3559999999999
H	24.2579999999999	-3.5660000000000	10.2850000000000
H	25.0930000000000	-2.9980000000000	12.6059999999999
H	23.2959999999999	-0.8760000000000	11.3580000000000
H	23.3430000000000	0.2320000000000	13.5229999999999
H	24.3309999999999	-0.6740000000000	15.3200000000000
H	26.1350000000000	-0.1560000000000	13.7110000000000
H	24.8249999999999	4.9860000000000	9.7580000000000
H	25.2270000000000	5.7000000000000	11.2989999999999
H	24.7639999999999	2.6290000000000	9.3720000000000
H	25.0320000000000	0.4180000000000	10.4199999999999
H	24.7149999999999	-0.7460000000000	6.3510000000000
P	20.9810000000000	-1.9160000000000	12.5820000000000
O	19.9869999999999	-3.0020000000000	12.7980000000000
O	20.7500000000000	-1.0630000000000	11.2200000000000
O	21.0060000000000	-0.7610000000000	13.7279999999999
C	20.9499999999999	-1.1030000000000	15.1020000000000
C	20.8629999999999	0.1820000000000	15.9420000000000
O	21.9280000000000	1.0700000000000	15.6280000000000
C	19.6589999999999	1.0460000000000	15.6289999999999
O	18.5109999999999	0.4740000000000	16.2420000000000
C	20.0599999999999	2.4190000000000	16.1430000000000
O	19.9589999999999	2.5750000000000	17.5489999999999
C	21.5459999999999	2.4220000000000	15.7319999999999
N	21.8120000000000	3.1280000000000	14.4179999999999
C	21.7880000000000	2.6470000000000	13.1549999999999
N	21.9879999999999	3.5610000000000	12.2360000000000
C	22.1200000000000	4.6980000000000	12.9350000000000
C	22.2910000000000	6.0530000000000	12.4499999999999
O	22.4220000000000	6.4460000000000	11.2919999999999
N	22.2639999999999	6.9160000000000	13.5160000000000
C	22.1149999999999	6.6020000000000	14.8559999999999
N	22.0730000000000	7.6300000000000	15.7070000000000
N	22.0079999999999	5.3910000000000	15.2829999999999
C	22.0040000000000	4.4700000000000	14.2769999999999
H	21.8159999999999	-1.6940000000000	15.3900000000000
H	20.0779999999999	-1.7290000000000	15.3000000000000
H	20.8999999999999	-0.0590000000000	17.0070000000000
H	19.4890000000000	1.0730000000000	14.5510000000000
H	19.5169999999999	3.2130000000000	15.6280000000000
H	19.0289999999999	2.6450000000000	17.7820000000000
H	22.1829999999999	2.8950000000000	16.4830000000000
H	21.6000000000000	1.6010000000000	12.9649999999999
H	22.3530000000000	7.8940000000000	13.2739999999999
H	21.9750000000000	7.4570000000000	16.6960000000000
H	22.0859999999999	8.5870000000000	15.3729999999999
H	19.9969999999999	-1.3530000000000	10.6730000000000

P	16.9939999999999	0.9640000000000	15.9019999999999
O	15.9239999999999	0.1130000000000	16.4860000000000
O	16.9750000000000	1.0540000000000	14.2810000000000
O	16.9570000000000	2.5250000000000	16.3619999999999
C	15.7149999999999	3.1990000000000	16.2689999999999
C	15.8249999999999	4.6570000000000	16.7280000000000
O	16.6140000000000	5.4020000000000	15.8089999999999
C	14.5000000000000	5.3940000000000	16.6589999999999
O	13.7330000000000	5.1090000000000	17.8249999999999
C	14.9199999999999	6.8460000000000	16.5289999999999
O	15.3309999999999	7.4660000000000	17.7379999999999
C	16.1250000000000	6.7030000000000	15.5969999999999
N	15.7721015185957	6.8719515191531	14.2077491383950
C	15.4588985174318	5.8756426909696	13.2982140087012
N	15.3228687232609	6.3140968982831	12.0727117501077
C	15.5593106695800	7.6621320815328	12.1679252329785
C	15.6728719625277	8.6702880107893	11.2016499122839
N	15.4275360745250	8.4169804362633	9.9071484539299
N	16.0283304246861	9.9146636305624	11.5906922527924
C	16.1980185221367	10.1374275123544	12.9077829788138
N	16.1205729089455	9.2734857801882	13.9333554908668
C	15.8397038558702	8.0360177551963	13.4808503621368
H	14.9920000000000	2.6800000000000	16.9020000000000
H	15.3450000000000	3.1490000000000	15.2430000000000
H	16.2620000000000	4.7260000000000	17.7259999999999
H	13.9450000000000	5.0760000000000	15.7729999999999
H	14.1570000000000	7.4240000000000	16.0259999999999
H	15.4520000000000	8.4050000000000	17.5749999999999
H	16.9130000000000	7.4140000000000	15.8469999999999
H	15.4808451889485	4.8476593083431	13.5845172055508
H	15.7118681872802	9.1166918584030	9.2393419499607
H	15.5152027301854	7.4427452454736	9.6598731207276
H	16.4206847547191	11.1689104622003	13.1756399011383
H	16.3350000000000	0.4610000000000	13.8399999999999
P	12.0980000000000	5.1620000000000	17.8410000000000
O	11.4629999999999	4.6370000000000	19.0790000000000
O	11.6750000000000	4.3760000000000	16.4849999999999
O	11.7449999999999	6.7110000000000	17.4920000000000
C	11.9580000000000	7.7310000000000	18.4510000000000
C	11.9588296680283	9.1338884725199	17.8313196610909
O	13.0163940946324	9.2055241451381	16.8408471350586
C	10.7252835685779	9.5877122706086	17.0527166178857
O	9.6966197824730	10.0257014772528	17.9573233213684
C	11.3011111393750	10.7137826890251	16.1801016675522
O	11.3931442561028	11.8763436318071	17.0003403478292
C	12.6886992839011	10.1507382303554	15.8334847978211
N	12.6535755215685	9.4909915355503	14.5217528855734
C	12.7666853489604	10.3175220279445	13.3882830069498
O	12.9745545764950	11.5565248449906	13.5832461301950

N	12.6191955790174	9.7714462394233	12.1779567996978
C	12.3333003725760	8.4446812308655	12.0626986714031
N	12.1353398593445	7.9651124682619	10.8552342980648
C	12.1964455954400	7.5923035115322	13.2025232349747
C	12.3384980209908	8.1602923715767	14.4142515199882
H	12.9350000000000	7.6130000000000	18.9200000000000
H	11.2180000000000	7.6500000000000	19.2489999999999
H	12.1459118616191	9.8622266891738	18.6337462450715
H	10.3522200964739	8.7650225952560	16.4392099859972
H	10.7372683767146	10.9272287593142	15.2628270512881
H	11.8615424044603	12.5403943308582	16.4785024012839
H	13.4436316425233	10.9411463322287	15.7699749066102
H	11.9246017846800	6.9875292321829	10.7562969793905
H	12.1609254749661	8.5717235532335	10.0411638161306
H	11.9510014659208	6.5497997406733	13.1179547150082
H	12.1947827121838	7.6574879483394	15.3649783064090
H	11.0510000000000	3.6360000000000	16.6140000000000
P	8.1709314596083	10.0325227492952	17.4325362510941
O	7.2618645105769	10.6165161213213	18.4538119417941
O	7.9940936540913	8.5253341117008	16.9857066591625
O	8.1611149436304	10.7945553024800	16.0081383869063
C	8.1296038238684	12.2330271343861	15.9675102149431
C	7.9094753762697	12.7076487263745	14.5541086820974
O	8.9834972254296	12.2532733641694	13.6915735237597
C	6.6598608598851	12.2111571913585	13.8378651180418
O	5.4985640117267	12.8704347445932	14.3593992394754
C	7.0130874509607	12.5199109526149	12.3833890391069
O	6.9291410316417	13.9244605938067	12.2041252079023
C	8.4813327568815	12.0629934010827	12.3710628342528
N	8.6111192764761	10.6396673584503	11.9945521293783
C	8.5164407794485	10.3460619981539	10.6448721750580
O	8.2698312188681	11.2182626260727	9.7863385989434
N	8.6902982806856	9.0457241502878	10.3160623533352
C	8.7482301408203	7.9556020537893	11.1995268371686
O	8.7721220764707	6.8170515673536	10.7573428040736
C	8.7964551826920	8.3337299021651	12.5868806078524
C	8.7466321755313	9.6346282477697	12.9333424028504
H	9.0808308738091	12.6223953971490	16.3504101322195
H	7.3092990935914	12.6052043194051	16.5928683131815
H	7.8829943197550	13.8080089866579	14.5753295575107
H	6.5637417020969	11.1325220224559	13.9826204528116
H	6.4079611179437	11.9906366012834	11.6366321505249
H	6.8693019268182	14.1069966174138	11.2361232556302
H	9.0577852242434	12.6529330051529	11.6519398886713
H	8.6667127270300	8.8295721032120	9.3064927290849
H	8.8797233732134	7.5712072442523	13.3435906651265
H	8.7975014069060	9.9948704144481	13.9544542100755
H	7.1741691770451	8.3502714580661	16.4878566802845
P	4.0513671553670	12.1801637107431	14.1626371160373

O	3.0253958617399	12.8955921274764	14.9623422456191
O	4.2682031647818	10.6287210165559	14.3986393406176
O	3.8148454988388	12.1397539553415	12.5716221534748
C	3.4482920665909	13.3723344189071	11.9278167261189
C	3.2010000000000	13.0770000000000	10.4580000000000
O	4.3580000000000	12.5090000000000	9.8640000000000
C	2.1680000000000	12.0090000000000	10.1539999999999
O	0.8670000000000	12.5579999999999	10.3290000000000
C	2.5130000000000	11.6140000000000	8.7250000000000
O	2.1020000000000	12.5530000000000	7.7440000000000
C	4.0490000000000	11.6180000000000	8.8240000000000
N	4.6750000000000	10.2720000000000	9.1300000000000
C	5.0020000000000	9.7390000000000	10.3260000000000
N	5.5640000000000	8.5600000000000	10.2439999999999
C	5.5660000000000	8.2880000000000	8.9310000000000
C	6.0240000000000	7.1660000000000	8.2040000000000
N	6.6600000000000	6.0750000000000	8.7900000000000
N	5.8280000000000	7.1480000000000	6.8620000000000
C	5.2300000000000	8.2050000000000	6.2960000000000
N	4.7920000000000	9.3370000000000	6.8670000000000
C	4.9880000000000	9.3170000000000	8.2070000000000
H	4.2668809987114	14.1004055848664	12.0054186884825
H	2.5371901743397	13.7697326452216	12.3889060144259
H	2.9650000000000	14.0120000000000	9.9450000000000
H	2.2940000000000	11.1590000000000	10.8279999999999
H	2.1600000000000	10.6150000000000	8.4770000000000
H	2.4220000000000	12.2669999999999	6.8840000000000
H	4.5230000000000	11.9809999999999	7.9120000000000
H	4.8180000000000	10.2780000000000	11.2420000000000
H	7.0190000000000	5.3430000000000	8.1930000000000
H	6.9770000000000	6.1620000000000	9.7450000000000
H	5.0970000000000	8.1370000000000	5.2280000000000
H	3.8043222503018	10.3078457046727	15.1910769627825
P	-0.4200000000000	11.6270000000000	10.6989999999999
O	-1.6650000000000	12.3710000000000	11.0310000000000
O	0.1250000000000	10.6660000000000	11.8879999999999
O	-0.5370000000000	10.6180000000000	9.4310000000000
C	-1.0840000000000	11.0809999999999	8.2110000000000
C	-1.0170000000000	9.9730000000000	7.1510000000000
O	0.3390000000000	9.6000000000000	6.9250000000000
C	-1.6220000000000	8.6390000000000	7.5600000000000
O	-3.0450000000000	8.6860000000000	7.5410000000000
C	-0.9720000000000	7.6590000000000	6.5890000000000
O	-1.4790000000000	7.6910000000000	5.2640000000000
C	0.4560000000000	8.2380000000000	6.5890000000000
N	1.4170000000000	7.5580000000000	7.4520000000000
C	2.0070000000000	6.4060000000000	6.9700000000000
O	1.8840000000000	5.9520000000000	5.8330000000000
N	2.7620000000000	5.7410000000000	7.8950000000000

C	2.9600000000000	5.9980000000000	9.2180000000000
O	3.6780000000000	5.2980000000000	9.9290000000000
C	2.2520000000000	7.1790000000000	9.6710000000000
C	1.5260000000000	7.8890000000000	8.7860000000000
H	-0.5450000000000	11.9629999999999	7.8630000000000
H	-2.1210000000000	11.3870000000000	8.3610000000000
H	-1.4490000000000	10.3249999999999	6.2110000000000
H	-1.3260000000000	8.4060000000000	8.5840000000000
H	-0.9730000000000	6.6440000000000	6.9780000000000
H	-1.1010000000000	6.9550000000000	4.7740000000000
H	0.9090000000000	8.2320000000000	5.5920000000000
H	3.2230000000000	4.9100000000000	7.5480000000000
H	2.3160000000000	7.4760000000000	10.7070000000000
H	0.9790000000000	8.7700000000000	9.0780000000000
H	-0.3750000000000	10.7189999999999	12.7260000000000
P	-3.9660000000000	7.5420000000000	8.2580000000000
O	-5.4220000000000	7.8480000000000	8.3160000000000
O	-3.2690000000000	7.3400000000000	9.7100000000000
O	-3.5830000000000	6.1680000000000	7.4770000000000
C	-4.1230000000000	5.9140000000000	6.1930000000000
C	-3.5680000000000	4.5880000000000	5.6530000000000
O	-2.1450000000000	4.6040000000000	5.6950000000000
C	-3.8690000000000	3.3640000000000	6.4990000000000
O	-5.2150000000000	2.9360000000000	6.3070000000000
C	-2.7990000000000	2.3770000000000	6.0500000000000
O	-3.0130000000000	1.7910000000000	4.7760000000000
C	-1.6010000000000	3.3320000000000	5.9430000000000
N	-0.7330000000000	3.3690000000000	7.1850000000000
C	-0.7720000000000	4.1930000000000	8.2550000000000
N	0.1370000000000	3.9260000000000	9.1610000000000
C	0.7640000000000	2.8420000000000	8.6800000000000
C	1.8410000000000	2.0720000000000	9.2670000000000
O	2.4890000000000	2.3070000000000	10.2840000000000
N	2.0590000000000	0.9540000000000	8.5010000000000
C	1.4310000000000	0.6010000000000	7.3210000000000
N	1.7910000000000	-0.5650000000000	6.7800000000000
N	0.5360000000000	1.3390000000000	6.7570000000000
C	0.2240000000000	2.4480000000000	7.4880000000000
H	-3.8790000000000	6.7290000000000	5.5130000000000
H	-5.2120000000000	5.8690000000000	6.2420000000000
H	-3.8990000000000	4.4330000000000	4.6230000000000
H	-3.7510000000000	3.6090000000000	7.5570000000000
H	-2.6060000000000	1.6130000000000	6.8020000000000
H	-2.3540000000000	1.1050000000000	4.6400000000000
H	-0.9400000000000	3.0790000000000	5.1100000000000
H	-1.4990000000000	4.9880000000000	8.3170000000000
H	2.7650000000000	0.3220000000000	8.8580000000000
H	1.3760000000000	-0.8630000000000	5.9100000000000
H	2.4350000000000	-1.1880000000000	7.2540000000000

H	-3.8630000000000	7.4610000000000	10.4760000000000
P	-5.9230000000000	1.8080000000000	7.2540000000000
O	-7.3690000000000	1.5730000000000	6.9880000000000
O	-5.5980000000000	2.3240000000000	8.7590000000000
O	-4.9720000000000	0.4980000000000	7.1090000000000
C	-5.1260000000000	-0.3970000000000	6.0220000000000
C	-4.2170000000000	-1.6190000000000	6.2320000000000
O	-2.8680000000000	-1.2100000000000	6.4430000000000
C	-4.4920000000000	-2.4090000000000	7.5010000000000
O	-5.6590000000000	-3.2080000000000	7.3380000000000
C	-3.1940000000000	-3.1800000000000	7.7210000000000
O	-3.0040000000000	-4.2990000000000	6.8720000000000
C	-2.1880000000000	-2.0850000000000	7.3170000000000
N	-1.5570000000000	-1.3600000000000	8.4160000000000
C	-0.5030000000000	-1.9710000000000	9.0690000000000
O	-0.0070000000000	-3.0600000000000	8.7820000000000
N	-0.0230000000000	-1.2550000000000	10.1310000000000
C	-0.4630000000000	-0.0840000000000	10.6699999999999
O	0.0920000000000	0.4610000000000	11.6199999999999
C	-1.6390000000000	0.4370000000000	10.0030000000000
C	-2.1270000000000	-0.2160000000000	8.9320000000000
H	-4.8890000000000	0.1000000000000	5.0830000000000
H	-6.1640000000000	-0.7220000000000	5.9420000000000
H	-4.2610000000000	-2.2740000000000	5.3600000000000
H	-4.6720000000000	-1.7190000000000	8.3280000000000
H	-3.0620000000000	-3.4560000000000	8.7650000000000
H	-2.2230000000000	-4.7770000000000	7.1650000000000
H	-1.3540000000000	-2.4800000000000	6.7290000000000
H	0.7700000000000	-1.6750000000000	10.5999999999999
H	-2.1100000000000	1.3360000000000	10.3719999999999
H	-3.0040000000000	0.1240000000000	8.4100000000000
H	-6.3800000000000	2.4700000000000	9.3240000000000
P	-6.4500000000000	-3.8690000000000	8.6050000000000
O	-7.7240000000000	-4.5590000000000	8.2680000000000
O	-6.6130000000000	-2.6290000000000	9.6400000000000
O	-5.3320000000000	-4.8010000000000	9.3280000000000
C	-5.0340000000000	-6.0880000000000	8.8170000000000
C	-3.9870000000000	-6.7640000000000	9.7160000000000
O	-2.8340000000000	-5.9380000000000	9.8390000000000
C	-4.4010000000000	-6.9180000000000	11.1699999999999
O	-5.2860000000000	-8.0240000000000	11.2929999999999
C	-3.0760000000000	-7.0910000000000	11.8979999999999
O	-2.5040000000000	-8.3860000000000	11.8100000000000
C	-2.2110000000000	-6.1020000000000	11.0899999999999
N	-1.9470000000000	-4.8120000000000	11.7279999999999
C	-0.9560000000000	-4.7320000000000	12.6950000000000
O	-0.3300000000000	-5.7480000000000	13.0050000000000
N	-0.7550000000000	-3.4670000000000	13.2799999999999
C	-1.5560000000000	-2.4830000000000	13.0839999999999

N	-1.3550000000000	-1.3070000000000	13.6820000000000
C	-2.6830000000000	-2.5990000000000	12.2089999999999
C	-2.8270000000000	-3.7670000000000	11.5670000000000
H	-4.6690000000000	-6.0190000000000	7.7930000000000
H	-5.9370000000000	-6.6990000000000	8.7810000000000
H	-3.6930000000000	-7.7270000000000	9.2950000000000
H	-4.9070000000000	-6.0120000000000	11.5090000000000
H	-3.1540000000000	-6.7690000000000	12.9350000000000
H	-1.7320000000000	-8.4180000000000	12.3800000000000
H	-1.2220000000000	-6.5070000000000	10.8520000000000
H	-1.9740000000000	-0.5290000000000	13.5150000000000
H	-0.5570000000000	-1.1760000000000	14.2910000000000
H	-3.3920000000000	-1.8000000000000	12.0570000000000
H	-3.6440000000000	-3.9570000000000	10.8900000000000
H	-7.5310000000000	-2.4280000000000	9.9020000000000
P	-6.4270000000000	-8.0960000000000	12.4580000000000
O	-7.3770000000000	-9.2340000000000	12.3379999999999
O	-7.1250000000000	-6.6310000000000	12.4060000000000
O	-5.5690000000000	-8.0540000000000	13.8399999999999
C	-4.8840000000000	-9.2200000000000	14.2669999999999
C	-4.0130000000000	-8.9210000000000	15.4969999999999
O	-3.0800000000000	-7.8950000000000	15.1880000000000
C	-4.7370000000000	-8.3370000000000	16.7079999999999
O	-5.4690000000000	-9.3000000000000	17.4460000000000
C	-3.6070000000000	-7.6640000000000	17.4879999999999
O	-2.8630000000000	-8.5370000000000	18.3270000000000
C	-2.7120000000000	-7.1690000000000	16.3329999999999
N	-2.7560000000000	-5.7280000000000	16.0749999999999
C	-1.9470000000000	-4.8850000000000	16.8290000000000
O	-1.1060000000000	-5.3620000000000	17.5919999999999
N	-2.1830000000000	-3.5020000000000	16.6879999999999
C	-3.1870000000000	-3.0370000000000	16.0369999999999
N	-3.4460000000000	-1.7270000000000	16.0300000000000
C	-4.0650000000000	-3.8960000000000	15.3000000000000
C	-3.8030000000000	-5.2090000000000	15.3490000000000
H	-4.2580000000000	-9.6030000000000	13.4600000000000
H	-5.6010000000000	-10.0069999999999	14.5009999999999
H	-3.4580000000000	-9.8170000000000	15.7829999999999
H	-5.4300000000000	-7.5620000000000	16.3719999999999
H	-4.8650000000000	-9.9880000000000	17.7349999999999
H	-3.9830000000000	-6.8200000000000	18.0700000000000
H	-3.3750000000000	-8.7070000000000	19.1219999999999
H	-1.6570000000000	-7.4160000000000	16.4810000000000
H	-4.2400000000000	-1.3560000000000	15.5320000000000
H	-2.8340000000000	-1.1000000000000	16.5380000000000
H	-4.8970000000000	-3.5240000000000	14.7210000000000
H	-4.4050000000000	-5.9400000000000	14.8279999999999
H	-8.0900000000000	-6.6380000000000	12.2609999999999

N	11.8587181850707	10.1712812369028	8.3478650923198
C	11.4143486258919	11.3835948678980	8.7706143513699
N	11.3972606907640	11.5665875792743	10.1189630404816
N	10.9390677736269	12.3749383517680	7.9819964446875
C	10.9145179308177	12.0289641310125	6.6823281078994
C	11.3851591879545	10.8466800794522	6.1303326390058
C	11.8623718795887	9.9020733746312	7.0437694422126
C	12.6901834816239	7.7010318460779	7.4866007243732
O	12.2658540866709	8.7541493831534	6.5699184582261
N	11.1558005493004	10.8015166814281	4.7676239720022
C	10.5548565475938	11.9435210164919	4.5096029098851
N	10.3842106595078	12.7304756428694	5.6294785769282
H	10.0200209749184	13.6884701988233	5.6979319772493
H	11.1972697370060	12.4957469999734	10.4525689118644
H	11.9980065170736	10.9887753456513	10.7173790494393
H	13.1322210289713	6.9750009891106	6.8031795213654
H	13.4157121743916	8.0932736296880	8.2012860331685
H	11.7956181977421	7.3029459634539	7.9682051360677
H	10.1627073907373	12.2322546794429	3.5458134202826
